# Supplementary material for: Centriflaken: An automated data analysis pipeline for assembly and in silico analyses of foodborne pathogens from metagenomic samples
Source: PLoS One. 2025 Dec 23;20(12):e0329425. doi: 10.1371/journal.pone.0329425 (PMC12725755; doi:10.1371/journal.pone.0329425)
Supplement: S3 File — (HTML) [file pone.0329425.s003.html]

CPIPES Report: MultiQC Report


# Toggle navigation v1.12

# CPIPES Report

Loading report..

- General Stats
- FastQC
  - Sequence Counts
  - Sequence Quality Histograms
  - Per Sequence Quality Scores
  - Per Base Sequence Content
  - Per Sequence GC Content
  - Per Base N Content
  - Sequence Length Distribution
  - Sequence Duplication Levels
  - Overrepresented sequences
  - Adapter Content
  - Status Checks
- Centrifuge
- Kraken2
- SEROTYPEFINDER
- ABRICATE\_NCBIAMRPLUS
- ABRICATE\_MEGARES
- ABRICATE\_RESFINDER
- ABRICATE\_ARGANNOT
- CPIPES Software Versions

Toolbox

### MultiQC Toolbox

#### Apply Highlight Samples

+

Regex mode off
help
 Clear

#### Apply Rename Samples

+

Click here for bulk input.

Paste two columns of a tab-delimited table here (eg. from Excel).

First column should be the old name, second column the new name.

Add

Regex mode off
help
 Clear

#### Apply Show / Hide Samples

Hide matching samples

Show only matching samples

+

Regex mode off
help
 Clear

#### Export Plots

- Images
- Data

px

px

Aspect ratio

PNG
JPEG
SVG

Plot scaling

X

Download the raw data used to create the plots in this report below:

Format:

Tab-separated
Comma-separated
JSON

Note that additional data was saved in `multiqc_data` when this report was generated.

---

##### Choose Plots

 All
 None

---


   Download Plot Images

If you use plots from MultiQC in a publication or presentation, please cite:

> **MultiQC: Summarize analysis results for multiple tools and samples in a single report**  
> *Philip Ewels, Måns Magnusson, Sverker Lundin and Max Käller*  
> Bioinformatics (2016)  
> doi: 10.1093/bioinformatics/btw354  
> PMID: 27312411

#### Save Settings

You can save the toolbox settings for this report to the browser.

 Save


---

#### Load Settings

Choose a saved report profile from the dropdown box below:

[ select ]

Load
 Delete
 Set default
 Clear default

#### Tool Citations

Please remember to cite the tools that you use in your analysis.

To help with this, you can download publication details of the tools mentioned in this report:

List of DOIs

BibTeX file

#### About MultiQC

This report was generated using MultiQC, version 1.12

You can see a YouTube video describing how to use MultiQC reports here:
https://youtu.be/qPbIlO\_KWN0

For more information about MultiQC, including other videos and
extensive documentation, please visit http://multiqc.info

You can report bugs, suggest improvements and find the source code for MultiQC on GitHub:
https://github.com/ewels/MultiQC

MultiQC is published in Bioinformatics:

> **MultiQC: Summarize analysis results for multiple tools and samples in a single report**  
> *Philip Ewels, Måns Magnusson, Sverker Lundin and Max Käller*  
> Bioinformatics (2016)  
> doi: 10.1093/bioinformatics/btw354  
> PMID: 27312411

# 

# CPIPES Report

CPIPES (CFSAN PIPELINES) is a modular bioinformatics data analysis project at CFSAN, FDA based on NEXTFLOW DSL2.

> This report has been generated by the CPIPES - centriflaken analysis pipeline. Only certain tables and plots are reported here. For complete results, please refer to the analysis pipeline output directory.

CPIPES Version
:   0.4.0

Workflow
:   centriflaken

Workflow Version
:   0.2.1

Input Directory
:   /nfs/legacy/projects/NGE-nanopore-projects/runs\_2022/samples\_21\_Yuma

Output Directory
:   /nfs/legacy/projects/NGE-nanopore-projects/runs\_2022/samples\_21\_Yuma/samples

Developer E-mail
:   Kranti.Konganti@fda.hhs.gov

Stakeholder E-mail
:   Narjol.Gonzalez-Escalona@fda.hhs.gov

#### JavaScript Disabled

MultiQC reports use JavaScript for plots and toolbox functions. It looks like
you have JavaScript disabled in your web browser. Please note that many of the report
functions will not work as intended.

Loading report..

---

×
don't show again

**Welcome!** Not sure where to start?  
Watch a tutorial video
  *(6:06)*

## General Statistics

 Copy table

 Configure Columns

 Sort by highlight

 Plot
Showing 21/21 rows and 4/11 columns.

| Sample Name | % Dups | % GC | Read Length | % Failed | M Seqs | % Escherichia coli | % Top 5 Species | % Unclassified | % Escherichia coli | % Top 5 Species | % Unclassified |
| --- | --- | --- | --- | --- | --- | --- | --- | --- | --- | --- | --- |
| FAL00958 | 0.0% | 53% | 4748 bp | 30% | 2.0 | 18.3% | 58.6% | 1.5% | 89.2% | 95.7% |  |
| FAL01198 | 0.0% | 50% | 5391 bp | 30% | 2.5 | 40.1% | 60.1% | 2.5% | 86.7% | 91.4% | 0.1% |
| FAL01556 | 0.0% | 53% | 5256 bp | 30% | 2.5 | 25.8% | 65.4% | 2.8% | 72.6% | 92.3% | 0.1% |
| FAL05306 | 0.0% | 52% | 4807 bp | 30% | 2.3 | 22.3% | 59.1% | 3.9% | 83.6% | 92.6% | 0.1% |
| FAL11145 | 0.0% | 52% | 4577 bp | 30% | 1.5 | 24.0% | 59.3% | 2.1% | 89.3% | 94.8% |  |
| FAL11151 | 0.0% | 52% | 5452 bp | 30% | 1.3 | 22.5% | 57.8% | 2.1% | 88.0% | 94.7% | 0.2% |
| FAL11305 | 0.0% | 53% | 5451 bp | 30% | 2.7 | 27.2% | 69.4% | 2.8% | 69.8% | 90.5% |  |
| FAL11351 | 0.0% | 52% | 4628 bp | 30% | 2.1 | 21.4% | 51.2% | 2.9% | 84.2% | 93.5% |  |
| FAL11384 | 0.0% | 50% | 5043 bp | 30% | 2.6 | 37.0% | 56.7% | 3.5% | 83.5% | 90.2% | 0.2% |
| FAL11387 | 0.0% | 52% | 5312 bp | 30% | 2.6 | 22.0% | 83.9% | 1.4% | 82.6% | 94.1% |  |
| FAL11417 | 0.0% | 52% | 5366 bp | 30% | 1.9 | 27.7% | 64.5% | 2.8% | 74.2% | 91.4% |  |
| FAL11425 | 0.0% | 54% | 4687 bp | 40% | 2.0 | 10.8% | 62.3% | 1.8% | 75.7% | 92.9% | 0.3% |
| FAL16059 | 0.0% | 52% | 5150 bp | 30% | 2.1 | 22.5% | 55.1% | 2.5% | 71.6% | 91.8% |  |
| FAL17459 | 0.0% | 53% | 5671 bp | 30% | 2.5 | 20.7% | 64.2% | 2.8% | 84.3% | 93.5% |  |
| FAO71993 | 0.0% | 52% | 4928 bp | 30% | 2.3 | 24.9% | 48.6% | 2.1% | 85.8% | 93.3% |  |
| FAO72169 | 0.0% | 52% | 4262 bp | 30% | 2.0 | 23.1% | 55.4% | 3.1% | 88.6% | 95.0% |  |
| FAO96374 | 0.0% | 52% | 4252 bp | 30% | 1.7 | 25.4% | 63.0% | 2.4% | 87.5% | 94.6% |  |
| FAO97359 | 0.0% | 51% | 6177 bp | 30% | 2.8 | 22.7% | 63.1% | 1.9% | 82.6% | 92.8% |  |
| FAQ33890 | 0.0% | 52% | 5289 bp | 30% | 2.2 | 25.9% | 47.0% | 2.3% | 88.3% | 92.2% |  |
| FAQ33923 | 0.0% | 52% | 4759 bp | 30% | 2.4 | 32.4% | 61.4% | 2.5% | 87.8% | 93.5% |  |
| FAQ34034 | 0.0% | 52% | 4917 bp | 30% | 2.1 | 25.5% | 70.3% | 1.6% | 83.1% | 94.9% |  |

×

#### General Statistics: Columns

Uncheck the tick box to hide columns. Click and drag the handle on the left to change order.

Show All
Show None

| Sort | Visible | Group | Column | Description | ID | Scale |
| --- | --- | --- | --- | --- | --- | --- |
| || |  | FastQC | % Dups | % Duplicate Reads | `percent_duplicates` | None |
| || |  | FastQC | % GC | Average % GC Content | `percent_gc` | None |
| || |  | FastQC | Read Length | Average Read Length (bp) | `avg_sequence_length` | None |
| || |  | FastQC | % Failed | Percentage of modules failed in FastQC report (includes those not plotted here) | `percent_fails` | None |
| || |  | FastQC | M Seqs | Total Sequences (millions) | `total_sequences` | read\_count |
| || |  | Centrifuge | % Escherichia coli | Percentage of reads that were the top Species over all samples - Escherichia coli | `% Escherichia coli` | None |
| || |  | Centrifuge | % Top 5 Species | Percentage of reads that were classified by one of the top 5 Species (Escherichia coli, Klebsiella pneumoniae, Vibrio cholerae, Enterobacter cloacae complex, Enterobacter cloacae) | `% Top 5` | None |
| || |  | Centrifuge | % Unclassified | Percentage of reads that were unclassified | `% Unclassified` | None |
| || |  | Kraken2 | % Escherichia coli | Percentage of reads that were the top Species over all samples - Escherichia coli | `% Escherichia coli` | None |
| || |  | Kraken2 | % Top 5 Species | Percentage of reads that were classified by one of the top 5 Species (Escherichia coli, Klebsiella pneumoniae, Salmonella enterica, Escherichia albertii, Escherichia fergusonii) | `% Top 5` | None |
| || |  | Kraken2 | % Unclassified | Percentage of reads that were unclassified | `% Unclassified` | None |

Close

## FastQC

FastQC section of the report shows FastQC results **before** adapter trimming.

### Sequence Counts Help

Sequence counts for each sample. Duplicate read counts are an estimate only.

This plot show the total number of reads, broken down into unique and duplicate
if possible (only more recent versions of FastQC give duplicate info).

You can read more about duplicate calculation in the
FastQC documentation.
A small part has been copied here for convenience:

*Only sequences which first appear in the first 100,000 sequences
in each file are analysed. This should be enough to get a good impression
for the duplication levels in the whole file. Each sequence is tracked to
the end of the file to give a representative count of the overall duplication level.*

*The duplication detection requires an exact sequence match over the whole length of
the sequence. Any reads over 75bp in length are truncated to 50bp for this analysis.*

Number of reads
Percentages

loading..

---

### Sequence Quality Histograms Help

The mean quality value across each base position in the read.

To enable multiple samples to be plotted on the same graph, only the mean quality
scores are plotted (unlike the box plots seen in FastQC reports).

Taken from the FastQC help:

*The y-axis on the graph shows the quality scores. The higher the score, the better
the base call. The background of the graph divides the y axis into very good quality
calls (green), calls of reasonable quality (orange), and calls of poor quality (red).
The quality of calls on most platforms will degrade as the run progresses, so it is
common to see base calls falling into the orange area towards the end of a read.*

loading..

---

### Per Sequence Quality Scores Help

The number of reads with average quality scores. Shows if a subset of reads has poor quality.

From the FastQC help:

*The per sequence quality score report allows you to see if a subset of your
sequences have universally low quality values. It is often the case that a
subset of sequences will have universally poor quality, however these should
represent only a small percentage of the total sequences.*

loading..

---

### Per Base Sequence Content Help

The proportion of each base position for which each of the four normal DNA bases has been called.

To enable multiple samples to be shown in a single plot, the base composition data
is shown as a heatmap. The colours represent the balance between the four bases:
an even distribution should give an even muddy brown colour. Hover over the plot
to see the percentage of the four bases under the cursor.

**To see the data as a line plot, as in the original FastQC graph, click on a sample track.**

From the FastQC help:

*Per Base Sequence Content plots out the proportion of each base position in a
file for which each of the four normal DNA bases has been called.*

*In a random library you would expect that there would be little to no difference
between the different bases of a sequence run, so the lines in this plot should
run parallel with each other. The relative amount of each base should reflect
the overall amount of these bases in your genome, but in any case they should
not be hugely imbalanced from each other.*

*It's worth noting that some types of library will always produce biased sequence
composition, normally at the start of the read. Libraries produced by priming
using random hexamers (including nearly all RNA-Seq libraries) and those which
were fragmented using transposases inherit an intrinsic bias in the positions
at which reads start. This bias does not concern an absolute sequence, but instead
provides enrichement of a number of different K-mers at the 5' end of the reads.
Whilst this is a true technical bias, it isn't something which can be corrected
by trimming and in most cases doesn't seem to adversely affect the downstream
analysis.*

Click a sample row to see a line plot for that dataset.

##### Rollover for sample name

 Export Plot

Position: -

%T: -

%C: -

%A: -

%G: -

---

### Per Sequence GC Content Help

The average GC content of reads. Normal random library typically have a
roughly normal distribution of GC content.

From the FastQC help:

*This module measures the GC content across the whole length of each sequence
in a file and compares it to a modelled normal distribution of GC content.*

*In a normal random library you would expect to see a roughly normal distribution
of GC content where the central peak corresponds to the overall GC content of
the underlying genome. Since we don't know the the GC content of the genome the
modal GC content is calculated from the observed data and used to build a
reference distribution.*

*An unusually shaped distribution could indicate a contaminated library or
some other kinds of biased subset. A normal distribution which is shifted
indicates some systematic bias which is independent of base position. If there
is a systematic bias which creates a shifted normal distribution then this won't
be flagged as an error by the module since it doesn't know what your genome's
GC content should be.*

Percentages
Counts

loading..

---

### Per Base N Content Help

The percentage of base calls at each position for which an `N` was called.

From the FastQC help:

*If a sequencer is unable to make a base call with sufficient confidence then it will
normally substitute an `N` rather than a conventional base call. This graph shows the
percentage of base calls at each position for which an `N` was called.*

*It's not unusual to see a very low proportion of Ns appearing in a sequence, especially
nearer the end of a sequence. However, if this proportion rises above a few percent
it suggests that the analysis pipeline was unable to interpret the data well enough to
make valid base calls.*

loading..

---

### Sequence Length Distribution

The distribution of fragment sizes (read lengths) found.
See the FastQC help

loading..

---

### Sequence Duplication Levels Help

The relative level of duplication found for every sequence.

From the FastQC Help:

*In a diverse library most sequences will occur only once in the final set.
A low level of duplication may indicate a very high level of coverage of the
target sequence, but a high level of duplication is more likely to indicate
some kind of enrichment bias (eg PCR over amplification). This graph shows
the degree of duplication for every sequence in a library: the relative
number of sequences with different degrees of duplication.*

*Only sequences which first appear in the first 100,000 sequences
in each file are analysed. This should be enough to get a good impression
for the duplication levels in the whole file. Each sequence is tracked to
the end of the file to give a representative count of the overall duplication level.*

*The duplication detection requires an exact sequence match over the whole length of
the sequence. Any reads over 75bp in length are truncated to 50bp for this analysis.*

*In a properly diverse library most sequences should fall into the far left of the
plot in both the red and blue lines. A general level of enrichment, indicating broad
oversequencing in the library will tend to flatten the lines, lowering the low end
and generally raising other categories. More specific enrichments of subsets, or
the presence of low complexity contaminants will tend to produce spikes towards the
right of the plot.*

loading..

---

### Overrepresented sequences Help

The total amount of overrepresented sequences found in each library.

FastQC calculates and lists overrepresented sequences in FastQ files. It would not be
possible to show this for all samples in a MultiQC report, so instead this plot shows
the *number of sequences* categorized as over represented.

Sometimes, a single sequence may account for a large number of reads in a dataset.
To show this, the bars are split into two: the first shows the overrepresented reads
that come from the single most common sequence. The second shows the total count
from all remaining overrepresented sequences.

From the FastQC Help:

*A normal high-throughput library will contain a diverse set of sequences, with no
individual sequence making up a tiny fraction of the whole. Finding that a single
sequence is very overrepresented in the set either means that it is highly biologically
significant, or indicates that the library is contaminated, or not as diverse as you expected.*

*FastQC lists all of the sequences which make up more than 0.1% of the total.
To conserve memory only sequences which appear in the first 100,000 sequences are tracked
to the end of the file. It is therefore possible that a sequence which is overrepresented
but doesn't appear at the start of the file for some reason could be missed by this module.*

21 samples had less than 1% of reads made up of overrepresented sequences

---

### Adapter Content Help

The cumulative percentage count of the proportion of your
library which has seen each of the adapter sequences at each position.

Note that only samples with ≥ 0.1% adapter contamination are shown.

There may be several lines per sample, as one is shown for each adapter
detected in the file.

From the FastQC Help:

*The plot shows a cumulative percentage count of the proportion
of your library which has seen each of the adapter sequences at each position.
Once a sequence has been seen in a read it is counted as being present
right through to the end of the read so the percentages you see will only
increase as the read length goes on.*

No samples found with any adapter contamination > 0.1%

---

### Status Checks Help

Status for each FastQC section showing whether results seem entirely normal (green),
slightly abnormal (orange) or very unusual (red).

FastQC assigns a status for each section of the report.
These give a quick evaluation of whether the results of the analysis seem
entirely normal (green), slightly abnormal (orange) or very unusual (red).

It is important to stress that although the analysis results appear to give a pass/fail result,
these evaluations must be taken in the context of what you expect from your library.
A 'normal' sample as far as FastQC is concerned is random and diverse.
Some experiments may be expected to produce libraries which are biased in particular ways.
You should treat the summary evaluations therefore as pointers to where you should concentrate
your attention and understand why your library may not look random and diverse.

Specific guidance on how to interpret the output of each module can be found in the relevant
report section, or in the FastQC help.

In this heatmap, we summarise all of these into a single heatmap for a quick overview.
Note that not all FastQC sections have plots in MultiQC reports, but all status checks
are shown in this heatmap.

Sort by highlight

Min:

Max:

loading..

---

## Centrifuge

Centrifuge section of the report shows how **reads** are classified. Please note that the plot title below is shown as **Kraken2: Top taxa** since `centrifuge-kreport` was used to create Kraken-style reports from centrifuge output files.
.*DOI: 10.1101/gr.210641.116.*

### Top taxa Help

The number of reads falling into the top 5 taxa across different ranks.

To make this plot, the percentage of each sample assigned to a given taxa is summed across all samples.
The counts for these top five taxa are then plotted for each of the 9 different taxa ranks.
The unclassified count is always shown across all taxa ranks.

The total number of reads is approximated by dividing the number of `unclassified` reads by the percentage of
the library that they account for.
Note that this is only an approximation, and that kraken percentages don't always add to exactly 100%.

The category *"Other"* shows the difference between the above total read count and the sum of the read counts
in the top 5 taxa shown + unclassified. This should cover all taxa *not* in the top 5, +/- any rounding errors.

Note that any taxon that does not exactly fit a taxon rank (eg. `-` or `G2`) is ignored.

Counts
Percentages

Species
Genus
Family
Order
Class
Phylum
Kingdom

loading..

---

## Kraken2

Kraken2 section of the report shows how **assembled contigs** are classified.*DOI: 10.1186/gb-2014-15-3-r46.*

### Top taxa Help

The number of reads falling into the top 5 taxa across different ranks.

To make this plot, the percentage of each sample assigned to a given taxa is summed across all samples.
The counts for these top five taxa are then plotted for each of the 9 different taxa ranks.
The unclassified count is always shown across all taxa ranks.

The total number of reads is approximated by dividing the number of `unclassified` reads by the percentage of
the library that they account for.
Note that this is only an approximation, and that kraken percentages don't always add to exactly 100%.

The category *"Other"* shows the difference between the above total read count and the sum of the read counts
in the top 5 taxa shown + unclassified. This should cover all taxa *not* in the top 5, +/- any rounding errors.

Note that any taxon that does not exactly fit a taxon rank (eg. `-` or `G2`) is ignored.

Counts
Percentages

Species
Genus
Family
Order
Class
Phylum
Kingdom
Domain
Root

loading..

---

## SEROTYPEFINDER

The results table shown here is a collection from all samples.

| Name | Database | Gene | Serotype | Identity | Template / HSP length | Contig | Position in contig | Accession number |
| --- | --- | --- | --- | --- | --- | --- | --- | --- |
| `FAL11425` | H\_type | fliC | H7 | 99.94 | 1758 / 1758 | contig\_237 | 2034..3790 | AF228496 |
| `FAL11425` | H\_type | fliC | H8 | 98.99 | 1479 / 1479 | contig\_238 | 1719..3197 | AJ865465 |
| `FAL11425` | H\_type | fliC | H8 | 100.0 | 1479 / 1479 | contig\_201 | 345549..347027 | AJ884569 |
| `FAL11425` | O\_type | wzx | O153 | 99.58 | 1179 / 1179 | contig\_275 | 1579..2753 | KJ755551 |
| `FAL11425` | O\_type | wzy | O153 | 99.91 | 1167 / 1167 | contig\_275 | 3709..4874 | KJ755551 |
| `FAL11425` | O\_type | wzx | O170 | 96.98 | 1259 / 1248 | contig\_277 | 5808..7055 | AB812070 |
| `FAL11425` | O\_type | wzy | O170 | 99.15 | 1174 / 1173 | contig\_277 | 1811..2981 | AB812070 |
| `FAL11425` | O\_type | wzy | O178 | 99.82 | 1098 / 1098 | contig\_273 | 2040..3135 | KJ778799 |
| `FAL11425` | O\_type | wzx | O185 | 97.92 | 1441 / 1434 | contig\_276 | 2100..3537 | AB812081 |
| `FAL11425` | O\_type | wzy | O185 | 97.96 | 1373 / 1371 | contig\_276 | 4427..5790 | AB812081 |
| `FAL11425` | O\_type | wzm | O8 | 99.37 | 795 / 795 | contig\_290 | 6193..6986 | AB010150 |
| `FAL11425` | O\_type | wzt | O8 | 99.59 | 1216 / 1215 | contig\_271 | 7936..9150 | AB811598 |
| `FAL11425` | O\_type | wzx | O81 | 99.39 | 1302 / 1302 | contig\_274 | 6276..7569 | CU928162 |
| `FAL11425` | O\_type | wzy | O81 | 99.51 | 1230 / 1230 | contig\_274 | 3109..4332 | CU928162 |
| `FAL11425` | O\_type | wzx | O82 | 99.76 | 1230 / 1230 | contig\_269 | 43690..44917 | AB812034 |
| `FAL11425` | O\_type | wzy | O82 | 99.54 | 1083 / 1083 | contig\_269 | 41442..42521 | AB812034 |
| `FAL11425` | O\_type | wzy | O93 | 98.23 | 1525 / 1512 | contig\_280 | 5561..7078 | AB812041 |
| `FAL11145` | H\_type | fliC | H21 | 98.04 | 1481 / 1476 | contig\_378 | 64..1543 | AIHL01000060 |
| `FAL11145` | H\_type | flkA | H36 | 96.57 | 1689 / 1671 | contig\_248 | 4076..5755 | EF392693 |
| `FAL11145` | H\_type | fliC | H7 | 97.72 | 1758 / 1758 | contig\_377 | 186..1942 | AF228496 |
| `FAL11145` | H\_type | fliC | H8 | 96.86 | 1495 / 1479 | contig\_364 | 351556..353050 | AJ884569 |
| `FAL11145` | O\_type | wzx | O10 | 98.69 | 1528 / 1521 | contig\_343 | 9307..10825 | AB811599 |
| `FAL11145` | O\_type | wzy | O10 | 99.04 | 1043 / 1044 | contig\_343 | 5483..6519 | AB811599 |
| `FAL11145` | O\_type | wzx | O110 | 99.84 | 1251 / 1251 | contig\_45 | 212663..213911 | AB812049 |
| `FAL11145` | O\_type | wzy | O110 | 99.59 | 1206 / 1206 | contig\_45 | 215030..216230 | AB812049 |
| `FAL11145` | O\_type | wzy | O123 | 99.05 | 1257 / 1251 | contig\_335 | 8247..9497 | AB972419 |
| `FAL11145` | O\_type | wzx | O123 | 90.57 | 1527 / 1446 | contig\_335 | 1863..3382 | DQ676934 |
| `FAL11145` | O\_type | wzy | O123/O186 | 99.05 | 1257 / 1251 | contig\_335 | 8247..9497 | DQ676934-AB812082 |
| `FAL11145` | O\_type | wzx | O128ab | 99.1 | 1444 / 1443 | contig\_330 | 5184..6621 | AY217096 |
| `FAL11145` | O\_type | wzx | O128ac | 99.1 | 1444 / 1443 | contig\_330 | 5184..6621 | SAMEA3529399 |
| `FAL11145` | O\_type | wzy | O128ac | 99.14 | 1044 / 1044 | contig\_330 | 1577..2612 | SAMEA3529399 |
| `FAL11145` | O\_type | wzy | O139 | 97.75 | 1247 / 1239 | contig\_339 | 5127..6363 | DQ109552 |
| `FAL11145` | O\_type | wzx | O139 | 98.12 | 1223 / 1221 | contig\_339 | 3065..4274 | DQ109552 |
| `FAL11145` | O\_type | wzx | O148 | 99.66 | 1191 / 1191 | contig\_338 | 4095..5282 | AAJT02000037 |
| `FAL11145` | O\_type | wzx | O15 | 96.37 | 1268 / 1245 | contig\_345 | 7288..8542 | AY647261 |
| `FAL11145` | O\_type | wzy | O15 | 95.73 | 1218 / 1179 | contig\_345 | 5038..6249 | AY647261 |
| `FAL11145` | O\_type | wzm | O162 | 99.74 | 782 / 780 | contig\_351 | 23787..24568 | AB812067 |
| `FAL11145` | O\_type | wzt | O162 | 99.6 | 754 / 753 | contig\_351 | 23029..23780 | AB812067 |
| `FAL11145` | O\_type | wzx | O17/O77 | 98.55 | 1238 / 1236 | contig\_333 | 7092..8317 | AB812084-DQ000314 |
| `FAL11145` | O\_type | wzy | O18 | 98.99 | 1192 / 1191 | contig\_342 | 4363..5549 | GU299793 |
| `FAL11145` | O\_type | wzy | O186 | 99.05 | 1257 / 1251 | contig\_335 | 8247..9497 | AB812082 |
| `FAL11145` | O\_type | wzx | O186 | 90.57 | 1527 / 1446 | contig\_335 | 1863..3382 | KP710595 |
| `FAL11145` | O\_type | wzx | O18ac | 98.48 | 1249 / 1248 | contig\_342 | 5606..6843 | GU299793 |
| `FAL11145` | O\_type | wzx | O20 | 98.59 | 1415 / 1410 | contig\_340 | 10976..12380 | KJ778793 |
| `FAL11145` | O\_type | wzy | O20 | 98.2 | 1335 / 1323 | contig\_340 | 8898..10222 | KJ778793 |
| `FAL11145` | O\_type | wzx | O21 | 94.35 | 1256 / 1236 | contig\_346 | 4278..5510 | EU694098 |
| `FAL11145` | O\_type | wzy | O21 | 97.99 | 1092 / 1083 | contig\_346 | 910..1991 | EU694098 |
| `FAL11145` | O\_type | wzx | O3 | 98.41 | 1505 / 1494 | contig\_344 | 6630..8126 | EU694097 |
| `FAL11145` | O\_type | wzy | O3 | 98.9 | 1088 / 1086 | contig\_344 | 4415..5493 | EU694097 |
| `FAL11145` | O\_type | wzx | O33 | 98.72 | 1247 / 1245 | contig\_329 | 5270..6506 | AB811611 |
| `FAL11145` | O\_type | wzy | O33 | 98.34 | 1204 / 1203 | contig\_329 | 7647..8839 | AB811611 |
| `FAL11145` | O\_type | wzy | O55 | 93.7 | 1000 / 978 | contig\_331 | 15345..16329 | CP003109 |
| `FAL11145` | O\_type | wzx | O55 | 95.28 | 1313 / 1278 | contig\_331 | 14026..15327 | JH958641 |
| `FAL11145` | O\_type | wzy | O6 | 97.0 | 1367 / 1344 | contig\_332 | 1314..2674 | CP002185 |
| `FAL11145` | O\_type | wzx | O6 | 96.73 | 1253 / 1239 | contig\_332 | 78..1311 | CP002185 |
| `FAL11145` | O\_type | wzx | O7 | 98.2 | 1441 / 1431 | contig\_609 | 399..1831 | CP003034 |
| `FAL11145` | O\_type | wzy | O73 | 97.48 | 1350 / 1332 | contig\_333 | 5711..7050 | DQ000313 |
| `FAL11145` | O\_type | wzy | O75 | 97.69 | 1084 / 1074 | contig\_331 | 37904..38983 | GU299795 |
| `FAL11145` | O\_type | wzt | O8 | 98.77 | 1221 / 1215 | contig\_45 | 226188..227405 | AB811598 |
| `FAL11145` | O\_type | wzx | O82 | 98.86 | 1232 / 1230 | contig\_334 | 2231..3453 | AB812034 |
| `FAL11145` | O\_type | wzy | O82 | 97.98 | 1089 / 1083 | contig\_334 | 4620..5700 | AB812034 |
| `FAL11145` | O\_type | wzm | O89 | 95.59 | 793 / 780 | contig\_352 | 29612..30397 | AB812038 |
| `FAL11145` | O\_type | wzt | O89 | 94.05 | 773 / 753 | contig\_352 | 28841..29605 | AB812038 |
| `FAL11145` | O\_type | wzm | O9 | 94.23 | 797 / 786 | contig\_525 | 6086..6873 | D43637 |
| `FAL11145` | O\_type | wzt | O9a | 94.11 | 1325 / 1296 | contig\_525 | 4767..6083 | AB010293 |
| `FAL11151` | H\_type | fliC | H15 | 98.64 | 1691 / 1689 | contig\_600 | 1739..3429 | AY249999 |
| `FAL11151` | H\_type | fliC | H16 | 85.95 | 1644 / 1575 | contig\_671 | 1725..3354 | JH953794 |
| `FAL11151` | H\_type | fliC | H16 | 92.72 | 1608 / 1575 | contig\_67 | 59876..61476 | JH954529 |
| `FAL11151` | O\_type | wzx | O10 | 91.99 | 1524 / 1521 | contig\_391 | 14369..15883 | AB811599 |
| `FAL11151` | O\_type | wzy | O10 | 93.01 | 1045 / 1044 | contig\_391 | 11310..12350 | AB811599 |
| `FAL11151` | O\_type | wzx | O110 | 98.88 | 1253 / 1251 | contig\_387 | 10029..11273 | AB812049 |
| `FAL11151` | O\_type | wzy | O110 | 99.25 | 1207 / 1206 | contig\_387 | 7710..8908 | AB812049 |
| `FAL11151` | O\_type | wzx | O146 | 98.19 | 1550 / 1539 | contig\_389 | 10929..12469 | DQ465249 |
| `FAL11151` | O\_type | wzy | O146 | 92.56 | 1209 / 1179 | contig\_389 | 7376..8566 | DQ465249 |
| `FAL11151` | O\_type | wzx | O150 | 99.2 | 1503 / 1503 | contig\_404 | 52091..53590 | EU294168 |
| `FAL11151` | O\_type | wzy | O150 | 99.0 | 1099 / 1098 | contig\_404 | 47132..48226 | EU294168 |
| `FAL11151` | O\_type | wzy | O156 | 92.92 | 1257 / 1206 | contig\_476 | 3696..4942 | AB812065 |
| `FAL11151` | O\_type | wzx | O156 | 97.06 | 1157 / 1140 | contig\_476 | 262..1410 | AB812065 |
| `FAL11151` | O\_type | wzt | O162 | 99.2 | 754 / 753 | contig\_477 | 4539..5290 | AB812067 |
| `FAL11151` | O\_type | wzx | O166 | 99.12 | 1254 / 1254 | contig\_384 | 12703..13950 | GU299794 |
| `FAL11151` | O\_type | wzy | O166 | 99.07 | 1074 / 1074 | contig\_384 | 14839..15908 | GU299794 |
| `FAL11151` | O\_type | wzy | O175 | 98.59 | 1133 / 1131 | contig\_323 | 3799..4925 | AB812073 |
| `FAL11151` | O\_type | wzy | O18 | 99.24 | 1191 / 1191 | contig\_392 | 9519..10705 | GU299793 |
| `FAL11151` | O\_type | wzx | O18ac | 99.04 | 1248 / 1248 | contig\_392 | 10762..12004 | GU299793 |
| `FAL11151` | O\_type | wzx | O20 | 99.79 | 1407 / 1410 | contig\_510 | 48086..49489 | KJ778793 |
| `FAL11151` | O\_type | wzy | O20 | 99.25 | 1326 / 1323 | contig\_510 | 50234..51552 | KJ778793 |
| `FAL11151` | O\_type | wzx | O33 | 98.96 | 1245 / 1245 | contig\_383 | 4073..5308 | AB811611 |
| `FAL11151` | O\_type | wzy | O33 | 98.92 | 1204 / 1203 | contig\_383 | 6454..7650 | AB811611 |
| `FAL11151` | O\_type | wzx | O38 | 97.88 | 1319 / 1308 | contig\_385 | 16519..17830 | AB811615 |
| `FAL11151` | O\_type | wzy | O38 | 96.6 | 1236 / 1227 | contig\_385 | 15298..16515 | AB811615 |
| `FAL11151` | O\_type | wzx | O6 | 99.35 | 1239 / 1239 | contig\_395 | 14915..16145 | CP002185 |
| `FAL11151` | O\_type | wzt | O8 | 99.42 | 1215 / 1215 | contig\_402 | 4804..6016 | AB811598 |
| `FAL11151` | O\_type | wzx | O82 | 99.51 | 1230 / 1230 | contig\_388 | 10514..11739 | AB812034 |
| `FAL11151` | O\_type | wzy | O82 | 99.54 | 1083 / 1083 | contig\_388 | 8268..9347 | AB812034 |
| `FAL11151` | O\_type | wzx | O84 | 99.36 | 1398 / 1395 | contig\_474 | 8610..10004 | AB812036 |
| `FAL11151` | O\_type | wzy | O84 | 99.22 | 1157 / 1152 | contig\_474 | 12663..13816 | AB812036 |
| `FAL11151` | O\_type | wzm | O89 | 97.83 | 785 / 780 | contig\_477 | 3748..4532 | AB812038 |
| `FAO96374` | H\_type | fliC | H16 | 94.69 | 1581 / 1575 | contig\_101 | 64094..65668 | JH953794 |
| `FAO96374` | H\_type | fliC | H21 | 94.2 | 1484 / 1476 | contig\_146 | 40..1483 | AIHL01000060 |
| `FAO96374` | H\_type | flkA | H36 | 99.34 | 1673 / 1671 | contig\_459 | 4078..5750 | EF392693 |
| `FAO96374` | H\_type | fliC | H7 | 99.09 | 1758 / 1758 | contig\_1080 | 200..1956 | AF228496 |
| `FAO96374` | O\_type | wzx | O10 | 94.14 | 1535 / 1521 | contig\_70 | 14700..16225 | AB811599 |
| `FAO96374` | O\_type | wzy | O10 | 93.11 | 1045 / 1044 | contig\_70 | 11641..12681 | AB811599 |
| `FAO96374` | O\_type | wzy | O103 | 98.1 | 1155 / 1149 | contig\_74 | 9069..10211 | EF027115 |
| `FAO96374` | O\_type | wzx | O103 | 98.28 | 1277 / 1266 | contig\_74 | 12098..13368 | NC:013353 |
| `FAO96374` | O\_type | wzx | O109 | 94.52 | 1387 / 1365 | contig\_73 | 4141..5511 | HM485572 |
| `FAO96374` | O\_type | wzy | O109 | 94.46 | 1245 / 1221 | contig\_73 | 10959..12188 | HM485572 |
| `FAO96374` | O\_type | wzx | O110 | 97.86 | 1261 / 1251 | contig\_68 | 5413..6662 | AB812049 |
| `FAO96374` | O\_type | wzy | O110 | 98.84 | 1210 / 1206 | contig\_68 | 3088..4288 | AB812049 |
| `FAO96374` | O\_type | wzy | O113 | 99.24 | 1190 / 1188 | contig\_66 | 4985..6168 | AF172324 |
| `FAO96374` | O\_type | wzx | O123 | 96.0 | 1475 / 1446 | contig\_72 | 19260..20723 | DQ676934 |
| `FAO96374` | O\_type | wzx | O128ac | 98.83 | 1447 / 1443 | contig\_52 | 15380..16820 | SAMEA3529399 |
| `FAO96374` | O\_type | wzy | O128ac | 96.87 | 1054 / 1044 | contig\_52 | 11758..12801 | SAMEA3529399 |
| `FAO96374` | O\_type | wzx | O15 | 99.52 | 1245 / 1245 | contig\_67 | 69827..71066 | AY647261 |
| `FAO96374` | O\_type | wzy | O15 | 99.41 | 1179 / 1179 | contig\_67 | 67627..68800 | AY647261 |
| `FAO96374` | O\_type | wzx | O16 | 99.28 | 1251 / 1248 | contig\_71 | 5924..7173 | AB811601 |
| `FAO96374` | O\_type | wzy | O16 | 96.31 | 1192 / 1167 | contig\_71 | 3637..4820 | AB811601 |
| `FAO96374` | O\_type | wzm | O162 | 99.23 | 780 / 780 | contig\_85 | 20117..20894 | AB812067 |
| `FAO96374` | O\_type | wzt | O162 | 99.73 | 754 / 753 | contig\_85 | 19358..20110 | AB812067 |
| `FAO96374` | O\_type | wzx | O166 | 99.04 | 1255 / 1254 | contig\_77 | 11055..12304 | GU299794 |
| `FAO96374` | O\_type | wzy | O166 | 99.16 | 1074 / 1074 | contig\_77 | 9096..10166 | GU299794 |
| `FAO96374` | O\_type | wzx | O17/O77 | 98.23 | 1241 / 1236 | contig\_59 | 11138..12366 | AB812084-DQ000314 |
| `FAO96374` | O\_type | wzy | O18 | 98.74 | 1192 / 1191 | contig\_69 | 15069..16251 | GU299793 |
| `FAO96374` | O\_type | wzx | O185 | 85.6 | 1521 / 1434 | contig\_75 | 2213..3716 | AB812081 |
| `FAO96374` | O\_type | wzy | O185 | 89.21 | 1455 / 1371 | contig\_75 | 4643..6083 | AB812081 |
| `FAO96374` | O\_type | wzx | O186 | 96.0 | 1475 / 1446 | contig\_72 | 19260..20723 | KP710595 |
| `FAO96374` | O\_type | wzy | O186 | 98.35 | 971 / 969 | contig\_72 | 13164..14120 | KP710595 |
| `FAO96374` | O\_type | wzx | O18ac | 98.8 | 1250 / 1248 | contig\_69 | 16308..17550 | GU299793 |
| `FAO96374` | O\_type | wzx | O33 | 99.28 | 1247 / 1245 | contig\_76 | 9728..10971 | AB811611 |
| `FAO96374` | O\_type | wzy | O33 | 99.17 | 1203 / 1203 | contig\_76 | 7387..8584 | AB811611 |
| `FAO96374` | O\_type | wzy | O6 | 99.33 | 1345 / 1344 | contig\_55 | 8175..9511 | CP002185 |
| `FAO96374` | O\_type | wzx | O7 | 99.44 | 1431 / 1431 | contig\_65 | 18717..20143 | CP003034 |
| `FAO96374` | O\_type | wzy | O7 | 99.59 | 726 / 726 | contig\_65 | 14292..15016 | CP003034 |
| `FAO96374` | O\_type | wzy | O73 | 98.28 | 1338 / 1332 | contig\_59 | 9769..11096 | DQ000313 |
| `FAO96374` | O\_type | wzt | O8 | 99.51 | 1215 / 1215 | contig\_1028 | 4968..6181 | AB811598 |
| `FAO96374` | O\_type | wzx | O82 | 99.03 | 1234 / 1230 | contig\_57 | 6784..8014 | AB812034 |
| `FAO96374` | O\_type | wzy | O82 | 98.9 | 1090 / 1083 | contig\_57 | 9182..10268 | AB812034 |
| `FAO96374` | O\_type | wzx | O83 | 97.31 | 1452 / 1443 | contig\_56 | 12943..14385 | AB812035 |
| `FAO96374` | O\_type | wzx | O84 | 98.51 | 1406 / 1395 | contig\_62 | 7535..8934 | AB812036 |
| `FAO96374` | O\_type | wzy | O84 | 99.83 | 1152 / 1152 | contig\_62 | 11595..12745 | AB812036 |
| `FAO96374` | O\_type | wzx | O88 | 99.45 | 1462 / 1458 | contig\_64 | 10613..12071 | AB812037 |
| `FAO96374` | O\_type | wzy | O88 | 98.21 | 1285 / 1275 | contig\_64 | 8416..9691 | AB812037 |
| `FAO96374` | O\_type | wzm | O9 | 96.73 | 794 / 786 | contig\_86 | 8399..9186 | AB010294 |
| `FAO96374` | O\_type | wzt | O9a | 96.55 | 1306 / 1296 | contig\_86 | 7097..8396 | AB010293 |
| `FAO72169` | H\_type | fliC | H16 | 99.37 | 1576 / 1575 | contig\_111 | 22..1590 | JH953794 |
| `FAO72169` | H\_type | fliC | H19 | 99.62 | 1833 / 1833 | contig\_854 | 570..2400 | AY337479 |
| `FAO72169` | H\_type | fliC | H30 | 97.61 | 1714 / 1713 | contig\_112 | 173..1884 | AY250011 |
| `FAO72169` | O\_type | wzy | O1 | 99.61 | 1020 / 1020 | contig\_175 | 9438..10455 | KY115223 |
| `FAO72169` | O\_type | wzx | O1 | 99.06 | 1279 / 1278 | contig\_175 | 12921..14192 | KY115225 |
| `FAO72169` | O\_type | wzy | O1 | 99.61 | 1020 / 1020 | contig\_175 | 9438..10455 | KY115225 |
| `FAO72169` | O\_type | wzy | O106 | 94.33 | 1357 / 1332 | contig\_180 | 1445..2782 | DQ000315 |
| `FAO72169` | O\_type | wzx | O110 | 99.13 | 1258 / 1251 | contig\_181 | 3555..4808 | AB812049 |
| `FAO72169` | O\_type | wzy | O110 | 98.76 | 1211 / 1206 | contig\_181 | 1236..2437 | AB812049 |
| `FAO72169` | O\_type | wzy | O125ab | 97.1 | 1344 / 1323 | contig\_185 | 8400..9733 | SAMEA3529396 |
| `FAO72169` | O\_type | wzy | O139 | 98.95 | 1242 / 1239 | contig\_171 | 13682..14919 | DQ109552 |
| `FAO72169` | O\_type | wzx | O139 | 97.56 | 1232 / 1221 | contig\_171 | 11603..12824 | DQ109552 |
| `FAO72169` | O\_type | wzx | O148 | 98.5 | 1199 / 1191 | contig\_174 | 14456..15647 | AAJT02000037 |
| `FAO72169` | O\_type | wzx | O168 | 99.69 | 1272 / 1272 | contig\_193 | 124..1391 | EU296403 |
| `FAO72169` | O\_type | wzy | O168 | 99.11 | 1236 / 1233 | contig\_193 | 3799..5031 | EU296403 |
| `FAO72169` | O\_type | wzx | O17/O77 | 94.69 | 1261 / 1236 | contig\_180 | 158..1404 | AB812084-DQ000314 |
| `FAO72169` | O\_type | wzx | O170 | 99.12 | 1250 / 1248 | contig\_167 | 102618..103861 | AB812070 |
| `FAO72169` | O\_type | wzy | O170 | 98.64 | 1174 / 1173 | contig\_167 | 98630..99794 | AB812070 |
| `FAO72169` | O\_type | wzy | O174 | 97.98 | 1140 / 1140 | contig\_191 | 2021..3156 | DQ008592 |
| `FAO72169` | O\_type | wzy | O18 | 99.16 | 1191 / 1191 | contig\_173 | 13124..14309 | GU299793 |
| `FAO72169` | O\_type | wzx | O185 | 98.33 | 1434 / 1434 | contig\_179 | 2081..3507 | AB812081 |
| `FAO72169` | O\_type | wzy | O185 | 98.4 | 1371 / 1371 | contig\_179 | 4396..5759 | AB812081 |
| `FAO72169` | O\_type | wzx | O18ac | 99.12 | 1248 / 1248 | contig\_173 | 14366..15609 | GU299793 |
| `FAO72169` | O\_type | wzx | O27 | 98.99 | 1289 / 1287 | contig\_165 | 4914..6197 | GU014555 |
| `FAO72169` | O\_type | wzy | O27 | 99.11 | 1233 / 1233 | contig\_165 | 7967..9189 | GU014555 |
| `FAO72169` | O\_type | wzx | O33 | 99.36 | 1245 / 1245 | contig\_186 | 12378..13618 | AB811611 |
| `FAO72169` | O\_type | wzy | O33 | 99.25 | 1203 / 1203 | contig\_186 | 10036..11234 | AB811611 |
| `FAO72169` | O\_type | wzx | O4 | 99.19 | 1239 / 1239 | contig\_176 | 18583..19818 | AY568960 |
| `FAO72169` | O\_type | wzy | O4 | 98.4 | 1189 / 1188 | contig\_176 | 16504..17688 | AY568960 |
| `FAO72169` | O\_type | wzy | O73 | 94.33 | 1357 / 1332 | contig\_180 | 1445..2782 | DQ000313 |
| `FAO72169` | O\_type | wzx | O75 | 99.09 | 1533 / 1533 | contig\_170 | 6655..8178 | GU299795 |
| `FAO72169` | O\_type | wzy | O75 | 98.98 | 1074 / 1074 | contig\_170 | 13064..14134 | GU299795 |
| `FAO72169` | O\_type | wzx | O79 | 98.63 | 1166 / 1161 | contig\_189 | 2842..4001 | EU294162 |
| `FAO72169` | O\_type | wzy | O79 | 99.09 | 1093 / 1092 | contig\_189 | 970..2057 | EU294162 |
| `FAO72169` | O\_type | wzt | O8 | 99.51 | 1215 / 1215 | contig\_144 | 72504..73717 | AB811598 |
| `FAO72169` | O\_type | wzx | O81 | 98.77 | 1303 / 1302 | contig\_183 | 6273..7561 | CU928162 |
| `FAO72169` | O\_type | wzy | O81 | 98.7 | 1232 / 1230 | contig\_183 | 3110..4330 | CU928162 |
| `FAO72169` | O\_type | wzx | O82 | 99.59 | 1230 / 1230 | contig\_178 | 5354..6580 | AB812034 |
| `FAO72169` | O\_type | wzy | O82 | 99.26 | 1083 / 1083 | contig\_178 | 3109..4187 | AB812034 |
| `FAO72169` | O\_type | wzm | O9 | 96.86 | 797 / 786 | contig\_562 | 6025..6817 | D43637 |
| `FAO72169` | O\_type | wzm | O92 | 96.35 | 821 / 801 | contig\_166 | 12464..13281 | AB812040 |
| `FAO72169` | O\_type | wzt | O92 | 95.6 | 772 / 750 | contig\_166 | 11691..12460 | AB812040 |
| `FAO72169` | O\_type | wzt | O9a | 97.25 | 1307 / 1296 | contig\_562 | 4726..6022 | AB010293 |
| `FAL00958` | H\_type | fliC | H7 | 99.32 | 1758 / 1758 | contig\_277 | 89429..91186 | AF228496 |
| `FAL00958` | O\_type | wzm | O101 | 98.59 | 782 / 780 | contig\_447 | 31055..31836 | CP011061 |
| `FAL00958` | O\_type | wzt | O101 | 99.2 | 754 / 753 | contig\_447 | 30296..31048 | CP011061 |
| `FAL00958` | O\_type | wzx | O103 | 99.84 | 1266 / 1266 | contig\_460 | 29037..30300 | EF027106 |
| `FAL00958` | O\_type | wzy | O103 | 99.65 | 1151 / 1149 | contig\_460 | 26010..27158 | EF027106 |
| `FAL00958` | O\_type | wzy | O106 | 99.32 | 1332 / 1332 | contig\_454 | 23275..24603 | DQ000315 |
| `FAL00958` | O\_type | wzy | O11 | 96.54 | 1244 / 1236 | contig\_463 | 8770..9998 | HQ388393 |
| `FAL00958` | O\_type | wzx | O11 | 94.68 | 1260 / 1236 | contig\_463 | 6802..8044 | HQ388393 |
| `FAL00958` | O\_type | wzx | O113 | 98.39 | 1426 / 1422 | contig\_13 | 16074..17491 | AF172324 |
| `FAL00958` | O\_type | wzy | O113 | 98.24 | 1190 / 1188 | contig\_13 | 13960..15137 | AF172324 |
| `FAL00958` | O\_type | wzy | O117 | 98.2 | 1330 / 1323 | contig\_394 | 120401..121720 | EU694096 |
| `FAL00958` | O\_type | wzx | O13/O129 | 90.73 | 1295 / 1257 | contig\_458 | 14203..15469 | AB972421 |
| `FAL00958` | O\_type | wzx | O13/O129 | 90.73 | 1295 / 1257 | contig\_458 | 14203..15469 | EU296422 |
| `FAL00958` | O\_type | wzy | O13/O129/O135 | 98.02 | 1164 / 1149 | contig\_458 | 11168..12327 | AB972421 |
| `FAL00958` | O\_type | wzy | O13/O135 | 98.02 | 1164 / 1149 | contig\_458 | 11168..12327 | EU296422-EU296423 |
| `FAL00958` | O\_type | wzy | O136 | 98.54 | 1236 / 1233 | contig\_455 | 14340..15565 | AB812059 |
| `FAL00958` | O\_type | wzx | O136 | 98.07 | 1242 / 1239 | contig\_455 | 16655..17877 | AB812059 |
| `FAL00958` | O\_type | wzx | O140 | 98.86 | 1316 / 1314 | contig\_17 | 19997..21305 | AB812060 |
| `FAL00958` | O\_type | wzy | O140 | 99.16 | 1192 / 1191 | contig\_17 | 12158..13341 | AB812060 |
| `FAL00958` | O\_type | wzx | O153 | 98.73 | 1181 / 1179 | contig\_451 | 7681..8849 | KJ755551 |
| `FAL00958` | O\_type | wzy | O153 | 98.89 | 1168 / 1167 | contig\_451 | 9800..10959 | KJ755551 |
| `FAL00958` | O\_type | wzy | O156 | 98.68 | 1208 / 1206 | contig\_468 | 14313..15512 | AB812065 |
| `FAL00958` | O\_type | wzx | O156 | 97.38 | 1146 / 1140 | contig\_468 | 10913..12044 | AB812065 |
| `FAL00958` | O\_type | wzx | O166 | 94.77 | 1261 / 1254 | contig\_19 | 6311..7539 | GU299794 |
| `FAL00958` | O\_type | wzy | O166 | 98.51 | 1076 / 1074 | contig\_19 | 4350..5421 | GU299794 |
| `FAL00958` | O\_type | wzx | O17/O77 | 99.19 | 1236 / 1236 | contig\_454 | 22003..23233 | AB812084-DQ000314 |
| `FAL00958` | O\_type | wzx | O179 | 99.25 | 1464 / 1461 | contig\_470 | 11428..12885 | AB812076 |
| `FAL00958` | O\_type | wzy | O179 | 99.46 | 1104 / 1104 | contig\_470 | 14017..15115 | AB812076 |
| `FAL00958` | O\_type | wzy | O18 | 98.99 | 1191 / 1191 | contig\_456 | 13669..14852 | GU299793 |
| `FAL00958` | O\_type | wzx | O18ac | 99.36 | 1248 / 1248 | contig\_456 | 14909..16155 | GU299793 |
| `FAL00958` | O\_type | wzy | O2 | 95.03 | 966 / 963 | contig\_457 | 13516..14460 | EU549863 |
| `FAL00958` | O\_type | wzy | O2 | 95.03 | 966 / 963 | contig\_457 | 13516..14460 | EU549863 |
| `FAL00958` | O\_type | wzx | O29 | 94.71 | 1456 / 1401 | contig\_439 | 21830..23279 | EU294173 |
| `FAL00958` | O\_type | wzx | O33 | 99.36 | 1245 / 1245 | contig\_453 | 16923..18163 | AB811611 |
| `FAL00958` | O\_type | wzy | O33 | 98.84 | 1206 / 1203 | contig\_453 | 14579..15778 | AB811611 |
| `FAL00958` | O\_type | wzx | O50 | 97.56 | 1271 / 1263 | contig\_457 | 16173..17437 | EU549863-AB811624 |
| `FAL00958` | O\_type | wzx | O61 | 98.86 | 1229 / 1227 | contig\_450 | 7864..9080 | GU220362 |
| `FAL00958` | O\_type | wzy | O61 | 98.18 | 1155 / 1158 | contig\_450 | 5797..6940 | GU220362 |
| `FAL00958` | O\_type | wzx | O7 | 99.09 | 1433 / 1431 | contig\_909 | 15104..16528 | CP003034 |
| `FAL00958` | O\_type | wzy | O7 | 99.72 | 726 / 726 | contig\_909 | 20228..20952 | CP003034 |
| `FAL00958` | O\_type | wzy | O73 | 99.32 | 1332 / 1332 | contig\_454 | 23275..24603 | DQ000313 |
| `FAL00958` | O\_type | wzx | O74 | 97.8 | 1270 / 1260 | contig\_20 | 12934..14194 | AB812030 |
| `FAL00958` | O\_type | wzy | O74 | 98.48 | 1183 / 1176 | contig\_20 | 9978..11153 | AB812030 |
| `FAL00958` | O\_type | wzt | O8 | 99.09 | 1215 / 1215 | contig\_12 | 1421..2634 | AB010150 |
| `FAL00958` | O\_type | wzm | O8 | 99.25 | 795 / 795 | contig\_9 | 12885..13678 | AB010150 |
| `FAL00958` | O\_type | wzt | O8 | 99.51 | 1215 / 1215 | contig\_11 | 7542..8755 | AB811598 |
| `FAL00958` | O\_type | wzy | O8 | 99.2 | 1245 / 1242 | contig\_12 | 16051..17289 | AF013583 |
| `FAL00958` | O\_type | wzx | O81 | 99.0 | 1306 / 1302 | contig\_452 | 12704..14003 | CU928162 |
| `FAL00958` | O\_type | wzy | O81 | 98.46 | 1237 / 1230 | contig\_452 | 9523..10749 | CU928162 |
| `FAL00958` | O\_type | wzx | O84 | 98.86 | 1398 / 1395 | contig\_469 | 12120..13510 | AB812036 |
| `FAL00958` | O\_type | wzy | O84 | 99.05 | 1155 / 1152 | contig\_469 | 16163..17312 | AB812036 |
| `FAL11351` | H\_type | fliC | H8 | 99.46 | 1483 / 1479 | contig\_729 | 40..1521 | AJ884569 |
| `FAL11351` | O\_type | wzy | O106 | 99.55 | 1333 / 1332 | contig\_681 | 6660..7989 | DQ000315 |
| `FAL11351` | O\_type | wzx | O113 | 98.18 | 1432 / 1422 | contig\_680 | 1358..2781 | AF172324 |
| `FAL11351` | O\_type | wzy | O113 | 99.24 | 1191 / 1188 | contig\_680 | 3715..4902 | AF172324 |
| `FAL11351` | O\_type | wzy | O139 | 98.4 | 1247 / 1239 | contig\_1193 | 11227..12469 | DQ109552 |
| `FAL11351` | O\_type | wzx | O139 | 98.94 | 1221 / 1221 | contig\_1193 | 9168..10379 | DQ109552 |
| `FAL11351` | O\_type | wzx | O140 | 98.79 | 1322 / 1314 | contig\_693 | 15129..16446 | AB812060 |
| `FAL11351` | O\_type | wzy | O140 | 98.75 | 1201 / 1191 | contig\_693 | 7270..8466 | AB812060 |
| `FAL11351` | O\_type | wzx | O142 | 98.49 | 1255 / 1245 | contig\_677 | 6185..7436 | AB812061 |
| `FAL11351` | O\_type | wzy | O142 | 98.99 | 1185 / 1182 | contig\_677 | 7488..8664 | AB812061 |
| `FAL11351` | O\_type | wzx | O17/O77 | 99.44 | 1239 / 1236 | contig\_681 | 8033..9268 | AB812084-DQ000314 |
| `FAL11351` | O\_type | wzy | O18 | 99.16 | 1191 / 1191 | contig\_687 | 9134..10321 | GU299793 |
| `FAL11351` | O\_type | wzx | O18ac | 99.12 | 1248 / 1248 | contig\_687 | 10378..11621 | GU299793 |
| `FAL11351` | O\_type | wzx | O20 | 99.57 | 1410 / 1410 | contig\_691 | 16459..17865 | KJ778793 |
| `FAL11351` | O\_type | wzy | O20 | 98.64 | 1327 / 1323 | contig\_691 | 14393..15710 | KJ778793 |
| `FAL11351` | O\_type | wzx | O33 | 99.36 | 1245 / 1245 | contig\_679 | 2719..3959 | AB811611 |
| `FAL11351` | O\_type | wzy | O33 | 99.33 | 1203 / 1203 | contig\_679 | 5104..6303 | AB811611 |
| `FAL11351` | O\_type | wzx | O36 | 87.89 | 1247 / 1245 | contig\_689 | 12608..13851 | AB811613 |
| `FAL11351` | O\_type | wzx | O43 | 99.39 | 1316 / 1314 | contig\_684 | 1207..2516 | AB811619 |
| `FAL11351` | O\_type | wzy | O43 | 99.17 | 1212 / 1212 | contig\_684 | 4845..6047 | AB811619 |
| `FAL11351` | O\_type | wzx | O48 | 99.35 | 1226 / 1224 | contig\_692 | 8750..9971 | AB811622 |
| `FAL11351` | O\_type | wzy | O48 | 98.76 | 1125 / 1122 | contig\_692 | 6370..7486 | AB811622 |
| `FAL11351` | O\_type | wzy | O63 | 96.98 | 1326 / 1311 | contig\_696 | 8477..9795 | EU549862 |
| `FAL11351` | O\_type | wzx | O63 | 98.9 | 1268 / 1263 | contig\_696 | 11301..12563 | EU549862 |
| `FAL11351` | O\_type | wzy | O7 | 99.18 | 728 / 726 | contig\_226 | 9622..10347 | CP003034 |
| `FAL11351` | O\_type | wzy | O73 | 99.55 | 1333 / 1332 | contig\_681 | 6660..7989 | DQ000313 |
| `FAL11351` | O\_type | wzx | O82 | 99.51 | 1231 / 1230 | contig\_682 | 3545..4772 | AB812034 |
| `FAL11351` | O\_type | wzy | O82 | 99.17 | 1083 / 1083 | contig\_682 | 5934..7009 | AB812034 |
| `FAL11351` | O\_type | wzx | O88 | 99.04 | 1458 / 1458 | contig\_1129 | 7285..8735 | AB812037 |
| `FAL11351` | O\_type | wzy | O88 | 99.53 | 1275 / 1275 | contig\_1129 | 9665..10936 | AB812037 |
| `FAL11351` | O\_type | wzm | O9 | 98.6 | 786 / 786 | contig\_917 | 9052..9833 | D43637 |
| `FAL11351` | O\_type | wzt | O9a | 99.38 | 1297 / 1296 | contig\_917 | 7754..9049 | AB010293 |
| `FAL05306` | H\_type | fliC | H16 | 97.04 | 1420 / 1578 | contig\_301 | 123342..124757 | AY337475 |
| `FAL05306` | H\_type | fliC | H16 | 97.04 | 1420 / 1575 | contig\_301 | 123342..124757 | JH953794 |
| `FAL05306` | H\_type | fliC | H16 | 97.04 | 1420 / 1575 | contig\_301 | 123342..124757 | JH954529 |
| `FAL05306` | H\_type | flkA | H36 | 99.82 | 1671 / 1671 | contig\_27 | 4072..5742 | EF392693 |
| `FAL05306` | H\_type | fliC | H7 | 99.72 | 1758 / 1758 | contig\_334 | 176..1933 | AF228496 |
| `FAL05306` | O\_type | wzx | O1 | 99.53 | 1281 / 1278 | contig\_851 | 174..1452 | KY115226 |
| `FAL05306` | O\_type | wzy | O1 | 96.2 | 1054 / 1044 | contig\_851 | 6084..7135 | KY115226 |
| `FAL05306` | O\_type | wzx | O104 | 94.48 | 1322 / 1296 | contig\_790 | 2221..3528 | AF361371 |
| `FAL05306` | O\_type | wzy | O104 | 95.98 | 1120 / 1113 | contig\_790 | 4712..5808 | AF361371 |
| `FAL05306` | O\_type | wzy | O106 | 99.32 | 1332 / 1332 | contig\_305 | 5487..6812 | DQ000315 |
| `FAL05306` | O\_type | wzx | O109 | 96.88 | 1380 / 1365 | contig\_302 | 7868..9230 | HM485572 |
| `FAL05306` | O\_type | wzy | O109 | 98.62 | 1228 / 1221 | contig\_302 | 1330..2551 | HM485572 |
| `FAL05306` | O\_type | wzx | O110 | 99.84 | 1251 / 1251 | contig\_1040 | 3174..4422 | AB812049 |
| `FAL05306` | O\_type | wzy | O110 | 99.5 | 1206 / 1206 | contig\_1040 | 5542..6741 | AB812049 |
| `FAL05306` | O\_type | wzy | O123 | 98.65 | 1257 / 1251 | contig\_303 | 7167..8413 | AB972419 |
| `FAL05306` | O\_type | wzx | O123 | 98.9 | 1448 / 1446 | contig\_303 | 884..2318 | DQ676934 |
| `FAL05306` | O\_type | wzy | O123/O186 | 98.65 | 1257 / 1251 | contig\_303 | 7167..8413 | DQ676934-AB812082 |
| `FAL05306` | O\_type | wzy | O124 | 98.56 | 1112 / 1104 | contig\_791 | 35847..36954 | EU296419 |
| `FAL05306` | O\_type | wzy | O139 | 98.39 | 1245 / 1239 | contig\_786 | 6116..7356 | DQ109552 |
| `FAL05306` | O\_type | wzx | O139 | 96.43 | 1233 / 1221 | contig\_786 | 4043..5259 | DQ109552 |
| `FAL05306` | O\_type | wzx | O140 | 97.73 | 1321 / 1314 | contig\_309 | 5338..6643 | AB812060 |
| `FAL05306` | O\_type | wzx | O147 | 98.39 | 1243 / 1233 | contig\_787 | 4179..5412 | DQ868766 |
| `FAL05306` | O\_type | wzy | O147 | 98.66 | 1193 / 1188 | contig\_787 | 2939..4123 | DQ868766 |
| `FAL05306` | O\_type | wzy | O15 | 99.07 | 1180 / 1179 | contig\_200 | 663..1835 | AY647261 |
| `FAL05306` | O\_type | wzx | O150 | 98.48 | 1509 / 1503 | contig\_784 | 10468..11972 | EU294168 |
| `FAL05306` | O\_type | wzy | O150 | 98.37 | 1104 / 1098 | contig\_784 | 5504..6600 | EU294168 |
| `FAL05306` | O\_type | wzy | O156 | 98.51 | 1209 / 1206 | contig\_971 | 1785..2989 | AB812065 |
| `FAL05306` | O\_type | wzm | O162 | 99.87 | 780 / 780 | contig\_301 | 3732..4510 | AB812067 |
| `FAL05306` | O\_type | wzt | O162 | 99.87 | 753 / 753 | contig\_301 | 4517..5268 | AB812067 |
| `FAL05306` | O\_type | wzx | O164 | 98.83 | 1456 / 1455 | contig\_791 | 41367..42808 | EU296420 |
| `FAL05306` | O\_type | wzy | O164 | 98.56 | 1112 / 1104 | contig\_791 | 35847..36954 | EU296420 |
| `FAL05306` | O\_type | wzx | O166 | 99.04 | 1254 / 1254 | contig\_789 | 7639..8880 | GU299794 |
| `FAL05306` | O\_type | wzy | O166 | 99.26 | 1074 / 1074 | contig\_789 | 5680..6749 | GU299794 |
| `FAL05306` | O\_type | wzx | O17/O77 | 98.79 | 1237 / 1236 | contig\_305 | 6854..8079 | AB812084-DQ000314 |
| `FAL05306` | O\_type | wzy | O186 | 98.65 | 1257 / 1251 | contig\_303 | 7167..8413 | AB812082 |
| `FAL05306` | O\_type | wzx | O186 | 98.9 | 1448 / 1446 | contig\_303 | 884..2318 | KP710595 |
| `FAL05306` | O\_type | wzx | O20 | 99.5 | 1407 / 1410 | scaffold\_316 | 224..1625 | KJ778793 |
| `FAL05306` | O\_type | wzy | O20 | 99.17 | 1323 / 1323 | scaffold\_316 | 2365..3676 | KJ778793 |
| `FAL05306` | O\_type | wzy | O28ac/O42 | 96.13 | 1394 / 1380 | contig\_293 | 8357..9729 | DQ462205-FJ539194 |
| `FAL05306` | O\_type | wzx | O28ac/O42 | 93.68 | 1092 / 1071 | contig\_293 | 6141..7208 | DQ462205-FJ539194 |
| `FAL05306` | O\_type | wzx | O33 | 99.2 | 1245 / 1245 | contig\_287 | 5218..6456 | AB811611 |
| `FAL05306` | O\_type | wzy | O33 | 98.67 | 1204 / 1203 | contig\_287 | 7602..8795 | AB811611 |
| `FAL05306` | O\_type | wzx | O4 | 87.21 | 1345 / 1239 | contig\_788 | 9546..10880 | AY568960 |
| `FAL05306` | O\_type | wzy | O55 | 98.88 | 982 / 978 | contig\_785 | 7898..8873 | CP003109 |
| `FAL05306` | O\_type | wzx | O55 | 98.36 | 1284 / 1278 | contig\_785 | 6606..7880 | JH958641 |
| `FAL05306` | O\_type | wzy | O6 | 99.11 | 1344 / 1344 | contig\_308 | 1319..2651 | CP002185 |
| `FAL05306` | O\_type | wzx | O7 | 99.09 | 1431 / 1431 | contig\_852 | 397..1820 | CP003034 |
| `FAL05306` | O\_type | wzy | O7 | 99.45 | 727 / 726 | contig\_852 | 5516..6241 | CP003034 |
| `FAL05306` | O\_type | wzy | O73 | 99.32 | 1332 / 1332 | contig\_305 | 5487..6812 | DQ000313 |
| `FAL05306` | O\_type | wzt | O8 | 99.26 | 1215 / 1215 | contig\_120 | 5441..6652 | AB811598 |
| `FAL05306` | O\_type | wzx | O81 | 97.11 | 1315 / 1302 | contig\_793 | 6300..7599 | CU928162 |
| `FAL05306` | O\_type | wzy | O81 | 97.18 | 1239 / 1230 | contig\_793 | 3129..4351 | CU928162 |
| `FAL05306` | O\_type | wzx | O88 | 86.68 | 1569 / 1458 | contig\_634 | 1934..3484 | AB812037 |
| `FAL05306` | O\_type | wzy | O88 | 97.45 | 1293 / 1275 | contig\_634 | 4449..5735 | AB812037 |
| `FAQ34034` | H\_type | fliC | H16 | 99.79 | 1422 / 1578 | contig\_501 | 1704..3123 | AY337475 |
| `FAQ34034` | H\_type | fliC | H16 | 99.79 | 1422 / 1575 | contig\_501 | 1704..3123 | JH953794 |
| `FAQ34034` | H\_type | fliC | H16 | 99.79 | 1422 / 1575 | contig\_501 | 1704..3123 | JH954529 |
| `FAQ34034` | H\_type | flkA | H36 | 99.58 | 1672 / 1671 | contig\_361 | 4068..5737 | EF392693 |
| `FAQ34034` | H\_type | fliC | H51 | 97.42 | 1820 / 1818 | contig\_500 | 1712..3530 | AY250027 |
| `FAQ34034` | O\_type | wzx | O10 | 96.72 | 1526 / 1521 | contig\_272 | 8530..10031 | AB811599 |
| `FAQ34034` | O\_type | wzy | O10 | 98.66 | 1045 / 1044 | contig\_272 | 5474..6507 | AB811599 |
| `FAQ34034` | O\_type | wzx | O100 | 91.73 | 1281 / 1242 | contig\_709 | 9686..10948 | AB812045 |
| `FAQ34034` | O\_type | wzy | O100 | 91.18 | 1225 / 1170 | contig\_709 | 12168..13382 | AB812045 |
| `FAQ34034` | O\_type | wzm | O101 | 98.21 | 784 / 780 | contig\_849 | 17669..18448 | CP011061 |
| `FAQ34034` | O\_type | wzt | O101 | 97.48 | 755 / 753 | contig\_849 | 16916..17662 | CP011061 |
| `FAQ34034` | O\_type | wzy | O103 | 98.79 | 1153 / 1149 | contig\_267 | 14763..15909 | EF027115 |
| `FAQ34034` | O\_type | wzx | O103 | 99.37 | 1268 / 1266 | contig\_267 | 17782..19046 | NC:013353 |
| `FAQ34034` | O\_type | wzy | O104 | 97.95 | 1120 / 1113 | contig\_271 | 3433..4546 | AF361371 |
| `FAQ34034` | O\_type | wzx | O104 | 98.83 | 1196 / 1191 | contig\_271 | 933..2120 | AFPS01000083 |
| `FAQ34034` | O\_type | wzx | O128ab | 98.76 | 1446 / 1443 | contig\_255 | 4764..6198 | AY217096 |
| `FAQ34034` | O\_type | wzx | O128ac | 98.76 | 1446 / 1443 | contig\_255 | 4764..6198 | SAMEA3529399 |
| `FAQ34034` | O\_type | wzy | O128ac | 98.66 | 1045 / 1044 | contig\_255 | 1158..2189 | SAMEA3529399 |
| `FAQ34034` | O\_type | wzx | O132 | 95.52 | 1295 / 1284 | contig\_266 | 9432..10700 | AB812056 |
| `FAQ34034` | O\_type | wzy | O132 | 99.25 | 1203 / 1200 | contig\_266 | 4587..5784 | AB812056 |
| `FAQ34034` | O\_type | wzx | O138 | 98.2 | 1279 / 1278 | contig\_264 | 17948..19216 | DQ109551 |
| `FAQ34034` | O\_type | wzy | O138 | 99.01 | 1212 / 1212 | contig\_264 | 11190..12398 | DQ109551 |
| `FAQ34034` | O\_type | wzy | O139 | 99.11 | 1241 / 1239 | contig\_265 | 17607..18844 | DQ109552 |
| `FAQ34034` | O\_type | wzx | O139 | 98.85 | 1221 / 1221 | contig\_265 | 15545..16756 | DQ109552 |
| `FAQ34034` | O\_type | wzx | O15 | 99.68 | 1245 / 1245 | contig\_268 | 18306..19546 | AY647261 |
| `FAQ34034` | O\_type | wzy | O15 | 99.15 | 1179 / 1179 | contig\_268 | 16111..17282 | AY647261 |
| `FAQ34034` | O\_type | wzx | O171 | 98.12 | 1274 / 1266 | contig\_41 | 319947..321209 | AB812071 |
| `FAQ34034` | O\_type | wzy | O171 | 98.02 | 1159 / 1152 | contig\_41 | 322181..323327 | AB812071 |
| `FAQ34034` | O\_type | wzy | O178 | 99.36 | 1098 / 1098 | contig\_890 | 4321..5412 | KJ778799 |
| `FAQ34034` | O\_type | wzy | O18 | 99.08 | 1191 / 1191 | contig\_262 | 32288..33474 | GU299793 |
| `FAQ34034` | O\_type | wzx | O18ac | 98.96 | 1248 / 1248 | contig\_262 | 33531..34773 | GU299793 |
| `FAQ34034` | O\_type | wzx | O20 | 99.36 | 1409 / 1410 | contig\_846 | 16366..17767 | KJ778793 |
| `FAQ34034` | O\_type | wzy | O20 | 99.17 | 1324 / 1323 | contig\_846 | 18510..19823 | KJ778793 |
| `FAQ34034` | O\_type | wzx | O33 | 99.36 | 1245 / 1245 | contig\_254 | 10549..11789 | AB811611 |
| `FAQ34034` | O\_type | wzy | O33 | 98.92 | 1203 / 1203 | contig\_254 | 8211..9405 | AB811611 |
| `FAQ34034` | O\_type | wzx | O36 | 91.1 | 1259 / 1245 | contig\_845 | 3462..4713 | AB811613 |
| `FAQ34034` | O\_type | wzy | O36 | 85.94 | 1259 / 1257 | contig\_845 | 6251..7500 | AB811613 |
| `FAQ34034` | O\_type | wzy | O6 | 99.03 | 1344 / 1344 | contig\_474 | 1305..2642 | AJ426423 |
| `FAQ34034` | O\_type | wzx | O7 | 97.99 | 1442 / 1431 | contig\_477 | 7846..9278 | CP003034 |
| `FAQ34034` | O\_type | wzy | O7 | 98.64 | 734 / 726 | contig\_477 | 3378..4111 | CP003034 |
| `FAQ34034` | O\_type | wzy | O73 | 99.47 | 1332 / 1332 | contig\_260 | 11524..12849 | DQ000313 |
| `FAQ34034` | O\_type | wzx | O73 | 99.26 | 1215 / 1218 | contig\_260 | 10276..11482 | DQ000313 |
| `FAQ34034` | O\_type | wzt | O8 | 99.09 | 1215 / 1215 | contig\_3 | 970293..971507 | AB010150 |
| `FAQ34034` | O\_type | wzt | O8 | 99.51 | 1215 / 1215 | contig\_286 | 85823..87037 | AB811598 |
| `FAQ34034` | O\_type | wzx | O82 | 98.95 | 1235 / 1230 | contig\_259 | 16458..17687 | AB812034 |
| `FAQ34034` | O\_type | wzy | O82 | 99.45 | 1083 / 1083 | contig\_259 | 14209..15287 | AB812034 |
| `FAQ34034` | O\_type | wzx | O83 | 98.61 | 1443 / 1443 | contig\_257 | 16221..17660 | AB812035 |
| `FAQ34034` | O\_type | wzx | O88 | 99.25 | 1459 / 1458 | contig\_479 | 5841..7291 | AB812037 |
| `FAQ34034` | O\_type | wzy | O88 | 99.61 | 1276 / 1275 | contig\_479 | 3644..4915 | AB812037 |
| `FAQ34034` | O\_type | wzm | O89 | 99.62 | 781 / 780 | contig\_40 | 20084..20862 | AB812038 |
| `FAQ34034` | O\_type | wzt | O89 | 99.6 | 753 / 753 | contig\_40 | 19327..20077 | AB812038 |
| `FAQ34034` | O\_type | wzy | O93 | 99.54 | 1512 / 1512 | contig\_261 | 45555..47064 | AB812041 |
| `FAQ34034` | O\_type | wzx | O93 | 99.42 | 1200 / 1200 | contig\_261 | 47086..48279 | AB812041 |
| `FAL16059` | H\_type | fliC | H25 | 85.01 | 1374 / 1332 | contig\_348 | 10235..11578 | AGSG01000116 |
| `FAL16059` | O\_type | wzx | O104 | 99.0 | 1297 / 1296 | contig\_393 | 25412..26699 | AF361371 |
| `FAL16059` | O\_type | wzy | O104 | 99.1 | 1115 / 1113 | contig\_393 | 27894..29003 | AF361371 |
| `FAL16059` | O\_type | wzx | O12 | 99.68 | 1260 / 1260 | contig\_835 | 18135..19391 | AB811600 |
| `FAL16059` | O\_type | wzy | O12 | 99.37 | 1105 / 1104 | contig\_835 | 17023..18122 | AB811600 |
| `FAL16059` | O\_type | wzy | O125ab | 95.13 | 1356 / 1323 | contig\_839 | 19496..20839 | SAMEA3529396 |
| `FAL16059` | O\_type | wzx | O125ab | 98.59 | 1277 / 1272 | contig\_839 | 16477..17743 | SAMEA3529396 |
| `FAL16059` | O\_type | wzx | O128ac | 97.73 | 1452 / 1443 | contig\_830 | 13763..15204 | SAMEA3529399 |
| `FAL16059` | O\_type | wzy | O128ac | 98.76 | 1047 / 1044 | contig\_830 | 10124..11160 | SAMEA3529399 |
| `FAL16059` | O\_type | wzy | O140 | 99.08 | 1193 / 1191 | contig\_833 | 10851..12034 | AB812060 |
| `FAL16059` | O\_type | wzx | O142 | 99.52 | 1245 / 1245 | contig\_827 | 16724..17964 | AB812061 |
| `FAL16059` | O\_type | wzy | O142 | 99.24 | 1183 / 1182 | contig\_827 | 15496..16672 | AB812061 |
| `FAL16059` | O\_type | wzm | O162 | 97.46 | 788 / 780 | contig\_394 | 24475..25254 | AB812067 |
| `FAL16059` | O\_type | wzt | O162 | 97.62 | 757 / 753 | contig\_394 | 23719..24468 | AB812067 |
| `FAL16059` | O\_type | wzx | O166 | 98.73 | 1255 / 1254 | contig\_829 | 17302..18543 | GU299794 |
| `FAL16059` | O\_type | wzy | O166 | 99.26 | 1074 / 1074 | contig\_829 | 15346..16415 | GU299794 |
| `FAL16059` | O\_type | wzx | O23 | 98.76 | 1212 / 1206 | contig\_832 | 14844..16047 | AB811607 |
| `FAL16059` | O\_type | wzy | O23 | 98.83 | 1028 / 1026 | contig\_832 | 16885..17903 | AB811607 |
| `FAL16059` | O\_type | wzx | O26 | 98.97 | 1268 / 1263 | contig\_994 | 208..1468 | AF529080 |
| `FAL16059` | O\_type | wzy | O26 | 98.44 | 1027 / 1023 | contig\_994 | 1534..2553 | AF529080 |
| `FAL16059` | O\_type | wzx | O51 | 97.12 | 1564 / 1560 | contig\_834 | 17219..18769 | AB812020 |
| `FAL16059` | O\_type | wzy | O51 | 97.79 | 1039 / 1038 | contig\_834 | 15318..16349 | AB812020 |
| `FAL16059` | O\_type | wzx | O6 | 99.12 | 1243 / 1239 | contig\_992 | 3359..4595 | CP002185 |
| `FAL16059` | O\_type | wzm | O62 | 99.87 | 768 / 768 | contig\_443 | 44396..45162 | KY379508 |
| `FAL16059` | O\_type | wzx | O7 | 91.02 | 1436 / 1431 | contig\_395 | 17967..19397 | CP003034 |
| `FAL16059` | O\_type | wzy | O7 | 88.51 | 731 / 726 | contig\_395 | 13521..14245 | CP003034 |
| `FAL16059` | O\_type | wzy | O70 | 98.37 | 1290 / 1281 | contig\_993 | 5264..6546 | FN995094 |
| `FAL16059` | O\_type | wzx | O70 | 98.73 | 1258 / 1251 | contig\_993 | 2511..3764 | FN995094 |
| `FAL16059` | O\_type | wzx | O74 | 98.5 | 1267 / 1260 | contig\_831 | 13705..14965 | AB812030 |
| `FAL16059` | O\_type | wzy | O74 | 97.64 | 1185 / 1176 | contig\_831 | 10758..11924 | AB812030 |
| `FAL16059` | O\_type | wzt | O8 | 99.51 | 1215 / 1215 | contig\_393 | 14550..15763 | AB010150 |
| `FAL16059` | O\_type | wzy | O84 | 92.04 | 1169 / 1152 | contig\_1094 | 7123..8265 | AB812036 |
| `FAL16059` | O\_type | wzx | O88 | 99.04 | 1461 / 1458 | contig\_1085 | 5812..7263 | AB812037 |
| `FAL16059` | O\_type | wzy | O88 | 99.14 | 1280 / 1275 | contig\_1085 | 3621..4894 | AB812037 |
| `FAL16059` | O\_type | wzm | O9 | 99.62 | 786 / 786 | contig\_987 | 64..848 | D43637 |
| `FAO71993` | H\_type | fliC | H16 | 99.68 | 1575 / 1575 | contig\_965 | 24..1595 | JH953794 |
| `FAO71993` | H\_type | flkA | H36 | 99.82 | 1671 / 1671 | contig\_1005 | 3406..5076 | EF392693 |
| `FAO71993` | H\_type | fliC | H7 | 98.74 | 1431 / 1758 | contig\_1246 | 49385..50814 | AB334574 |
| `FAO71993` | H\_type | fliC | H7 | 98.74 | 1431 / 1758 | contig\_1246 | 49385..50814 | AF228496 |
| `FAO71993` | O\_type | wzx | O105 | 99.09 | 1432 / 1428 | contig\_558 | 250..1673 | EU294171 |
| `FAO71993` | O\_type | wzy | O105 | 98.16 | 1035 / 1029 | contig\_558 | 5574..6595 | EU294171 |
| `FAO71993` | O\_type | wzy | O106 | 98.95 | 1335 / 1332 | contig\_542 | 1428..2757 | DQ000315 |
| `FAO71993` | O\_type | wzy | O123 | 99.44 | 1251 / 1251 | contig\_539 | 6006..7249 | AB972419 |
| `FAO71993` | O\_type | wzx | O123 | 99.72 | 1446 / 1446 | contig\_539 | 12083..13524 | DQ676934 |
| `FAO71993` | O\_type | wzy | O123/O186 | 99.44 | 1251 / 1251 | contig\_539 | 6006..7249 | DQ676934-AB812082 |
| `FAO71993` | O\_type | wzy | O125ab | 99.4 | 1323 / 1323 | contig\_560 | 6876..8190 | SAMEA3529396 |
| `FAO71993` | O\_type | wzx | O125ab | 98.91 | 1279 / 1272 | contig\_560 | 9951..11225 | SAMEA3529396 |
| `FAO71993` | O\_type | wzx | O132 | 99.84 | 1284 / 1284 | contig\_535 | 7509..8790 | AB812056 |
| `FAO71993` | O\_type | wzy | O132 | 99.42 | 1200 / 1200 | contig\_535 | 2671..3864 | AB812056 |
| `FAO71993` | O\_type | wzx | O134 | 99.39 | 1303 / 1302 | contig\_552 | 3652..4948 | AB812058 |
| `FAO71993` | O\_type | wzy | O134 | 98.86 | 1140 / 1140 | contig\_552 | 1616..2747 | AB812058 |
| `FAO71993` | O\_type | wzx | O142 | 99.76 | 1245 / 1245 | contig\_545 | 5378..6619 | AB812061 |
| `FAO71993` | O\_type | wzy | O142 | 99.49 | 1182 / 1182 | contig\_545 | 4149..5326 | AB812061 |
| `FAO71993` | O\_type | wzx | O150 | 96.0 | 1526 / 1503 | contig\_523 | 10427..11938 | EU294168 |
| `FAO71993` | O\_type | wzy | O150 | 99.27 | 1099 / 1098 | contig\_523 | 5463..6558 | EU294168 |
| `FAO71993` | O\_type | wzy | O156 | 99.34 | 1208 / 1206 | contig\_566 | 3670..4872 | AB812065 |
| `FAO71993` | O\_type | wzx | O156 | 98.95 | 1143 / 1140 | contig\_566 | 261..1395 | AB812065 |
| `FAO71993` | O\_type | wzx | O163 | 98.89 | 1257 / 1254 | contig\_540 | 8478..9725 | AB812068 |
| `FAO71993` | O\_type | wzx | O166 | 98.09 | 1257 / 1254 | contig\_546 | 5812..7051 | GU299794 |
| `FAO71993` | O\_type | wzy | O166 | 98.05 | 1076 / 1074 | contig\_546 | 3841..4911 | GU299794 |
| `FAO71993` | O\_type | wzx | O17/O77 | 98.95 | 1236 / 1236 | contig\_542 | 160..1386 | AB812084-DQ000314 |
| `FAO71993` | O\_type | wzx | O170 | 97.92 | 1250 / 1248 | contig\_544 | 5792..7035 | AB812070 |
| `FAO71993` | O\_type | wzy | O170 | 98.55 | 1176 / 1173 | contig\_544 | 1797..2963 | AB812070 |
| `FAO71993` | O\_type | wzx | O179 | 99.32 | 1463 / 1461 | contig\_567 | 122..1578 | AB812076 |
| `FAO71993` | O\_type | wzy | O179 | 98.74 | 1107 / 1104 | contig\_567 | 2711..3810 | AB812076 |
| `FAO71993` | O\_type | wzx | O181 | 98.98 | 1272 / 1272 | contig\_557 | 1716..2975 | AB812078 |
| `FAO71993` | O\_type | wzy | O181 | 99.35 | 1229 / 1251 | contig\_557 | 2992..4214 | AB812078 |
| `FAO71993` | O\_type | wzy | O186 | 99.44 | 1251 / 1251 | contig\_539 | 6006..7249 | AB812082 |
| `FAO71993` | O\_type | wzx | O186 | 99.72 | 1446 / 1446 | contig\_539 | 12083..13524 | KP710595 |
| `FAO71993` | O\_type | wzx | O22 | 96.59 | 1230 / 1218 | contig\_551 | 1138..2361 | AB811606 |
| `FAO71993` | O\_type | wzy | O22 | 91.92 | 1262 / 1236 | contig\_551 | 4045..5285 | DQ851855 |
| `FAO71993` | O\_type | wzx | O23 | 99.92 | 1206 / 1206 | contig\_547 | 3870..5074 | AB811607 |
| `FAO71993` | O\_type | wzy | O23 | 98.83 | 1026 / 1026 | contig\_547 | 5910..6924 | AB811607 |
| `FAO71993` | O\_type | wzx | O3 | 98.01 | 1507 / 1494 | contig\_537 | 6616..8115 | EU694097 |
| `FAO71993` | O\_type | wzy | O3 | 98.53 | 1090 / 1086 | contig\_537 | 4392..5474 | EU694097 |
| `FAO71993` | O\_type | wzx | O33 | 98.4 | 1248 / 1245 | contig\_554 | 5921..7159 | AB811611 |
| `FAO71993` | O\_type | wzy | O33 | 99.09 | 1203 / 1203 | contig\_554 | 3581..4777 | AB811611 |
| `FAO71993` | O\_type | wzx | O4 | 99.27 | 1239 / 1239 | contig\_1219 | 9282..10518 | AY568960 |
| `FAO71993` | O\_type | wzy | O4 | 98.57 | 1188 / 1188 | contig\_1219 | 7204..8387 | AY568960 |
| `FAO71993` | O\_type | wzy | O46 | 98.86 | 1140 / 1140 | contig\_552 | 1616..2747 | AB811621 |
| `FAO71993` | O\_type | wzx | O51 | 98.01 | 1561 / 1560 | contig\_534 | 7019..8575 | AB812020 |
| `FAO71993` | O\_type | wzy | O51 | 98.17 | 1040 / 1038 | contig\_534 | 5107..6142 | AB812020 |
| `FAO71993` | O\_type | wzm | O52 | 91.05 | 782 / 774 | contig\_1134 | 8664..9443 | AY528413 |
| `FAO71993` | O\_type | wzy | O59 | 95.03 | 1187 / 1170 | contig\_1172 | 2094..3266 | AY654590 |
| `FAO71993` | O\_type | wzy | O6 | 99.4 | 1344 / 1344 | contig\_559 | 6795..8130 | CP002185 |
| `FAO71993` | O\_type | wzx | O7 | 99.65 | 1431 / 1431 | contig\_532 | 10354..11780 | CP003034 |
| `FAO71993` | O\_type | wzy | O7 | 99.59 | 726 / 726 | contig\_532 | 5931..6654 | CP003034 |
| `FAO71993` | O\_type | wzy | O73 | 98.95 | 1335 / 1332 | contig\_542 | 1428..2757 | DQ000313 |
| `FAO71993` | O\_type | wzt | O8 | 99.59 | 1215 / 1215 | contig\_1226 | 827..2041 | AB811598 |
| `FAO71993` | O\_type | wzy | O86 | 98.64 | 1401 / 1401 | contig\_553 | 41128..42512 | AY220982 |
| `FAO71993` | O\_type | wzx | O86 | 99.17 | 1204 / 1203 | contig\_553 | 43137..44332 | AY220982 |
| `FAO71993` | O\_type | wzx | O86 | 99.17 | 1204 / 1203 | contig\_553 | 43137..44332 | AY667408 |
| `FAO71993` | O\_type | wzx | O88 | 95.34 | 1460 / 1458 | contig\_561 | 5850..7305 | AB812037 |
| `FAO71993` | O\_type | wzy | O88 | 93.33 | 1275 / 1275 | contig\_561 | 3656..4926 | AB812037 |
| `FAO71993` | O\_type | wzm | O9 | 99.24 | 786 / 786 | contig\_971 | 25382..26163 | D43637 |
| `FAO71993` | O\_type | wzt | O9a | 99.61 | 1296 / 1296 | contig\_971 | 26166..27460 | AB010293 |
| `FAL11417` | H\_type | fliC | H37 | 99.47 | 1688 / 1686 | contig\_441 | 40945..42632 | AY250017 |
| `FAL11417` | O\_type | wzx | O102 | 98.01 | 1259 / 1251 | contig\_798 | 13629..14878 | AB812047 |
| `FAL11417` | O\_type | wzy | O102 | 98.33 | 1137 / 1131 | contig\_798 | 11492..12619 | AB812047 |
| `FAL11417` | O\_type | wzx | O12 | 99.6 | 1260 / 1260 | contig\_350 | 10231..11487 | AB811600 |
| `FAL11417` | O\_type | wzy | O12 | 99.46 | 1104 / 1104 | contig\_350 | 11500..12598 | AB811600 |
| `FAL11417` | O\_type | wzy | O125ab | 99.17 | 1327 / 1323 | contig\_351 | 10750..12070 | SAMEA3529396 |
| `FAL11417` | O\_type | wzx | O125ab | 99.06 | 1275 / 1272 | contig\_351 | 7738..9004 | SAMEA3529396 |
| `FAL11417` | O\_type | wzy | O134 | 98.77 | 1141 / 1140 | contig\_340 | 3143..4272 | AB812058 |
| `FAL11417` | O\_type | wzy | O139 | 99.19 | 1240 / 1239 | contig\_803 | 12893..14129 | DQ109552 |
| `FAL11417` | O\_type | wzx | O139 | 98.61 | 1222 / 1221 | contig\_803 | 10832..12041 | DQ109552 |
| `FAL11417` | O\_type | wzx | O140 | 97.88 | 1323 / 1314 | contig\_796 | 20171..21480 | AB812060 |
| `FAL11417` | O\_type | wzy | O140 | 99.24 | 1192 / 1191 | contig\_796 | 12352..13535 | AB812060 |
| `FAL11417` | O\_type | wzx | O142 | 99.52 | 1245 / 1245 | contig\_140 | 23321..24559 | AB812061 |
| `FAL11417` | O\_type | wzy | O142 | 99.32 | 1183 / 1182 | contig\_140 | 24611..25787 | AB812061 |
| `FAL11417` | O\_type | wzx | O146 | 99.68 | 1539 / 1539 | contig\_795 | 17163..18696 | DQ465249 |
| `FAL11417` | O\_type | wzy | O146 | 99.41 | 1181 / 1179 | contig\_795 | 13636..14811 | DQ465249 |
| `FAL11417` | O\_type | wzx | O153 | 99.41 | 1180 / 1179 | contig\_339 | 21965..23138 | KJ755551 |
| `FAL11417` | O\_type | wzy | O153 | 99.66 | 1167 / 1167 | contig\_339 | 19848..21010 | KJ755551 |
| `FAL11417` | O\_type | wzx | O154 | 99.15 | 1297 / 1293 | contig\_801 | 11292..12582 | AB812064 |
| `FAL11417` | O\_type | wzy | O154 | 98.1 | 1051 / 1041 | contig\_801 | 7071..8115 | AB812064 |
| `FAL11417` | O\_type | wzx | O16 | 99.28 | 1249 / 1248 | contig\_800 | 15836..17078 | AB811601 |
| `FAL11417` | O\_type | wzy | O16 | 99.14 | 1168 / 1167 | contig\_800 | 13566..14728 | AB811601 |
| `FAL11417` | O\_type | wzm | O162 | 98.21 | 780 / 780 | contig\_335 | 10834..11611 | AB812067 |
| `FAL11417` | O\_type | wzt | O162 | 98.8 | 753 / 753 | contig\_335 | 11618..12367 | AB812067 |
| `FAL11417` | O\_type | wzx | O166 | 94.62 | 1282 / 1254 | contig\_806 | 6329..7588 | GU299794 |
| `FAL11417` | O\_type | wzy | O166 | 98.79 | 1074 / 1074 | contig\_806 | 4375..5440 | GU299794 |
| `FAL11417` | O\_type | wzx | O167 | 96.79 | 1276 / 1266 | contig\_1277 | 113..1374 | EU296408 |
| `FAL11417` | O\_type | wzy | O167 | 96.95 | 1149 / 1251 | contig\_1277 | 5462..6601 | EU296408 |
| `FAL11417` | O\_type | wzy | O18 | 96.41 | 1198 / 1191 | contig\_799 | 9878..11059 | GU299793 |
| `FAL11417` | O\_type | wzx | O18ac | 98.0 | 1252 / 1248 | contig\_799 | 11116..12354 | GU299793 |
| `FAL11417` | O\_type | wzx | O20 | 99.57 | 1411 / 1410 | contig\_805 | 27207..28615 | KJ778793 |
| `FAL11417` | O\_type | wzy | O20 | 98.94 | 1327 / 1323 | contig\_805 | 25146..26464 | KJ778793 |
| `FAL11417` | O\_type | wzx | O21 | 98.95 | 1236 / 1236 | contig\_344 | 8002..9226 | EU694098 |
| `FAL11417` | O\_type | wzy | O21 | 99.17 | 1088 / 1083 | contig\_344 | 11506..12590 | EU694098 |
| `FAL11417` | O\_type | wzx | O22 | 97.7 | 1220 / 1218 | contig\_345 | 11516..12730 | AB811606 |
| `FAL11417` | O\_type | wzy | O22 | 97.49 | 1236 / 1236 | contig\_345 | 8598..9826 | DQ851855 |
| `FAL11417` | O\_type | wzx | O23 | 99.67 | 1206 / 1206 | contig\_343 | 11104..12306 | AB811607 |
| `FAL11417` | O\_type | wzy | O23 | 99.03 | 1028 / 1026 | contig\_343 | 9249..10269 | AB811607 |
| `FAL11417` | O\_type | wzx | O26 | 97.79 | 1266 / 1263 | contig\_1307 | 3199..4455 | AF529080 |
| `FAL11417` | O\_type | wzy | O26 | 98.64 | 1026 / 1023 | contig\_1307 | 2115..3133 | AF529080 |
| `FAL11417` | O\_type | wzx | O43 | 98.63 | 1317 / 1314 | contig\_352 | 6583..7890 | AB811619 |
| `FAL11417` | O\_type | wzy | O43 | 97.87 | 1218 / 1212 | contig\_352 | 10209..11413 | AB811619 |
| `FAL11417` | O\_type | wzy | O46 | 98.77 | 1141 / 1140 | contig\_340 | 3143..4272 | AB811621 |
| `FAL11417` | O\_type | wzx | O46 | 99.21 | 1134 / 1131 | contig\_340 | 948..2076 | AB811621 |
| `FAL11417` | O\_type | wzx | O51 | 97.12 | 1564 / 1560 | contig\_802 | 14864..16413 | AB812020 |
| `FAL11417` | O\_type | wzy | O51 | 96.54 | 1040 / 1038 | contig\_802 | 12966..13987 | AB812020 |
| `FAL11417` | O\_type | wzy | O6 | 99.33 | 1345 / 1344 | contig\_347 | 10695..12031 | CP002185 |
| `FAL11417` | O\_type | wzt | O62 | 99.87 | 741 / 741 | contig\_496 | 43652..44391 | KY379508 |
| `FAL11417` | O\_type | wzx | O7 | 99.3 | 1433 / 1431 | contig\_875 | 15668..17095 | CP003034 |
| `FAL11417` | O\_type | wzy | O7 | 99.59 | 727 / 726 | contig\_875 | 20792..21516 | CP003034 |
| `FAL11417` | O\_type | wzy | O73 | 99.03 | 1340 / 1332 | contig\_341 | 14356..15690 | DQ000313 |
| `FAL11417` | O\_type | wzx | O73 | 98.28 | 1223 / 1218 | contig\_341 | 15732..16942 | DQ000313 |
| `FAL11417` | O\_type | wzt | O8 | 99.67 | 1215 / 1215 | contig\_458 | 4778..5992 | AB811598 |
| `FAL11417` | O\_type | wzx | O82 | 97.74 | 1241 / 1230 | contig\_342 | 7179..8409 | AB812034 |
| `FAL11417` | O\_type | wzy | O82 | 97.16 | 1091 / 1083 | contig\_342 | 9573..10652 | AB812034 |
| `FAL11417` | O\_type | wzy | O86 | 97.37 | 1408 / 1401 | contig\_349 | 19762..21146 | AY220982 |
| `FAL11417` | O\_type | wzx | O86 | 98.02 | 1212 / 1203 | contig\_349 | 17935..19135 | AY220982 |
| `FAL11417` | O\_type | wzx | O86 | 98.02 | 1212 / 1203 | contig\_349 | 17935..19135 | AY667408 |
| `FAL11417` | O\_type | wzx | O88 | 96.86 | 1465 / 1458 | contig\_348 | 15352..16809 | AB812037 |
| `FAL11417` | O\_type | wzy | O88 | 95.47 | 1281 / 1275 | contig\_348 | 17736..19010 | AB812037 |
| `FAL11417` | O\_type | wzm | O9 | 99.62 | 786 / 786 | contig\_457 | 49792..50575 | D43637 |
| `FAL11417` | O\_type | wzt | O9a | 99.61 | 1296 / 1296 | contig\_457 | 50578..51872 | AB010293 |
| `FAQ33890` | H\_type | fliC | H2 | 97.26 | 1499 / 1494 | contig\_68 | 1701..3199 | AIHA01000023 |
| `FAQ33890` | H\_type | fliC | H28 | 98.74 | 1671 / 1740 | contig\_69 | 1714..3382 | AAJT02000052 |
| `FAQ33890` | H\_type | flkA | H3 | 93.17 | 1596 / 1590 | contig\_669 | 4078..5669 | AB128916 |
| `FAQ33890` | O\_type | wzy | O1 | 97.26 | 1021 / 1020 | contig\_33 | 7789..8793 | KY115225 |
| `FAQ33890` | O\_type | wzx | O1 | 95.35 | 1289 / 1278 | contig\_33 | 11273..12559 | KY115226 |
| `FAQ33890` | O\_type | wzx | O102 | 99.44 | 1251 / 1251 | contig\_34 | 24118..25364 | AB812047 |
| `FAQ33890` | O\_type | wzy | O102 | 99.29 | 1131 / 1131 | contig\_34 | 21996..23120 | AB812047 |
| `FAQ33890` | O\_type | wzx | O103 | 93.79 | 1304 / 1266 | contig\_26 | 18749..20038 | EF027106 |
| `FAQ33890` | O\_type | wzy | O103 | 97.67 | 1159 / 1149 | contig\_26 | 15711..16858 | EF027106 |
| `FAQ33890` | O\_type | wzx | O121 | 99.21 | 1398 / 1395 | contig\_18 | 6926..8317 | JN859209 |
| `FAQ33890` | O\_type | wzy | O121 | 99.25 | 1195 / 1191 | contig\_18 | 4284..5474 | JN859216 |
| `FAQ33890` | O\_type | wzy | O123 | 99.36 | 1251 / 1251 | contig\_28 | 16199..17442 | AB972419 |
| `FAQ33890` | O\_type | wzx | O123 | 99.59 | 1446 / 1446 | contig\_28 | 22290..23729 | DQ676934 |
| `FAQ33890` | O\_type | wzy | O123/O186 | 99.36 | 1251 / 1251 | contig\_28 | 16199..17442 | DQ676934-AB812082 |
| `FAQ33890` | O\_type | wzy | O139 | 99.19 | 1239 / 1239 | contig\_39 | 16928..18162 | DQ109552 |
| `FAQ33890` | O\_type | wzx | O139 | 98.77 | 1222 / 1221 | contig\_39 | 14864..16075 | DQ109552 |
| `FAQ33890` | O\_type | wzy | O140 | 99.24 | 1192 / 1191 | contig\_32 | 20456..21639 | AB812060 |
| `FAQ33890` | O\_type | wzx | O140 | 98.76 | 1287 / 1314 | contig\_926 | 2192..3468 | AB812060 |
| `FAQ33890` | O\_type | wzx | O142 | 99.28 | 1250 / 1245 | contig\_46 | 4492..5738 | AB812061 |
| `FAQ33890` | O\_type | wzy | O142 | 98.49 | 1190 / 1182 | contig\_46 | 3256..4440 | AB812061 |
| `FAQ33890` | O\_type | wzx | O15 | 99.52 | 1245 / 1245 | contig\_920 | 25562..26801 | AY647261 |
| `FAQ33890` | O\_type | wzy | O15 | 99.15 | 1179 / 1179 | contig\_920 | 23364..24534 | AY647261 |
| `FAQ33890` | O\_type | wzx | O150 | 99.0 | 1503 / 1503 | contig\_36 | 17524..19021 | EU294168 |
| `FAQ33890` | O\_type | wzy | O150 | 99.36 | 1098 / 1098 | contig\_36 | 12556..13652 | EU294168 |
| `FAQ33890` | O\_type | wzx | O153 | 98.98 | 1181 / 1179 | contig\_19 | 1576..2750 | KJ755551 |
| `FAQ33890` | O\_type | wzy | O153 | 99.57 | 1168 / 1167 | contig\_19 | 3705..4868 | KJ755551 |
| `FAQ33890` | O\_type | wzm | O162 | 99.62 | 780 / 780 | contig\_42 | 153428..154204 | AB812067 |
| `FAQ33890` | O\_type | wzt | O162 | 99.87 | 753 / 753 | contig\_42 | 152670..153421 | AB812067 |
| `FAQ33890` | O\_type | wzx | O163 | 98.57 | 1260 / 1254 | contig\_21 | 8522..9773 | AB812068 |
| `FAQ33890` | O\_type | wzx | O166 | 99.2 | 1255 / 1254 | contig\_23 | 5840..7085 | GU299794 |
| `FAQ33890` | O\_type | wzy | O166 | 99.44 | 1074 / 1074 | contig\_23 | 3881..4952 | GU299794 |
| `FAQ33890` | O\_type | wzx | O17/O77 | 99.03 | 1236 / 1236 | contig\_16 | 158..1384 | AB812084-DQ000314 |
| `FAQ33890` | O\_type | wzx | O171 | 99.45 | 1266 / 1266 | contig\_17 | 4820..6080 | AB812071 |
| `FAQ33890` | O\_type | wzy | O171 | 99.13 | 1153 / 1152 | contig\_17 | 2734..3879 | AB812071 |
| `FAQ33890` | O\_type | wzy | O18 | 98.74 | 1192 / 1191 | contig\_37 | 21844..23029 | GU299793 |
| `FAQ33890` | O\_type | wzy | O186 | 99.36 | 1251 / 1251 | contig\_28 | 16199..17442 | AB812082 |
| `FAQ33890` | O\_type | wzx | O186 | 99.59 | 1446 / 1446 | contig\_28 | 22290..23729 | KP710595 |
| `FAQ33890` | O\_type | wzx | O18ac | 98.8 | 1248 / 1248 | contig\_37 | 23086..24326 | GU299793 |
| `FAQ33890` | O\_type | wzx | O20 | 99.57 | 1409 / 1410 | contig\_1126 | 3573..4977 | KJ778793 |
| `FAQ33890` | O\_type | wzy | O20 | 99.32 | 1324 / 1323 | contig\_1126 | 5719..7034 | KJ778793 |
| `FAQ33890` | O\_type | wzy | O28ac/O42 | 99.13 | 1385 / 1380 | contig\_13 | 7174..8555 | DQ462205-FJ539194 |
| `FAQ33890` | O\_type | wzx | O28ac/O42 | 98.61 | 1076 / 1071 | contig\_13 | 9719..10787 | DQ462205-FJ539194 |
| `FAQ33890` | O\_type | wzx | O33 | 99.36 | 1245 / 1245 | contig\_11 | 14650..15890 | AB811611 |
| `FAQ33890` | O\_type | wzy | O33 | 99.09 | 1203 / 1203 | contig\_11 | 12312..13508 | AB811611 |
| `FAQ33890` | O\_type | wzx | O36 | 87.56 | 1254 / 1245 | contig\_31 | 18128..19376 | AB811613 |
| `FAQ33890` | O\_type | wzx | O4 | 98.95 | 1243 / 1239 | contig\_921 | 26305..27545 | AY568960 |
| `FAQ33890` | O\_type | wzy | O4 | 91.64 | 1232 / 1188 | contig\_921 | 24198..25410 | AY568960 |
| `FAQ33890` | O\_type | wzx | O45 | 99.13 | 1265 / 1263 | contig\_27 | 18131..19391 | CU463050 |
| `FAQ33890` | O\_type | wzy | O45 | 99.73 | 1092 / 1092 | contig\_27 | 16087..17175 | CU463050 |
| `FAQ33890` | O\_type | wzx | O48 | 93.92 | 1250 / 1224 | contig\_35 | 12548..13784 | AB811622 |
| `FAQ33890` | O\_type | wzy | O48 | 98.85 | 1127 / 1122 | contig\_35 | 10167..11287 | AB811622 |
| `FAQ33890` | O\_type | wzy | O5 | 98.34 | 1322 / 1317 | contig\_1125 | 4106..5418 | AB811596 |
| `FAQ33890` | O\_type | wzx | O5 | 98.49 | 1261 / 1257 | contig\_1125 | 2852..4105 | AB811596 |
| `FAQ33890` | O\_type | wzx | O51 | 96.45 | 1579 / 1560 | contig\_45 | 6296..7866 | AB812020 |
| `FAQ33890` | O\_type | wzy | O51 | 97.21 | 1041 / 1038 | contig\_45 | 4391..5423 | AB812020 |
| `FAQ33890` | O\_type | wzx | O6 | 98.87 | 1239 / 1239 | contig\_930 | 90..1319 | CP002185 |
| `FAQ33890` | O\_type | wzx | O7 | 96.81 | 1440 / 1431 | contig\_931 | 3242..4666 | CP003034 |
| `FAQ33890` | O\_type | wzy | O7 | 99.04 | 728 / 726 | contig\_931 | 8361..9085 | CP003034 |
| `FAQ33890` | O\_type | wzy | O73 | 99.55 | 1332 / 1332 | contig\_16 | 1426..2751 | DQ000313 |
| `FAQ33890` | O\_type | wzm | O8 | 99.62 | 795 / 795 | contig\_9 | 3970..4764 | AB010150 |
| `FAQ33890` | O\_type | wzx | O81 | 99.0 | 1302 / 1302 | contig\_22 | 10512..11800 | CU928162 |
| `FAQ33890` | O\_type | wzy | O81 | 96.54 | 1241 / 1230 | contig\_22 | 7342..8564 | CU928162 |
| `FAQ33890` | O\_type | wzx | O82 | 99.59 | 1230 / 1230 | contig\_15 | 5356..6581 | AB812034 |
| `FAQ33890` | O\_type | wzy | O82 | 99.35 | 1083 / 1083 | contig\_15 | 3112..4189 | AB812034 |
| `FAQ33890` | O\_type | wzy | O86 | 98.93 | 1401 / 1401 | contig\_25 | 502331..503718 | AY220982 |
| `FAQ33890` | O\_type | wzx | O86 | 99.33 | 1203 / 1203 | contig\_25 | 504346..505541 | AY220982 |
| `FAQ33890` | O\_type | wzx | O86 | 99.33 | 1203 / 1203 | contig\_25 | 504346..505541 | AY667408 |
| `FAQ33890` | O\_type | wzx | O88 | 99.52 | 1458 / 1458 | contig\_924 | 5881..7333 | AB812037 |
| `FAQ33890` | O\_type | wzy | O88 | 99.53 | 1276 / 1275 | contig\_924 | 8260..9533 | AB812037 |
| `FAQ33890` | O\_type | wzm | O89 | 99.62 | 780 / 780 | contig\_10 | 26066..26842 | AB812038 |
| `FAQ33890` | O\_type | wzt | O89 | 99.73 | 753 / 753 | contig\_10 | 25308..26059 | AB812038 |
| `FAQ33890` | O\_type | wzm | O9 | 99.36 | 787 / 786 | contig\_613 | 137..920 | D43637 |
| `FAL17459` | H\_type | fliC | H12 | 98.49 | 1788 / 1788 | contig\_39 | 137930..139715 | AIFX01000055 |
| `FAL17459` | H\_type | fliC | H8 | 99.53 | 1483 / 1479 | contig\_465 | 1705..3187 | AJ884569 |
| `FAL17459` | O\_type | wzy | O106 | 99.47 | 1332 / 1332 | contig\_12 | 5491..6816 | DQ000315 |
| `FAL17459` | O\_type | wzx | O113 | 97.7 | 1433 / 1422 | contig\_14 | 26..1444 | AF172324 |
| `FAL17459` | O\_type | wzy | O113 | 94.7 | 1226 / 1188 | contig\_14 | 2402..3616 | AF172324 |
| `FAL17459` | O\_type | wzx | O150 | 99.14 | 1504 / 1503 | contig\_28 | 10464..11964 | EU294168 |
| `FAL17459` | O\_type | wzy | O150 | 99.18 | 1098 / 1098 | contig\_28 | 5492..6586 | EU294168 |
| `FAL17459` | O\_type | wzy | O156 | 99.17 | 1207 / 1206 | contig\_18 | 3667..4867 | AB812065 |
| `FAL17459` | O\_type | wzx | O156 | 98.95 | 1142 / 1140 | contig\_18 | 260..1392 | AB812065 |
| `FAL17459` | O\_type | wzy | O17/O44 | 99.47 | 1332 / 1332 | contig\_12 | 5491..6816 | AB812084-AB811620 |
| `FAL17459` | O\_type | wzx | O17/O77 | 99.27 | 1236 / 1236 | contig\_12 | 6858..8084 | AB812084-DQ000314 |
| `FAL17459` | O\_type | wzx | O174 | 96.94 | 1273 / 1272 | contig\_11 | 796..2057 | DQ008592 |
| `FAL17459` | O\_type | wzx | O175 | 99.22 | 1408 / 1407 | contig\_29 | 6594..7997 | AB812073 |
| `FAL17459` | O\_type | wzy | O175 | 98.76 | 1132 / 1131 | contig\_29 | 15025..16149 | AB812073 |
| `FAL17459` | O\_type | wzx | O178 | 99.76 | 1230 / 1230 | contig\_483 | 5558..6784 | KJ778799 |
| `FAL17459` | O\_type | wzy | O18 | 99.16 | 1191 / 1191 | contig\_47 | 1702..2888 | GU299793 |
| `FAL17459` | O\_type | wzx | O18ac | 98.72 | 1250 / 1248 | contig\_47 | 403..1645 | GU299793 |
| `FAL17459` | O\_type | wzx | O21 | 99.11 | 1236 / 1236 | contig\_22 | 10831..12056 | EU694098 |
| `FAL17459` | O\_type | wzy | O21 | 99.54 | 1083 / 1083 | contig\_22 | 7469..8546 | EU694098 |
| `FAL17459` | O\_type | wzx | O22 | 97.21 | 1220 / 1218 | contig\_23 | 7653..8862 | AB811606 |
| `FAL17459` | O\_type | wzy | O22 | 96.39 | 1246 / 1236 | contig\_23 | 10560..11797 | DQ851855 |
| `FAL17459` | O\_type | wzx | O28ab | 96.05 | 1266 / 1260 | contig\_2 | 11557..12817 | AB811608 |
| `FAL17459` | O\_type | wzy | O28ab | 94.69 | 1243 / 1242 | contig\_2 | 13695..14927 | AB811608 |
| `FAL17459` | O\_type | wzx | O29 | 98.79 | 1407 / 1401 | contig\_150 | 18884..20286 | EU294173 |
| `FAL17459` | O\_type | wzx | O6 | 99.52 | 1239 / 1239 | contig\_10 | 88..1321 | CP002185 |
| `FAL17459` | O\_type | wzy | O7 | 99.74 | 1176 / 1176 | contig\_13 | 388755..389927 | AB490074 |
| `FAL17459` | O\_type | wzx | O7 | 99.72 | 1431 / 1431 | contig\_13 | 384079..385505 | CP003034 |
| `FAL17459` | O\_type | wzt | O8 | 99.67 | 1215 / 1215 | contig\_15 | 17264..18477 | AB811598 |
| `FAL17459` | O\_type | wzx | O82 | 99.51 | 1230 / 1230 | contig\_16 | 2224..3448 | AB812034 |
| `FAL17459` | O\_type | wzy | O82 | 99.54 | 1083 / 1083 | contig\_16 | 4616..5695 | AB812034 |
| `FAL17459` | O\_type | wzy | O93 | 97.84 | 1525 / 1512 | contig\_19 | 2043..3556 | AB812041 |
| `FAL17459` | O\_type | wzx | O93 | 99.17 | 1202 / 1200 | contig\_19 | 827..2021 | AB812041 |
| `FAL11387` | H\_type | fliC | H37 | 98.6 | 1504 / 1686 | contig\_668 | 1..1503 | AY250017 |
| `FAL11387` | H\_type | fliC | H8 | 99.86 | 1479 / 1479 | contig\_665 | 1701..3178 | AJ884569 |
| `FAL11387` | O\_type | wzy | O104 | 98.83 | 1115 / 1113 | contig\_628 | 17787..18894 | AF361371 |
| `FAL11387` | O\_type | wzx | O104 | 99.33 | 1191 / 1191 | contig\_628 | 15296..16479 | AFPS01000083 |
| `FAL11387` | O\_type | wzy | O106 | 98.65 | 1332 / 1332 | contig\_602 | 9335..10656 | DQ000315 |
| `FAL11387` | O\_type | wzx | O113 | 99.23 | 1422 / 1422 | contig\_36 | 489172..490587 | AF172324 |
| `FAL11387` | O\_type | wzy | O113 | 99.49 | 1188 / 1188 | contig\_36 | 487054..488237 | AF172324 |
| `FAL11387` | O\_type | wzx | O126 | 97.66 | 1498 / 1485 | contig\_605 | 14198..15683 | DQ465248 |
| `FAL11387` | O\_type | wzy | O126 | 96.5 | 1057 / 1044 | contig\_605 | 4105..5148 | DQ465248 |
| `FAL11387` | O\_type | wzx | O132 | 98.61 | 1293 / 1284 | contig\_624 | 12945..14231 | AB812056 |
| `FAL11387` | O\_type | wzy | O132 | 98.59 | 1207 / 1200 | contig\_624 | 8077..9274 | AB812056 |
| `FAL11387` | O\_type | wzy | O139 | 99.19 | 1239 / 1239 | contig\_616 | 5112..6346 | DQ109552 |
| `FAL11387` | O\_type | wzx | O139 | 98.94 | 1221 / 1221 | contig\_616 | 3047..4258 | DQ109552 |
| `FAL11387` | O\_type | wzx | O140 | 99.09 | 1316 / 1314 | contig\_614 | 25738..27046 | AB812060 |
| `FAL11387` | O\_type | wzy | O140 | 99.58 | 1192 / 1191 | contig\_614 | 17914..19101 | AB812060 |
| `FAL11387` | O\_type | wzx | O141 | 99.28 | 1246 / 1242 | contig\_679 | 282..1522 | DQ868765 |
| `FAL11387` | O\_type | wzy | O141ab | 99.02 | 1117 / 1113 | contig\_679 | 3551..4661 | SAMEA3529400 |
| `FAL11387` | O\_type | wzy | O141ac | 99.02 | 1117 / 1113 | contig\_679 | 3551..4661 | SAMEA3529401 |
| `FAL11387` | O\_type | wzx | O150 | 98.54 | 1504 / 1503 | contig\_680 | 1220..2713 | EU294168 |
| `FAL11387` | O\_type | wzy | O150 | 99.37 | 953 / 1098 | contig\_680 | 6581..7531 | EU294168 |
| `FAL11387` | O\_type | wzx | O154 | 99.46 | 1294 / 1293 | contig\_615 | 12423..13710 | AB812064 |
| `FAL11387` | O\_type | wzy | O154 | 99.52 | 1041 / 1041 | contig\_615 | 8231..9266 | AB812064 |
| `FAL11387` | O\_type | wzx | O163 | 99.28 | 1254 / 1254 | contig\_607 | 7406..8651 | AB812068 |
| `FAL11387` | O\_type | wzx | O166 | 97.78 | 1260 / 1254 | contig\_630 | 17521..18761 | GU299794 |
| `FAL11387` | O\_type | wzy | O166 | 98.98 | 1074 / 1074 | contig\_630 | 15570..16636 | GU299794 |
| `FAL11387` | O\_type | wzx | O17/O77 | 98.79 | 1236 / 1236 | contig\_602 | 10698..11922 | AB812084-DQ000314 |
| `FAL11387` | O\_type | wzx | O170 | 98.72 | 1250 / 1248 | contig\_606 | 4140..5380 | AB812070 |
| `FAL11387` | O\_type | wzy | O170 | 98.98 | 1177 / 1173 | contig\_606 | 8215..9388 | AB812070 |
| `FAL11387` | O\_type | wzx | O179 | 99.73 | 1461 / 1461 | contig\_611 | 4161..5617 | AB812076 |
| `FAL11387` | O\_type | wzy | O179 | 99.73 | 1104 / 1104 | contig\_611 | 6747..7848 | AB812076 |
| `FAL11387` | O\_type | wzy | O18 | 98.99 | 1191 / 1191 | contig\_626 | 73192..74375 | GU299793 |
| `FAL11387` | O\_type | wzx | O18ac | 99.04 | 1248 / 1248 | contig\_626 | 74432..75674 | GU299793 |
| `FAL11387` | O\_type | wzx | O20 | 99.72 | 1407 / 1410 | contig\_678 | 27472..28874 | KJ778793 |
| `FAL11387` | O\_type | wzy | O20 | 99.47 | 1323 / 1323 | contig\_678 | 25412..26727 | KJ778793 |
| `FAL11387` | O\_type | wzx | O22 | 97.38 | 1221 / 1218 | contig\_627 | 8354..9568 | AB811606 |
| `FAL11387` | O\_type | wzy | O22 | 97.09 | 1238 / 1236 | contig\_627 | 11259..12485 | DQ851855 |
| `FAL11387` | O\_type | wzx | O29 | 99.71 | 1401 / 1401 | contig\_899 | 3710..5106 | EU294173 |
| `FAL11387` | O\_type | wzy | O36 | 99.28 | 1257 / 1257 | contig\_625 | 23823..25073 | AB811613 |
| `FAL11387` | O\_type | wzx | O36 | 99.28 | 1245 / 1245 | contig\_625 | 26603..27839 | AB811613 |
| `FAL11387` | O\_type | wzx | O45 | 99.6 | 1264 / 1263 | contig\_610 | 6158..7417 | CU463050 |
| `FAL11387` | O\_type | wzy | O45 | 99.36 | 1092 / 1092 | contig\_610 | 8370..9454 | CU463050 |
| `FAL11387` | O\_type | wzx | O48 | 99.26 | 1224 / 1224 | contig\_617 | 3503..4719 | AB811622 |
| `FAL11387` | O\_type | wzy | O48 | 99.38 | 1122 / 1122 | contig\_617 | 1126..2241 | AB811622 |
| `FAL11387` | O\_type | wzm | O52 | 99.48 | 774 / 774 | contig\_850 | 37196..37967 | AY528413 |
| `FAL11387` | O\_type | wzx | O7 | 99.23 | 1432 / 1431 | contig\_682 | 7662..9088 | CP003034 |
| `FAL11387` | O\_type | wzy | O7 | 99.45 | 727 / 726 | contig\_682 | 12785..13510 | CP003034 |
| `FAL11387` | O\_type | wzy | O73 | 98.65 | 1332 / 1332 | contig\_602 | 9335..10656 | DQ000313 |
| `FAL11387` | O\_type | wzt | O8 | 99.42 | 1215 / 1215 | contig\_609 | 19353..20567 | AB010150 |
| `FAL11387` | O\_type | wzt | O8 | 99.75 | 1215 / 1215 | contig\_603 | 19514..20728 | AB811598 |
| `FAL11387` | O\_type | wzx | O81 | 96.34 | 1313 / 1302 | contig\_629 | 9306..10592 | CU928162 |
| `FAL11387` | O\_type | wzy | O81 | 93.82 | 1246 / 1230 | contig\_629 | 6151..7361 | CU928162 |
| `FAL11387` | O\_type | wzx | O83 | 98.54 | 1443 / 1443 | contig\_608 | 4162..5601 | AB812035 |
| `FAL11387` | O\_type | wzx | O85 | 98.94 | 1225 / 1221 | contig\_604 | 7456..8672 | GU299798 |
| `FAL11387` | O\_type | wzy | O85 | 99.46 | 1119 / 1119 | contig\_604 | 5311..6425 | GU299798 |
| `FAL11387` | O\_type | wzy | O93 | 99.27 | 1514 / 1512 | contig\_613 | 6157..7663 | AB812041 |
| `FAL11387` | O\_type | wzx | O93 | 98.34 | 1206 / 1200 | contig\_613 | 4943..6136 | AB812041 |
| `FAQ33923` | H\_type | fliC | H21 | 98.64 | 1476 / 1476 | contig\_181 | 197983..199457 | AIHL01000060 |
| `FAQ33923` | H\_type | fliC | H8 | 88.83 | 1495 / 1479 | contig\_461 | 1700..3174 | AJ884569 |
| `FAQ33923` | O\_type | wzx | O10 | 99.28 | 1521 / 1521 | contig\_494 | 8517..10027 | AB811599 |
| `FAQ33923` | O\_type | wzy | O10 | 99.52 | 1044 / 1044 | contig\_494 | 5465..6503 | AB811599 |
| `FAQ33923` | O\_type | wzx | O102 | 99.36 | 1251 / 1251 | contig\_181 | 79395..80640 | AB812047 |
| `FAQ33923` | O\_type | wzy | O102 | 99.29 | 1131 / 1131 | contig\_181 | 81639..82763 | AB812047 |
| `FAQ33923` | O\_type | wzy | O104 | 97.68 | 1123 / 1113 | contig\_511 | 3419..4530 | AF361371 |
| `FAQ33923` | O\_type | wzx | O104 | 99.24 | 1191 / 1191 | contig\_511 | 929..2114 | AFPS01000083 |
| `FAQ33923` | O\_type | wzx | O105 | 99.23 | 1428 / 1428 | contig\_495 | 248..1667 | EU294171 |
| `FAQ33923` | O\_type | wzy | O105 | 98.93 | 1029 / 1029 | contig\_495 | 5550..6570 | EU294171 |
| `FAQ33923` | O\_type | wzx | O113 | 97.62 | 1430 / 1422 | contig\_517 | 7064..8477 | AF172324 |
| `FAQ33923` | O\_type | wzy | O113 | 97.99 | 1195 / 1188 | contig\_517 | 4948..6132 | AF172324 |
| `FAQ33923` | O\_type | wzy | O123 | 99.2 | 1251 / 1251 | contig\_503 | 5980..7220 | AB972419 |
| `FAQ33923` | O\_type | wzx | O123 | 99.38 | 1447 / 1446 | contig\_503 | 12055..13493 | DQ676934 |
| `FAQ33923` | O\_type | wzy | O123/O186 | 99.2 | 1251 / 1251 | contig\_503 | 5980..7220 | DQ676934-AB812082 |
| `FAQ33923` | O\_type | wzy | O134 | 98.25 | 1141 / 1140 | contig\_507 | 1621..2747 | AB812058 |
| `FAQ33923` | O\_type | wzy | O139 | 98.71 | 1239 / 1239 | contig\_523 | 408..1636 | DQ109552 |
| `FAQ33923` | O\_type | wzx | O139 | 98.2 | 1222 / 1221 | contig\_523 | 2487..3695 | DQ109552 |
| `FAQ33923` | O\_type | wzy | O140 | 99.16 | 1192 / 1191 | contig\_499 | 2468..3651 | AB812060 |
| `FAQ33923` | O\_type | wzx | O140 | 99.24 | 1314 / 1314 | contig\_525 | 2198..3504 | AB812060 |
| `FAQ33923` | O\_type | wzx | O15 | 98.72 | 1246 / 1245 | contig\_514 | 7165..8396 | AY647261 |
| `FAQ33923` | O\_type | wzy | O15 | 99.24 | 1179 / 1179 | contig\_514 | 4972..6144 | AY647261 |
| `FAQ33923` | O\_type | wzx | O150 | 98.87 | 1503 / 1503 | contig\_488 | 10458..11953 | EU294168 |
| `FAQ33923` | O\_type | wzy | O150 | 99.27 | 1098 / 1098 | contig\_488 | 5500..6594 | EU294168 |
| `FAQ33923` | O\_type | wzm | O162 | 99.87 | 780 / 780 | contig\_473 | 3403..4181 | AB812067 |
| `FAQ33923` | O\_type | wzt | O162 | 99.73 | 753 / 753 | contig\_473 | 4188..4938 | AB812067 |
| `FAQ33923` | O\_type | wzx | O166 | 99.2 | 1254 / 1254 | contig\_508 | 6324..7568 | GU299794 |
| `FAQ33923` | O\_type | wzy | O166 | 99.35 | 1074 / 1074 | contig\_508 | 4368..5438 | GU299794 |
| `FAQ33923` | O\_type | wzy | O18 | 99.08 | 1191 / 1191 | contig\_496 | 4350..5535 | GU299793 |
| `FAQ33923` | O\_type | wzy | O186 | 99.2 | 1251 / 1251 | contig\_503 | 5980..7220 | AB812082 |
| `FAQ33923` | O\_type | wzx | O186 | 99.38 | 1447 / 1446 | contig\_503 | 12055..13493 | KP710595 |
| `FAQ33923` | O\_type | wzx | O18ac | 99.04 | 1248 / 1248 | contig\_496 | 5592..6834 | GU299793 |
| `FAQ33923` | O\_type | wzx | O20 | 99.72 | 1407 / 1410 | contig\_518 | 153..1555 | KJ778793 |
| `FAQ33923` | O\_type | wzy | O20 | 99.24 | 1323 / 1323 | contig\_518 | 2297..3609 | KJ778793 |
| `FAQ33923` | O\_type | wzx | O33 | 99.04 | 1245 / 1245 | contig\_513 | 4887..6123 | AB811611 |
| `FAQ33923` | O\_type | wzy | O33 | 98.84 | 1204 / 1203 | contig\_513 | 2551..3746 | AB811611 |
| `FAQ33923` | O\_type | wzx | O36 | 87.64 | 1262 / 1245 | contig\_519 | 197..1444 | AB811613 |
| `FAQ33923` | O\_type | wzx | O45 | 99.05 | 1264 / 1263 | contig\_506 | 3699..4951 | CU463050 |
| `FAQ33923` | O\_type | wzy | O45 | 98.08 | 1096 / 1092 | contig\_506 | 1660..2744 | CU463050 |
| `FAQ33923` | O\_type | wzx | O46 | 99.38 | 1133 / 1131 | contig\_507 | 3815..4943 | AB811621 |
| `FAQ33923` | O\_type | wzy | O46 | 98.25 | 1141 / 1140 | contig\_507 | 1621..2747 | AB811621 |
| `FAQ33923` | O\_type | wzy | O6 | 99.26 | 1344 / 1344 | contig\_527 | 1307..2641 | AJ426423 |
| `FAQ33923` | O\_type | wzx | O7 | 99.37 | 1432 / 1431 | contig\_521 | 248..1675 | CP003034 |
| `FAQ33923` | O\_type | wzy | O7 | 99.31 | 726 / 726 | contig\_521 | 5370..6092 | CP003034 |
| `FAQ33923` | O\_type | wzy | O71 | 99.15 | 1294 / 1290 | contig\_498 | 1419..2705 | GU445927 |
| `FAQ33923` | O\_type | wzx | O71 | 99.22 | 1276 / 1275 | contig\_498 | 4095..5361 | GU445927 |
| `FAQ33923` | O\_type | wzy | O73 | 99.32 | 1333 / 1332 | contig\_505 | 1409..2733 | DQ000313 |
| `FAQ33923` | O\_type | wzx | O73 | 98.27 | 1217 / 1218 | contig\_505 | 166..1367 | DQ000313 |
| `FAQ33923` | O\_type | wzx | O74 | 94.38 | 1280 / 1260 | contig\_512 | 4272..5520 | AB812030 |
| `FAQ33923` | O\_type | wzy | O74 | 96.89 | 1188 / 1176 | contig\_512 | 1317..2486 | AB812030 |
| `FAQ33923` | O\_type | wzt | O8 | 99.51 | 1215 / 1215 | scaffold\_478 | 47712..48925 | AB811598 |
| `FAQ33923` | O\_type | wzy | O8 | 97.03 | 1244 / 1242 | contig\_510 | 4704..5938 | AF013583 |
| `FAQ33923` | O\_type | wzx | O82 | 99.59 | 1230 / 1230 | contig\_504 | 5348..6573 | AB812034 |
| `FAQ33923` | O\_type | wzy | O82 | 99.45 | 1083 / 1083 | contig\_504 | 3104..4182 | AB812034 |
| `FAQ33923` | O\_type | wzx | O88 | 99.04 | 1458 / 1458 | contig\_866 | 2402..3850 | AB812037 |
| `FAQ33923` | O\_type | wzy | O88 | 99.69 | 1275 / 1275 | contig\_866 | 206..1478 | AB812037 |
| `FAO97359` | H\_type | flkA | H3 | 98.68 | 1596 / 1590 | contig\_174 | 4309..5902 | AB128916 |
| `FAO97359` | H\_type | fliC | H49 | 98.94 | 1697 / 1695 | contig\_407 | 1714..3407 | AY250026 |
| `FAO97359` | H\_type | fliC | H8 | 100.0 | 1479 / 1479 | contig\_408 | 1703..3181 | AJ884569 |
| `FAO97359` | O\_type | wzx | O10 | 91.5 | 1529 / 1521 | contig\_350 | 19486..21001 | AB811599 |
| `FAO97359` | O\_type | wzy | O10 | 92.93 | 1047 / 1044 | contig\_350 | 16430..17470 | AB811599 |
| `FAO97359` | O\_type | wzx | O105 | 99.58 | 1428 / 1428 | contig\_346 | 26504..27926 | EU294171 |
| `FAO97359` | O\_type | wzy | O105 | 99.81 | 1029 / 1029 | contig\_346 | 31813..32839 | EU294171 |
| `FAO97359` | O\_type | wzx | O110 | 99.92 | 1251 / 1251 | contig\_391 | 16708..17957 | AB812049 |
| `FAO97359` | O\_type | wzy | O110 | 99.67 | 1206 / 1206 | contig\_391 | 19078..20279 | AB812049 |
| `FAO97359` | O\_type | wzx | O112ab | 98.57 | 1394 / 1392 | contig\_336 | 12684..14075 | EU296413 |
| `FAO97359` | O\_type | wzy | O112ab | 98.81 | 1179 / 1179 | contig\_336 | 14095..15269 | EU296413 |
| `FAO97359` | O\_type | wzx | O132 | 98.84 | 1291 / 1284 | contig\_74 | 40247..41535 | AB812056 |
| `FAO97359` | O\_type | wzy | O132 | 99.25 | 1201 / 1200 | contig\_74 | 35395..36589 | AB812056 |
| `FAO97359` | O\_type | wzx | O146 | 99.29 | 1540 / 1539 | contig\_314 | 5719..7252 | DQ465249 |
| `FAO97359` | O\_type | wzy | O146 | 98.9 | 1185 / 1179 | contig\_314 | 2185..3362 | DQ465249 |
| `FAO97359` | O\_type | wzx | O147 | 97.58 | 1239 / 1233 | contig\_349 | 14675..15897 | DQ868766 |
| `FAO97359` | O\_type | wzy | O147 | 97.23 | 1193 / 1188 | contig\_349 | 13446..14619 | DQ868766 |
| `FAO97359` | O\_type | wzx | O150 | 98.87 | 1507 / 1503 | contig\_1046 | 12764..14267 | EU294168 |
| `FAO97359` | O\_type | wzy | O150 | 99.45 | 1098 / 1098 | contig\_1046 | 18135..19231 | EU294168 |
| `FAO97359` | O\_type | wzx | O157 | 99.35 | 1394 / 1392 | contig\_356 | 9848..11237 | AB602253 |
| `FAO97359` | O\_type | wzy | O157 | 98.13 | 1176 / 1167 | contig\_356 | 11930..13097 | JH970567 |
| `FAO97359` | O\_type | wzx | O16 | 99.52 | 1250 / 1248 | contig\_1047 | 91..1337 | AB811601 |
| `FAO97359` | O\_type | wzy | O16 | 98.05 | 1178 / 1167 | contig\_1047 | 2444..3613 | AB811601 |
| `FAO97359` | O\_type | wzm | O162 | 99.62 | 780 / 780 | contig\_329 | 25439..26217 | AB812067 |
| `FAO97359` | O\_type | wzt | O162 | 99.73 | 753 / 753 | contig\_329 | 26224..26975 | AB812067 |
| `FAO97359` | O\_type | wzy | O17/O44 | 99.47 | 1332 / 1332 | contig\_335 | 17752..19077 | AB812084-AB811620 |
| `FAO97359` | O\_type | wzx | O17/O77 | 99.51 | 1236 / 1236 | contig\_335 | 19118..20349 | AB812084-DQ000314 |
| `FAO97359` | O\_type | wzx | O170 | 99.12 | 1249 / 1248 | contig\_337 | 23205..24446 | AB812070 |
| `FAO97359` | O\_type | wzy | O170 | 98.89 | 1174 / 1173 | contig\_337 | 27269..28435 | AB812070 |
| `FAO97359` | O\_type | wzy | O18 | 99.41 | 1191 / 1191 | contig\_348 | 14694..15883 | GU299793 |
| `FAO97359` | O\_type | wzx | O18ac | 99.28 | 1248 / 1248 | contig\_348 | 15940..17185 | GU299793 |
| `FAO97359` | O\_type | wzx | O20 | 99.79 | 1407 / 1410 | contig\_353 | 88113..89516 | KJ778793 |
| `FAO97359` | O\_type | wzy | O20 | 99.47 | 1323 / 1323 | contig\_353 | 86055..87370 | KJ778793 |
| `FAO97359` | O\_type | wzx | O28ac | 98.38 | 1236 / 1230 | contig\_328 | 9901..11127 | AB811609 |
| `FAO97359` | O\_type | wzy | O28ac/O42 | 92.82 | 1434 / 1380 | contig\_328 | 12297..13724 | DQ462205-FJ539194 |
| `FAO97359` | O\_type | wzx | O33 | 99.36 | 1245 / 1245 | contig\_312 | 13176..14416 | AB811611 |
| `FAO97359` | O\_type | wzy | O33 | 99.09 | 1203 / 1203 | contig\_312 | 10838..12034 | AB811611 |
| `FAO97359` | O\_type | wzx | O45 | 98.58 | 1266 / 1263 | contig\_354 | 9775..11028 | CU463050 |
| `FAO97359` | O\_type | wzy | O45 | 99.18 | 1094 / 1092 | contig\_354 | 7723..8811 | CU463050 |
| `FAO97359` | O\_type | wzy | O53 | 97.85 | 1303 / 1293 | contig\_352 | 11493..12783 | EU289392 |
| `FAO97359` | O\_type | wzx | O53 | 98.87 | 1235 / 1230 | contig\_352 | 13866..15093 | EU289392 |
| `FAO97359` | O\_type | wzy | O55 | 99.08 | 978 / 978 | contig\_342 | 28743..29711 | CP003109 |
| `FAO97359` | O\_type | wzx | O55 | 99.14 | 1279 / 1278 | contig\_342 | 29729..31001 | JH958641 |
| `FAO97359` | O\_type | wzy | O6 | 99.33 | 1345 / 1344 | contig\_339 | 14662..15998 | CP002185 |
| `FAO97359` | O\_type | wzt | O8 | 99.59 | 1215 / 1215 | contig\_1031 | 853..2067 | AB811598 |
| `FAO97359` | O\_type | wzx | O82 | 99.51 | 1230 / 1230 | contig\_333 | 26929..28153 | AB812034 |
| `FAO97359` | O\_type | wzy | O82 | 99.54 | 1083 / 1083 | contig\_333 | 29321..30400 | AB812034 |
| `FAO97359` | O\_type | wzm | O9 | 98.73 | 789 / 786 | contig\_273 | 109137..109923 | D43637 |
| `FAO97359` | O\_type | wzy | O93 | 99.54 | 1513 / 1512 | contig\_340 | 18088..19594 | AB812041 |
| `FAO97359` | O\_type | wzx | O93 | 99.33 | 1202 / 1200 | contig\_340 | 16869..18066 | AB812041 |
| `FAO97359` | O\_type | wzt | O9a | 99.38 | 1298 / 1296 | contig\_273 | 107841..109134 | AB010293 |
| `FAL01556` | H\_type | fliC | H37 | 99.94 | 1686 / 1686 | contig\_664 | 39060..40745 | AY250017 |
| `FAL01556` | O\_type | wzm | O101 | 99.87 | 780 / 780 | contig\_626 | 9196..9974 | CP011061 |
| `FAL01556` | O\_type | wzt | O101 | 98.81 | 756 / 753 | contig\_626 | 9981..10735 | CP011061 |
| `FAL01556` | O\_type | wzx | O104 | 98.54 | 1303 / 1296 | contig\_620 | 17053..18347 | AF361371 |
| `FAL01556` | O\_type | wzy | O104 | 98.92 | 1113 / 1113 | contig\_620 | 14745..15847 | AF361371 |
| `FAL01556` | O\_type | wzy | O106 | 98.8 | 1338 / 1332 | contig\_613 | 7654..8983 | DQ000315 |
| `FAL01556` | O\_type | wzx | O12 | 99.68 | 1260 / 1260 | contig\_627 | 9279..10535 | AB811600 |
| `FAL01556` | O\_type | wzy | O12 | 99.37 | 1104 / 1104 | contig\_627 | 10548..11645 | AB811600 |
| `FAL01556` | O\_type | wzx | O128ac | 99.51 | 1443 / 1443 | contig\_568 | 8727..10165 | SAMEA3529399 |
| `FAL01556` | O\_type | wzy | O128ac | 98.66 | 1047 / 1044 | contig\_568 | 5120..6157 | SAMEA3529399 |
| `FAL01556` | O\_type | wzx | O13/O129 | 95.92 | 1275 / 1257 | contig\_638 | 6204..7458 | AB972421 |
| `FAL01556` | O\_type | wzx | O13/O129 | 95.92 | 1275 / 1257 | contig\_638 | 6204..7458 | EU296422 |
| `FAL01556` | O\_type | wzy | O13/O129/O135 | 99.22 | 1150 / 1149 | contig\_638 | 3234..4377 | AB972421 |
| `FAL01556` | O\_type | wzy | O13/O135 | 99.22 | 1150 / 1149 | contig\_638 | 3234..4377 | EU296422-EU296423 |
| `FAL01556` | O\_type | wzx | O142 | 97.31 | 1262 / 1245 | contig\_629 | 10710..11960 | AB812061 |
| `FAL01556` | O\_type | wzy | O142 | 96.58 | 1199 / 1182 | contig\_629 | 12012..13199 | AB812061 |
| `FAL01556` | O\_type | wzy | O153 | 98.39 | 1179 / 1167 | contig\_623 | 13365..14541 | KJ755551 |
| `FAL01556` | O\_type | wzx | O153 | 97.48 | 1190 / 1179 | contig\_623 | 15489..16665 | KJ755551 |
| `FAL01556` | O\_type | wzy | O18 | 98.83 | 1194 / 1191 | contig\_633 | 8092..9281 | GU299793 |
| `FAL01556` | O\_type | wzx | O18ac | 97.77 | 1258 / 1248 | contig\_633 | 6784..8035 | GU299793 |
| `FAL01556` | O\_type | wzx | O21 | 99.03 | 1237 / 1236 | contig\_624 | 9731..10958 | EU694098 |
| `FAL01556` | O\_type | wzy | O21 | 99.08 | 1083 / 1083 | contig\_624 | 13234..14306 | EU694098 |
| `FAL01556` | O\_type | wzx | O22 | 97.79 | 1220 / 1218 | contig\_621 | 9611..10826 | AB811606 |
| `FAL01556` | O\_type | wzy | O22 | 97.58 | 1238 / 1236 | contig\_621 | 6685..7918 | DQ851855 |
| `FAL01556` | O\_type | wzx | O23 | 98.28 | 1218 / 1206 | contig\_628 | 10681..11892 | AB811607 |
| `FAL01556` | O\_type | wzy | O23 | 99.22 | 1028 / 1026 | contig\_628 | 8821..9842 | AB811607 |
| `FAL01556` | O\_type | wzy | O29 | 96.88 | 1185 / 1164 | contig\_1073 | 1229..2411 | EU294173 |
| `FAL01556` | O\_type | wzx | O3 | 98.34 | 1510 / 1494 | contig\_625 | 3884..5388 | EU694097 |
| `FAL01556` | O\_type | wzy | O3 | 98.0 | 1098 / 1086 | contig\_625 | 6542..7634 | EU694097 |
| `FAL01556` | O\_type | wzm | O52 | 99.87 | 774 / 774 | contig\_1106 | 39738..40510 | AY528413 |
| `FAL01556` | O\_type | wzy | O6 | 99.04 | 1348 / 1344 | contig\_618 | 9807..11145 | CP002185 |
| `FAL01556` | O\_type | wzx | O6 | 98.31 | 1245 / 1239 | contig\_618 | 8569..9804 | CP002185 |
| `FAL01556` | O\_type | wzt | O62 | 99.87 | 741 / 741 | contig\_555 | 9565..10304 | KY379508 |
| `FAL01556` | O\_type | wzx | O7 | 99.79 | 1431 / 1431 | contig\_631 | 11747..13174 | CP003034 |
| `FAL01556` | O\_type | wzy | O7 | 99.86 | 726 / 726 | contig\_631 | 16876..17600 | CP003034 |
| `FAL01556` | O\_type | wzy | O73 | 98.8 | 1338 / 1332 | contig\_613 | 7654..8983 | DQ000313 |
| `FAL01556` | O\_type | wzx | O73 | 99.18 | 1215 / 1218 | contig\_613 | 9025..10231 | DQ000313 |
| `FAL01556` | O\_type | wzt | O8 | 99.75 | 1215 / 1215 | contig\_912 | 4967..6181 | AB010150 |
| `FAL01556` | O\_type | wzm | O9 | 99.49 | 787 / 786 | contig\_642 | 6609..7393 | D43637 |
| `FAL01556` | O\_type | wzt | O9 | 99.38 | 1297 / 1296 | contig\_911 | 4706..5999 | D43637 |
| `FAL01556` | O\_type | wzy | O93 | 97.4 | 1536 / 1512 | contig\_619 | 3562..5090 | AB812041 |
| `FAL01556` | O\_type | wzx | O93 | 96.05 | 1214 / 1200 | contig\_619 | 2346..3540 | AB812041 |
| `FAL01556` | O\_type | wzt | O99 | 98.78 | 1309 / 1302 | contig\_415 | 7893..9200 | FJ940773 |
| `FAL01556` | O\_type | wzm | O99 | 98.62 | 798 / 795 | contig\_415 | 9202..9995 | FJ940773 |
| `FAL01556` | O\_type | wzt | O9a | 98.46 | 1297 / 1296 | contig\_642 | 7396..8689 | AB010293 |
| `FAL01556` | O\_type | wzm | O9a | 98.47 | 786 / 786 | contig\_911 | 6002..6784 | AB010293 |
| `FAL11384` | H\_type | fliC | H25 | 95.14 | 1337 / 1332 | contig\_512 | 43879..45215 | AGSG01000116 |
| `FAL11384` | O\_type | wzx | O10 | 99.41 | 1522 / 1521 | contig\_72 | 20372..21885 | AB811599 |
| `FAL11384` | O\_type | wzy | O10 | 99.14 | 1045 / 1044 | contig\_72 | 23897..24933 | AB811599 |
| `FAL11384` | O\_type | wzx | O100 | 97.68 | 1249 / 1242 | contig\_112 | 22088..23317 | AB812045 |
| `FAL11384` | O\_type | wzy | O100 | 99.32 | 1176 / 1170 | contig\_112 | 19705..20878 | AB812045 |
| `FAL11384` | O\_type | wzm | O101 | 100.0 | 780 / 780 | contig\_64 | 19396..20175 | CP011061 |
| `FAL11384` | O\_type | wzt | O101 | 99.73 | 753 / 753 | contig\_64 | 20182..20932 | CP011061 |
| `FAL11384` | O\_type | wzx | O102 | 99.04 | 1254 / 1251 | contig\_37 | 6804..8052 | AB812047 |
| `FAL11384` | O\_type | wzy | O102 | 99.29 | 1133 / 1131 | contig\_37 | 9050..10178 | AB812047 |
| `FAL11384` | O\_type | wzx | O110 | 98.97 | 1257 / 1251 | contig\_47 | 18997..20246 | AB812049 |
| `FAL11384` | O\_type | wzy | O110 | 98.68 | 1211 / 1206 | contig\_47 | 21368..22570 | AB812049 |
| `FAL11384` | O\_type | wzy | O114 | 99.1 | 1333 / 1332 | contig\_87 | 17087..18414 | AY573377 |
| `FAL11384` | O\_type | wzx | O114 | 99.2 | 1255 / 1254 | contig\_87 | 19269..20516 | AY573377 |
| `FAL11384` | O\_type | wzx | O12 | 99.76 | 1260 / 1260 | contig\_27 | 16651..17908 | AB811600 |
| `FAL11384` | O\_type | wzy | O12 | 99.64 | 1104 / 1104 | contig\_27 | 17921..19021 | AB811600 |
| `FAL11384` | O\_type | wzy | O123 | 97.62 | 1263 / 1251 | contig\_90 | 6015..7265 | AB972419 |
| `FAL11384` | O\_type | wzx | O123 | 91.77 | 1471 / 1446 | contig\_90 | 12122..13530 | DQ676934 |
| `FAL11384` | O\_type | wzy | O123/O186 | 97.62 | 1263 / 1251 | contig\_90 | 6015..7265 | DQ676934-AB812082 |
| `FAL11384` | O\_type | wzy | O124 | 98.91 | 1104 / 1104 | contig\_42 | 23219..24315 | EU296419 |
| `FAL11384` | O\_type | wzx | O128ac | 98.07 | 1454 / 1443 | contig\_530 | 1855..3299 | SAMEA3529399 |
| `FAL11384` | O\_type | wzy | O128ac | 98.39 | 1054 / 1044 | contig\_530 | 5872..6919 | SAMEA3529399 |
| `FAL11384` | O\_type | wzx | O130 | 98.13 | 1284 / 1275 | contig\_55 | 4416..5686 | EU296421 |
| `FAL11384` | O\_type | wzy | O130 | 97.6 | 1127 / 1116 | contig\_55 | 7666..8784 | EU296421 |
| `FAL11384` | O\_type | wzy | O134 | 97.99 | 1145 / 1140 | contig\_59 | 16167..17296 | AB812058 |
| `FAL11384` | O\_type | wzy | O139 | 98.23 | 1246 / 1239 | contig\_46 | 19696..20935 | DQ109552 |
| `FAL11384` | O\_type | wzx | O139 | 99.02 | 1221 / 1221 | contig\_46 | 21788..23000 | DQ109552 |
| `FAL11384` | O\_type | wzx | O140 | 98.71 | 1320 / 1314 | contig\_40 | 17017..18328 | AB812060 |
| `FAL11384` | O\_type | wzy | O140 | 99.5 | 1192 / 1191 | contig\_40 | 24971..26157 | AB812060 |
| `FAL11384` | O\_type | wzx | O142 | 99.2 | 1248 / 1245 | contig\_76 | 16495..17737 | AB812061 |
| `FAL11384` | O\_type | wzy | O142 | 99.41 | 1185 / 1182 | contig\_76 | 17789..18970 | AB812061 |
| `FAL11384` | O\_type | wzx | O146 | 99.48 | 1541 / 1539 | contig\_48 | 21758..23294 | DQ465249 |
| `FAL11384` | O\_type | wzy | O146 | 99.32 | 1183 / 1179 | contig\_48 | 25645..26823 | DQ465249 |
| `FAL11384` | O\_type | wzx | O148 | 99.16 | 1195 / 1191 | contig\_30 | 19508..20698 | AAJT02000037 |
| `FAL11384` | O\_type | wzx | O150 | 99.27 | 1503 / 1503 | contig\_108 | 21156..22656 | EU294168 |
| `FAL11384` | O\_type | wzy | O150 | 99.45 | 1099 / 1098 | contig\_108 | 16191..17288 | EU294168 |
| `FAL11384` | O\_type | wzx | O153 | 99.32 | 1180 / 1179 | contig\_52 | 22848..24021 | KJ755551 |
| `FAL11384` | O\_type | wzy | O153 | 99.66 | 1167 / 1167 | contig\_52 | 20730..21892 | KJ755551 |
| `FAL11384` | O\_type | wzx | O154 | 99.23 | 1294 / 1293 | contig\_69 | 21118..22402 | AB812064 |
| `FAL11384` | O\_type | wzy | O154 | 98.09 | 1046 / 1041 | contig\_69 | 25564..26600 | AB812064 |
| `FAL11384` | O\_type | wzx | O16 | 98.81 | 1259 / 1248 | contig\_38 | 7873..9128 | AB811601 |
| `FAL11384` | O\_type | wzy | O16 | 99.14 | 1169 / 1167 | contig\_38 | 10239..11401 | AB811601 |
| `FAL11384` | O\_type | wzx | O163 | 99.28 | 1258 / 1254 | contig\_35 | 15060..16313 | AB812068 |
| `FAL11384` | O\_type | wzx | O164 | 99.66 | 1455 / 1455 | contig\_42 | 17365..18817 | EU296420 |
| `FAL11384` | O\_type | wzy | O164 | 98.91 | 1104 / 1104 | contig\_42 | 23219..24315 | EU296420 |
| `FAL11384` | O\_type | wzx | O166 | 99.21 | 1259 / 1254 | contig\_49 | 16866..18119 | GU299794 |
| `FAL11384` | O\_type | wzy | O166 | 98.98 | 1078 / 1074 | contig\_49 | 19012..20086 | GU299794 |
| `FAL11384` | O\_type | wzy | O172 | 97.14 | 1188 / 1176 | contig\_80 | 3414..4593 | AY545992 |
| `FAL11384` | O\_type | wzy | O178 | 99.18 | 1101 / 1098 | contig\_45 | 16922..18016 | KJ778799 |
| `FAL11384` | O\_type | wzy | O18 | 98.58 | 1193 / 1191 | contig\_32 | 20703..21887 | GU299793 |
| `FAL11384` | O\_type | wzy | O186 | 97.62 | 1263 / 1251 | contig\_90 | 6015..7265 | AB812082 |
| `FAL11384` | O\_type | wzx | O186 | 91.77 | 1471 / 1446 | contig\_90 | 12122..13530 | KP710595 |
| `FAL11384` | O\_type | wzx | O18ac | 98.72 | 1250 / 1248 | contig\_32 | 19403..20646 | GU299793 |
| `FAL11384` | O\_type | wzy | O2 | 99.38 | 963 / 963 | contig\_58 | 5000..5956 | EU549863 |
| `FAL11384` | O\_type | wzy | O2 | 99.38 | 963 / 963 | contig\_58 | 5000..5956 | EU549863 |
| `FAL11384` | O\_type | wzx | O21 | 98.95 | 1236 / 1236 | contig\_56 | 9230..10453 | EU694098 |
| `FAL11384` | O\_type | wzy | O21 | 98.99 | 1087 / 1083 | contig\_56 | 12735..13816 | EU694098 |
| `FAL11384` | O\_type | wzx | O22 | 98.03 | 1218 / 1218 | contig\_34 | 22718..23932 | AB811606 |
| `FAL11384` | O\_type | wzy | O22 | 97.73 | 1236 / 1236 | contig\_34 | 19800..21030 | DQ851855 |
| `FAL11384` | O\_type | wzx | O23 | 98.03 | 1218 / 1206 | contig\_75 | 14615..15824 | AB811607 |
| `FAL11384` | O\_type | wzy | O23 | 98.45 | 1030 / 1026 | contig\_75 | 12757..13776 | AB811607 |
| `FAL11384` | O\_type | wzx | O24 | 98.25 | 1198 / 1194 | contig\_29 | 13615..14798 | DQ220292 |
| `FAL11384` | O\_type | wzy | O24 | 98.49 | 1058 / 1053 | contig\_29 | 12552..13599 | DQ220292 |
| `FAL11384` | O\_type | wzx | O29 | 97.67 | 1418 / 1401 | contig\_111 | 27467..28876 | EU294173 |
| `FAL11384` | O\_type | wzx | O3 | 97.43 | 1515 / 1494 | contig\_86 | 15905..17406 | EU694097 |
| `FAL11384` | O\_type | wzy | O3 | 97.73 | 1100 / 1086 | contig\_86 | 13663..14755 | EU694097 |
| `FAL11384` | O\_type | wzy | O36 | 96.32 | 1277 / 1257 | contig\_66 | 8005..9271 | AB811613 |
| `FAL11384` | O\_type | wzx | O36 | 94.06 | 1279 / 1245 | contig\_66 | 5126..6390 | AB811613 |
| `FAL11384` | O\_type | wzx | O43 | 98.18 | 1318 / 1314 | contig\_62 | 9527..10826 | AB811619 |
| `FAL11384` | O\_type | wzy | O43 | 96.97 | 1223 / 1212 | contig\_62 | 13154..14357 | AB811619 |
| `FAL11384` | O\_type | wzy | O46 | 97.99 | 1145 / 1140 | contig\_59 | 16167..17296 | AB811621 |
| `FAL11384` | O\_type | wzx | O46 | 98.07 | 1137 / 1131 | contig\_59 | 13973..15097 | AB811621 |
| `FAL11384` | O\_type | wzx | O50 | 99.6 | 1264 / 1263 | contig\_58 | 2033..3292 | EU549863-AB811624 |
| `FAL11384` | O\_type | wzx | O54 | 99.36 | 1253 / 1251 | contig\_50 | 19733..20979 | AB812085 |
| `FAL11384` | O\_type | wzy | O54 | 99.43 | 1059 / 1059 | contig\_50 | 14853..15905 | AB812085 |
| `FAL11384` | O\_type | wzy | O6 | 98.67 | 1349 / 1344 | contig\_61 | 17627..18963 | CP002185 |
| `FAL11384` | O\_type | wzm | O62 | 99.87 | 768 / 768 | contig\_1113 | 95672..96438 | KY379508 |
| `FAL11384` | O\_type | wzy | O65 | 97.9 | 1287 / 1272 | contig\_88 | 18320..19597 | AB812026 |
| `FAL11384` | O\_type | wzx | O65 | 98.26 | 1267 / 1251 | contig\_88 | 23552..24814 | AB812026 |
| `FAL11384` | O\_type | wzx | O69 | 95.54 | 1526 / 1515 | contig\_43 | 9303..10801 | AB812028 |
| `FAL11384` | O\_type | wzy | O69 | 97.49 | 1155 / 1143 | contig\_43 | 12823..13970 | AB812028 |
| `FAL11384` | O\_type | wzy | O7 | 99.49 | 1177 / 1176 | contig\_60 | 24469..25642 | AB490074 |
| `FAL11384` | O\_type | wzx | O7 | 99.1 | 1438 / 1431 | contig\_60 | 19782..21215 | CP003034 |
| `FAL11384` | O\_type | wzy | O73 | 99.77 | 1332 / 1332 | contig\_31 | 18047..19375 | DQ000313 |
| `FAL11384` | O\_type | wzx | O73 | 99.59 | 1215 / 1218 | contig\_31 | 19417..20626 | DQ000313 |
| `FAL11384` | O\_type | wzt | O8 | 99.59 | 1215 / 1215 | contig\_4 | 78234..79447 | AB811598 |
| `FAL11384` | O\_type | wzx | O80 | 98.86 | 1225 / 1221 | contig\_91 | 16391..17609 | AB812032 |
| `FAL11384` | O\_type | wzy | O80 | 97.41 | 1237 / 1227 | contig\_91 | 11486..12710 | AB812032 |
| `FAL11384` | O\_type | wzx | O84 | 93.78 | 1446 / 1395 | contig\_65 | 8163..9592 | AB812036 |
| `FAL11384` | O\_type | wzy | O84 | 98.71 | 1163 / 1152 | contig\_65 | 12287..13447 | AB812036 |
| `FAL11384` | O\_type | wzy | O86 | 95.79 | 1424 / 1401 | contig\_92 | 6407..7804 | AY220982 |
| `FAL11384` | O\_type | wzx | O86 | 97.78 | 1214 / 1203 | contig\_92 | 8429..9629 | AY220982 |
| `FAL11384` | O\_type | wzx | O86 | 97.78 | 1214 / 1203 | contig\_92 | 8429..9629 | AY667408 |
| `FAL11384` | O\_type | wzx | O88 | 99.38 | 1458 / 1458 | contig\_1449 | 21127..22576 | AB812037 |
| `FAL11384` | O\_type | wzy | O88 | 99.69 | 1275 / 1275 | contig\_1449 | 18936..20206 | AB812037 |
| `FAL11384` | O\_type | wzm | O9 | 99.49 | 787 / 786 | contig\_105 | 20528..21312 | D43637 |
| `FAL11384` | O\_type | wzt | O9a | 99.77 | 1296 / 1296 | contig\_105 | 19231..20525 | AB010293 |
| `FAL11305` | H\_type | fliC | H16 | 95.95 | 1581 / 1575 | contig\_303 | 42..1614 | JH953794 |
| `FAL11305` | H\_type | fliC | H37 | 99.82 | 1686 / 1686 | contig\_302 | 50426..52111 | AY250017 |
| `FAL11305` | O\_type | wzm | O101 | 99.36 | 781 / 780 | contig\_692 | 6358..7134 | CP011061 |
| `FAL11305` | O\_type | wzt | O101 | 99.73 | 754 / 753 | contig\_692 | 7141..7893 | CP011061 |
| `FAL11305` | O\_type | wzx | O12 | 99.45 | 1262 / 1260 | contig\_795 | 6694..7951 | AB811600 |
| `FAL11305` | O\_type | wzy | O12 | 99.55 | 1104 / 1104 | contig\_795 | 7964..9063 | AB811600 |
| `FAL11305` | O\_type | wzx | O140 | 99.07 | 1290 / 1314 | contig\_790 | 2196..3480 | AB812060 |
| `FAL11305` | O\_type | wzx | O142 | 98.56 | 1253 / 1245 | contig\_796 | 11368..12613 | AB812061 |
| `FAL11305` | O\_type | wzy | O142 | 98.74 | 1189 / 1182 | contig\_796 | 12665..13849 | AB812061 |
| `FAL11305` | O\_type | wzx | O146 | 98.08 | 1560 / 1539 | contig\_809 | 5740..7294 | DQ465249 |
| `FAL11305` | O\_type | wzy | O146 | 97.33 | 1197 / 1179 | contig\_809 | 2183..3371 | DQ465249 |
| `FAL11305` | O\_type | wzx | O15 | 97.93 | 1255 / 1245 | contig\_789 | 81..1325 | AY647261 |
| `FAL11305` | O\_type | wzy | O15 | 98.4 | 1191 / 1179 | contig\_789 | 2361..3546 | AY647261 |
| `FAL11305` | O\_type | wzx | O20 | 99.22 | 1413 / 1410 | contig\_808 | 11011..12419 | KJ778793 |
| `FAL11305` | O\_type | wzy | O20 | 96.65 | 1343 / 1323 | contig\_808 | 8935..10262 | KJ778793 |
| `FAL11305` | O\_type | wzx | O23 | 99.42 | 1208 / 1206 | contig\_797 | 7618..8822 | AB811607 |
| `FAL11305` | O\_type | wzy | O23 | 99.22 | 1026 / 1026 | contig\_797 | 5759..6776 | AB811607 |
| `FAL11305` | O\_type | wzx | O29 | 98.65 | 1406 / 1401 | contig\_901 | 19254..20653 | EU294173 |
| `FAL11305` | O\_type | wzy | O3 | 98.26 | 1091 / 1086 | contig\_811 | 4392..5471 | EU694097 |
| `FAL11305` | O\_type | wzx | O43 | 94.25 | 1357 / 1314 | contig\_793 | 7035..8379 | AB811619 |
| `FAL11305` | O\_type | wzy | O43 | 97.39 | 1226 / 1212 | contig\_793 | 10707..11917 | AB811619 |
| `FAL11305` | O\_type | wzm | O52 | 98.47 | 782 / 774 | contig\_902 | 25259..26037 | AY528413 |
| `FAL11305` | O\_type | wzy | O6 | 99.55 | 1344 / 1344 | contig\_792 | 7243..8580 | CP002185 |
| `FAL11305` | O\_type | wzm | O62 | 99.35 | 770 / 768 | contig\_127 | 44417..45184 | KY379508 |
| `FAL11305` | O\_type | wzx | O7 | 99.51 | 1434 / 1431 | contig\_812 | 9304..10734 | CP003034 |
| `FAL11305` | O\_type | wzy | O7 | 99.72 | 726 / 726 | contig\_812 | 4883..5606 | CP003034 |
| `FAL11305` | O\_type | wzt | O8 | 99.59 | 1215 / 1215 | contig\_900 | 4973..6186 | AB811598 |
| `FAL11305` | O\_type | wzx | O88 | 87.28 | 1564 / 1458 | contig\_266 | 158036..159576 | AB812037 |
| `FAL11305` | O\_type | wzy | O88 | 87.68 | 1331 / 1275 | contig\_266 | 155791..157092 | AB812037 |
| `FAL11305` | O\_type | wzt | O9 | 99.08 | 1298 / 1296 | contig\_899 | 4723..6018 | D43637 |
| `FAL11305` | O\_type | wzm | O9 | 98.98 | 787 / 786 | contig\_899 | 6021..6802 | D43637 |
| `FAL01198` | H\_type | fliC | H21 | 92.81 | 1517 / 1476 | contig\_923 | 1708..3219 | AIHL01000060 |
| `FAL01198` | H\_type | flkA | H36 | 97.49 | 1676 / 1671 | contig\_692 | 3449..5120 | EF392693 |
| `FAL01198` | H\_type | fliC | H51 | 88.3 | 1692 / 1818 | contig\_922 | 1724..3412 | AY250027 |
| `FAL01198` | H\_type | fliC | H8 | 96.52 | 1495 / 1479 | contig\_248 | 64238..65732 | AJ884569 |
| `FAL01198` | O\_type | wzx | O10 | 99.41 | 1521 / 1521 | contig\_708 | 8766..10277 | AB811599 |
| `FAL01198` | O\_type | wzy | O10 | 99.52 | 1044 / 1044 | contig\_708 | 5712..6750 | AB811599 |
| `FAL01198` | O\_type | wzm | O101 | 99.87 | 780 / 780 | contig\_1779 | 3393..4171 | CP011061 |
| `FAL01198` | O\_type | wzt | O101 | 99.73 | 753 / 753 | contig\_1779 | 4178..4928 | CP011061 |
| `FAL01198` | O\_type | wzy | O104 | 97.86 | 1121 / 1113 | contig\_716 | 3675..4788 | AF361371 |
| `FAL01198` | O\_type | wzx | O104 | 99.33 | 1193 / 1191 | contig\_716 | 1176..2365 | AFPS01000083 |
| `FAL01198` | O\_type | wzx | O112ac | 98.18 | 1428 / 1422 | contig\_658 | 10811..12223 | EU296405 |
| `FAL01198` | O\_type | wzy | O112ac | 98.54 | 1093 / 1086 | contig\_658 | 6896..7983 | EU296405 |
| `FAL01198` | O\_type | wzx | O113 | 94.33 | 1445 / 1422 | contig\_660 | 137..1558 | AF172324 |
| `FAL01198` | O\_type | wzy | O113 | 96.27 | 1205 / 1188 | contig\_660 | 2526..3715 | AF172324 |
| `FAL01198` | O\_type | wzx | O116 | 97.97 | 1431 / 1428 | contig\_653 | 15596..17019 | AB812051 |
| `FAL01198` | O\_type | wzy | O117 | 99.47 | 1323 / 1323 | contig\_709 | 4887..6202 | EU694096 |
| `FAL01198` | O\_type | wzx | O12 | 99.76 | 1260 / 1260 | contig\_668 | 24375..25632 | AB811600 |
| `FAL01198` | O\_type | wzy | O12 | 99.46 | 1104 / 1104 | contig\_668 | 25645..26743 | AB811600 |
| `FAL01198` | O\_type | wzx | O121 | 99.43 | 1400 / 1395 | contig\_644 | 22335..23731 | JN859209 |
| `FAL01198` | O\_type | wzy | O121 | 99.5 | 1193 / 1191 | contig\_644 | 25182..26370 | JN859209 |
| `FAL01198` | O\_type | wzx | O126 | 99.19 | 1489 / 1485 | contig\_659 | 22972..24454 | DQ465248 |
| `FAL01198` | O\_type | wzy | O126 | 99.04 | 1046 / 1044 | contig\_659 | 12882..13924 | DQ465248 |
| `FAL01198` | O\_type | wzx | O128ac | 99.03 | 1444 / 1443 | contig\_702 | 5359..6797 | SAMEA3529399 |
| `FAL01198` | O\_type | wzy | O128ac | 98.18 | 1044 / 1044 | contig\_702 | 1751..2785 | SAMEA3529399 |
| `FAL01198` | O\_type | wzx | O130 | 98.83 | 1283 / 1275 | contig\_655 | 8779..10056 | EU296421 |
| `FAL01198` | O\_type | wzy | O130 | 99.19 | 1118 / 1116 | contig\_655 | 12025..13140 | EU296421 |
| `FAL01198` | O\_type | wzy | O139 | 98.23 | 1244 / 1239 | contig\_703 | 5097..6333 | DQ109552 |
| `FAL01198` | O\_type | wzx | O139 | 98.86 | 1223 / 1221 | contig\_703 | 3028..4242 | DQ109552 |
| `FAL01198` | O\_type | wzx | O140 | 99.32 | 1314 / 1314 | contig\_710 | 10278..11584 | AB812060 |
| `FAL01198` | O\_type | wzy | O140 | 99.41 | 1193 / 1191 | contig\_710 | 2456..3643 | AB812060 |
| `FAL01198` | O\_type | wzx | O142 | 99.6 | 1246 / 1245 | contig\_669 | 4896..6138 | AB812061 |
| `FAL01198` | O\_type | wzy | O142 | 99.44 | 1062 / 1182 | contig\_669 | 6191..7248 | AB812061 |
| `FAL01198` | O\_type | wzx | O146 | 99.09 | 1543 / 1539 | contig\_714 | 7797..9332 | DQ465249 |
| `FAL01198` | O\_type | wzy | O146 | 99.49 | 1180 / 1179 | contig\_714 | 4272..5446 | DQ465249 |
| `FAL01198` | O\_type | wzx | O148 | 99.66 | 1192 / 1191 | contig\_715 | 4099..5288 | AAJT02000037 |
| `FAL01198` | O\_type | wzx | O150 | 99.27 | 1503 / 1503 | contig\_706 | 10473..11973 | EU294168 |
| `FAL01198` | O\_type | wzy | O150 | 99.36 | 1098 / 1098 | contig\_706 | 5509..6603 | EU294168 |
| `FAL01198` | O\_type | wzx | O153 | 99.32 | 1180 / 1179 | contig\_646 | 14379..15552 | KJ755551 |
| `FAL01198` | O\_type | wzy | O153 | 99.74 | 1168 / 1167 | contig\_646 | 12257..13422 | KJ755551 |
| `FAL01198` | O\_type | wzx | O16 | 98.48 | 1254 / 1248 | contig\_712 | 5877..7123 | AB811601 |
| `FAL01198` | O\_type | wzy | O16 | 99.57 | 1169 / 1167 | contig\_712 | 3603..4768 | AB811601 |
| `FAL01198` | O\_type | wzx | O166 | 99.44 | 1254 / 1254 | contig\_651 | 18881..20127 | GU299794 |
| `FAL01198` | O\_type | wzy | O166 | 99.07 | 1075 / 1074 | contig\_651 | 21016..22085 | GU299794 |
| `FAL01198` | O\_type | wzx | O169 | 99.46 | 1286 / 1284 | contig\_648 | 22809..24089 | AB812069 |
| `FAL01198` | O\_type | wzy | O169 | 99.43 | 1054 / 1053 | contig\_648 | 13600..14648 | AB812069 |
| `FAL01198` | O\_type | wzx | O17/O77 | 99.35 | 1236 / 1236 | contig\_649 | 15823..17053 | AB812084-DQ000314 |
| `FAL01198` | O\_type | wzx | O171 | 95.53 | 1297 / 1266 | contig\_717 | 4839..6121 | AB812071 |
| `FAL01198` | O\_type | wzy | O171 | 98.11 | 1163 / 1152 | contig\_717 | 2738..3892 | AB812071 |
| `FAL01198` | O\_type | wzx | O175 | 99.08 | 1408 / 1407 | contig\_657 | 25573..26977 | AB812073 |
| `FAL01198` | O\_type | wzy | O175 | 98.59 | 1132 / 1131 | contig\_657 | 17421..18542 | AB812073 |
| `FAL01198` | O\_type | wzx | O178 | 99.19 | 1234 / 1230 | contig\_637 | 10109..11337 | KJ778799 |
| `FAL01198` | O\_type | wzy | O18 | 98.66 | 1194 / 1191 | contig\_707 | 4599..5786 | GU299793 |
| `FAL01198` | O\_type | wzx | O18ac | 98.64 | 1250 / 1248 | contig\_707 | 5843..7087 | GU299793 |
| `FAL01198` | O\_type | wzx | O20 | 99.72 | 1408 / 1410 | contig\_1359 | 38922..40326 | KJ778793 |
| `FAL01198` | O\_type | wzy | O20 | 99.47 | 1324 / 1323 | contig\_1359 | 41070..42387 | KJ778793 |
| `FAL01198` | O\_type | wzx | O21 | 99.03 | 1237 / 1236 | contig\_656 | 17038..18264 | EU694098 |
| `FAL01198` | O\_type | wzy | O21 | 99.17 | 1083 / 1083 | contig\_656 | 20549..21624 | EU694098 |
| `FAL01198` | O\_type | wzx | O22 | 98.03 | 1218 / 1218 | contig\_641 | 42053..43267 | AB811606 |
| `FAL01198` | O\_type | wzy | O22 | 97.9 | 1236 / 1236 | contig\_641 | 39131..40363 | DQ851855 |
| `FAL01198` | O\_type | wzx | O23 | 99.75 | 1206 / 1206 | contig\_642 | 12057..13259 | AB811607 |
| `FAL01198` | O\_type | wzy | O23 | 99.42 | 1026 / 1026 | contig\_642 | 10198..11218 | AB811607 |
| `FAL01198` | O\_type | wzy | O28ac/O42 | 99.42 | 1380 / 1380 | contig\_1816 | 2815..4188 | DQ462205-FJ539194 |
| `FAL01198` | O\_type | wzx | O28ac/O42 | 99.63 | 1071 / 1071 | contig\_1816 | 586..1652 | DQ462205-FJ539194 |
| `FAL01198` | O\_type | wzy | O29 | 98.21 | 1174 / 1164 | contig\_1348 | 25363..26527 | EU294173 |
| `FAL01198` | O\_type | wzx | O45 | 97.58 | 1279 / 1263 | contig\_654 | 11912..13183 | CU463050 |
| `FAL01198` | O\_type | wzy | O45 | 99.27 | 1092 / 1092 | contig\_654 | 14140..15225 | CU463050 |
| `FAL01198` | O\_type | wzy | O5 | 98.56 | 1323 / 1317 | contig\_645 | 9725..11042 | AB811596 |
| `FAL01198` | O\_type | wzx | O5 | 97.63 | 1265 / 1257 | contig\_645 | 8471..9724 | AB811596 |
| `FAL01198` | O\_type | wzx | O51 | 97.7 | 1564 / 1560 | contig\_713 | 6289..7844 | AB812020 |
| `FAL01198` | O\_type | wzy | O51 | 97.88 | 1040 / 1038 | contig\_713 | 4388..5421 | AB812020 |
| `FAL01198` | O\_type | wzx | O6 | 99.44 | 1239 / 1239 | contig\_664 | 22965..24197 | CP002185 |
| `FAL01198` | O\_type | wzm | O62 | 100.0 | 768 / 768 | contig\_16 | 44420..45187 | KY379508 |
| `FAL01198` | O\_type | wzx | O7 | 99.65 | 1432 / 1431 | contig\_1357 | 19913..21341 | CP003034 |
| `FAL01198` | O\_type | wzy | O7 | 99.86 | 726 / 726 | contig\_1357 | 25039..25763 | CP003034 |
| `FAL01198` | O\_type | wzy | O73 | 99.62 | 1332 / 1332 | contig\_649 | 14455..15781 | DQ000313 |
| `FAL01198` | O\_type | wzx | O75 | 98.64 | 1542 / 1533 | contig\_721 | 82..1614 | GU299795 |
| `FAL01198` | O\_type | wzy | O75 | 99.07 | 1075 / 1074 | contig\_721 | 6498..7563 | GU299795 |
| `FAL01198` | O\_type | wzt | O8 | 99.75 | 1215 / 1215 | contig\_1819 | 4978..6192 | AB811598 |
| `FAL01198` | O\_type | wzx | O8 | 95.58 | 1198 / 1188 | contig\_638 | 5762..6939 | AF013583 |
| `FAL01198` | O\_type | wzx | O82 | 99.19 | 1233 / 1230 | contig\_643 | 12824..14052 | AB812034 |
| `FAL01198` | O\_type | wzy | O82 | 99.35 | 1083 / 1083 | contig\_643 | 15221..16298 | AB812034 |
| `FAL01198` | O\_type | wzx | O83 | 98.68 | 1443 / 1443 | contig\_635 | 35292..36733 | AB812035 |
| `FAL01198` | O\_type | wzx | O84 | 97.59 | 1408 / 1395 | contig\_666 | 5831..7230 | AB812036 |
| `FAL01198` | O\_type | wzy | O84 | 99.39 | 1155 / 1152 | contig\_666 | 9902..11054 | AB812036 |
| `FAL01198` | O\_type | wzy | O86 | 98.72 | 1403 / 1401 | contig\_647 | 19375..20763 | AY220982 |
| `FAL01198` | O\_type | wzx | O86 | 99.09 | 1205 / 1203 | contig\_647 | 17550..18747 | AY220982 |
| `FAL01198` | O\_type | wzx | O86 | 99.09 | 1205 / 1203 | contig\_647 | 17550..18747 | AY667408 |
| `FAL01198` | O\_type | wzx | O88 | 99.11 | 1459 / 1458 | contig\_665 | 24336..25787 | AB812037 |
| `FAL01198` | O\_type | wzy | O88 | 99.53 | 1275 / 1275 | contig\_665 | 26714..27985 | AB812037 |
| `FAL01198` | O\_type | wzt | O9 | 96.99 | 1296 / 1296 | contig\_636 | 39695..40989 | D43637 |
| `FAL01198` | O\_type | wzm | O9 | 99.75 | 787 / 786 | contig\_636 | 38906..39692 | D43637 |
| `FAL01198` | O\_type | wzt | O9a | 99.92 | 1296 / 1296 | contig\_1820 | 4719..6014 | AB010293 |

---

## ABRICATE\_NCBIAMRPLUS

The results table shown here is a collection from all samples.

| #FILE | NUM\_FOUND | LHR\_hdeD | LHR\_hsp20A | LHR\_hsp20B | LHR\_kefB | LHR\_psiE | LHR\_trx | LHR\_yfdX1 | LHR\_yfdX2 | SMR\_efflux\_emrE | aac(2')-IIa | aac(3)-VIa | aadA1 | aadA2 | aadA25 | aadA5 | acrF | air | alpha | alpha-5 | aph(3'')-Ib | aph(3')-IIa | aph(3')-Ia | aph(6)-Id | ariR | arsA | arsB\_pKW301 | arsC\_gluta | arsD | arsR\_K-12 | arsR\_R46 | arsR\_pKW301 | asr | astA | beta-1 | blaCTX-M-15 | blaCTX-M-55 | blaEC-15 | blaEC-18 | blaEC-5 | blaSHV-1 | blaTEM-1 | blaTEM-122 | blaTEM-141 | blaTEM-150 | blaTEM-156 | bmaE | capU | catA1 | cdtB\_I | cdtB\_III | cif | cmlA1 | cmlA4 | cnf1 | cnf2 | cvaC | dfrA1 | dfrA12 | dfrA14 | dfrA17 | dfrA5 | ehxA | eilA | emrD | epeA | epsilon | epsilon-3 | erm(B) | espA | espB | espC | espF | espI | espJ | espP | espX1 | etpD | f17a | f17g | fdeC | fieF | floR | fosA7.5 | gamma-2 | hlyA-alpha | ibeA | iha | ireA | iroB | iroC | iroD | iroE | iroN | iss | iucA | iucB | iucC | iucD | iutA | katP | lpfA-O113 | lpfA1 | lpfA2 | lpfA\_gen | mchB | mchF | mdtM | merA | merC | merD | merE | merR\_Ps | merT | mph(A) | mph(B) | nleA | nleB | nleB2 | nleC | papC | papE | papF | papG-II | papH | pcoA | pcoB | pcoC | pcoD | pcoE | pcoR | pcoS | pic\_auto | qacEdelta1 | qacG2 | qacL | qnrB19 | qnrS1 | qnrS2 | qnrS4 | saa | sat2\_fam | sepA | sfaF | sfaS | silA | silB | silC | silE | silF | silP | silR | silS | sslE | sta1 | stxA1a | stxA2d | stxB1a | stxB2a | stxB2c | stxB2e | sul1 | sul2 | sul3 | terW | terZ | tet(A) | tet(B) | tet(C) | tet(M) | tir | tsh | vac\_auto | ybtP | ybtQ |
| --- | --- | --- | --- | --- | --- | --- | --- | --- | --- | --- | --- | --- | --- | --- | --- | --- | --- | --- | --- | --- | --- | --- | --- | --- | --- | --- | --- | --- | --- | --- | --- | --- | --- | --- | --- | --- | --- | --- | --- | --- | --- | --- | --- | --- | --- | --- | --- | --- | --- | --- | --- | --- | --- | --- | --- | --- | --- | --- | --- | --- | --- | --- | --- | --- | --- | --- | --- | --- | --- | --- | --- | --- | --- | --- | --- | --- | --- | --- | --- | --- | --- | --- | --- | --- | --- | --- | --- | --- | --- | --- | --- | --- | --- | --- | --- | --- | --- | --- | --- | --- | --- | --- | --- | --- | --- | --- | --- | --- | --- | --- | --- | --- | --- | --- | --- | --- | --- | --- | --- | --- | --- | --- | --- | --- | --- | --- | --- | --- | --- | --- | --- | --- | --- | --- | --- | --- | --- | --- | --- | --- | --- | --- | --- | --- | --- | --- | --- | --- | --- | --- | --- | --- | --- | --- | --- | --- | --- | --- | --- | --- | --- | --- | --- | --- | --- | --- | --- | --- | --- | --- | --- | --- | --- | --- | --- |
| `FAL00958` | 45 | . | . | . | . | . | . | . | . | 100.00;99.70;100.00 | . | . | . | . | . | . | 99.87 | . | . | . | . | . | . | 99.88 | 100.00;100.00 | . | . | . | . | 100.00 | . | . | 100.00 | 100.00;100.00;100.00;100.00 | . | . | . | . | 100.00 | . | . | . | . | . | . | . | . | . | . | . | . | 99.29 | . | . | 99.70 | . | 99.04 | . | . | 99.16 | . | . | . | 99.65 | 100.00 | . | . | . | . | 99.65 | 99.28 | . | . | . | 99.08 | . | 99.51 | . | . | . | 99.39 | 100.00 | . | . | 99.68 | 99.54 | 99.78 | . | 99.12 | 99.55;99.73;99.55;99.55 | 98.52;99.70;99.89;99.84 | 99.59;99.51;99.59;99.76 | 99.58;99.90;99.90;100.00 | 99.68;99.86;99.82;99.82 | 100.00;100.00;99.71;99.71;100.00 | 98.21 | . | . | . | . | . | 100.00;100.00 | . | . | 100.00 | 100.00 | 99.52;99.76 | 99.76 | . | . | . | . | . | . | . | . | . | 99.49 | 99.39 | . | . | . | . | . | . | . | . | . | . | . | . | . | 99.66;99.51;99.68;84.84 | . | . | . | . | . | . | . | . | . | . | 99.35 | 99.80 | . | . | . | . | . | . | . | . | . | . | . | . | . | . | . | . | . | 99.26 | . | . | . | 99.83 | . | . | . | 99.58 | . | 99.61 | . | . |
| `FAL01198` | 90 | . | . | . | . | . | . | . | . | 99.70;100.00;99.70 | 99.62 | . | 99.87 | 99.87 | . | . | . | . | . | . | 99.88;99.88;99.88;99.88;99.88 | . | 99.51 | 99.88;99.88;99.88;99.88;99.88 | 100.00 | . | . | . | . | 100.00 | . | . | 98.38 | 100.00;100.00;100.00;100.00;100.00;100.00;97.44;100.00;100.00;100.00;100.00;100.00 | . | . | 100.00 | . | 100.00 | . | 99.88 | 99.77;99.88 | . | . | . | 99.65;99.65;99.65 | . | 99.72;99.72;99.63 | 99.55 | . | 100.00;100.00;100.00 | . | 99.29 | . | . | 99.57;99.61;99.64 | 100.00 | . | 99.60 | 98.95 | . | 99.79 | 99.13;99.63 | 99.82 | 99.92 | . | . | . | 99.86 | . | . | . | . | . | . | 99.64 | 99.72 | . | 100.00;99.82 | 100.00;99.81;100.00 | 99.36 | 100.00 | 99.84;99.92;99.84 | 100.00 | . | 99.71 | . | 99.76 | . | 99.82;99.73 | 99.92;99.86 | 99.84;99.76;99.76 | 99.90;100.00;99.90 | 99.82;99.91 | 99.71;99.71;100.00;99.71;99.42;100.00;100.00;100.00;98.25 | 99.54;99.54 | 99.58;99.47 | 99.89;99.83 | 98.43;99.55 | 99.77 | . | 100.00 | . | . | 100.00 | 100.00 | 99.71;99.76 | 99.84;99.76;99.76;99.92;99.84;98.13 | . | 100.00 | . | . | 96.32 | 100.00 | 100.00 | . | . | . | . | . | 99.16 | . | . | . | 90.82 | . | . | . | . | . | . | . | 99.49 | 100.00 | 100.00 | 99.40 | . | 100.00 | . | . | . | . | . | . | . | 99.65 | 99.92 | 99.78 | 100.00 | 100.00 | 99.31 | 99.85 | 99.66 | . | 96.80 | . | 99.79 | 97.04 | 100.00 | . | . | 100.00 | 99.63;99.63;99.51;99.63 | 99.87 | 99.57 | 99.83 | 99.92;99.83;99.83;99.92;99.83;99.75 | 99.92;99.83;99.83;99.83;99.83 | 99.91;99.83 | 99.79 | . | 99.71 | . | 99.72 | 99.78 |
| `FAL01556` | 54 | . | . | . | . | . | . | . | . | 100.00;100.00;99.70;99.70 | . | . | . | . | . | 99.87 | 99.90 | 81.09 | . | . | 99.88;99.88;99.76 | . | . | 99.88;99.88;99.52 | 100.00 | . | . | . | . | 100.00 | . | . | 98.71 | 100.00;100.00;100.00 | . | 99.89 | . | . | 99.91 | . | . | 99.77;99.88 | . | . | 99.77 | . | . | 99.72 | . | . | 99.51;99.51;99.51 | . | . | . | . | 99.54 | 99.68 | . | . | . | 98.95 | . | . | 99.65 | 100.00 | . | . | . | . | . | . | . | . | . | . | . | 99.58;99.65 | . | 100.00 | . | 99.76 | 100.00 | 99.84;99.75;99.84;99.84;99.75 | . | . | . | . | 99.76 | . | 99.82 | 99.95 | 99.84;99.76 | 99.90;99.79 | 99.86;99.82;99.31 | 99.12;100.00;100.00;97.66 | 99.54;99.54 | 99.68;99.37 | 99.77;99.83 | 98.21;99.48 | 99.82;99.77 | . | 100.00;100.00 | 98.51 | . | . | 100.00 | 99.62;99.67 | 99.92;98.22;99.84;98.22 | . | . | . | . | . | . | . | 98.68 | . | . | . | . | . | . | . | . | . | . | . | . | . | . | . | . | 98.93 | 100.00 | . | . | . | 100.00;100.00 | . | . | . | . | . | . | . | . | . | . | . | . | . | . | . | . | . | . | . | . | . | . | . | 99.88 | 99.63;99.63;99.75 | . | 99.57 | 99.83 | 99.92;99.92;99.92;99.92;99.92;99.92;93.92;99.58 | 99.92;99.83 | . | . | . | 99.73 | . | . | . |
| `FAL05306` | 89 | . | . | . | . | . | . | . | . | 100.00;99.40;99.40 | . | . | 99.87 | 98.74;95.20 | 99.24 | 99.62 | 99.81 | . | . | . | 99.88 | . | . | 99.88 | . | . | . | . | . | 100.00 | . | . | 98.38 | 100.00;100.00;100.00;100.00;100.00;100.00;100.00;100.00;100.00 | . | . | . | . | 99.82 | . | . | . | . | 99.54 | . | . | . | 99.72 | . | . | 99.88;99.88 | . | . | 99.21 | . | 99.64 | 99.36 | 99.58 | 96.99;96.18 | . | 99.58 | . | . | 99.59 | 99.75 | 99.44 | . | 97.93 | 98.92 | 99.83 | 99.58 | . | . | . | . | . | 99.58 | . | . | . | 99.36;99.34;99.29 | 100.00 | 99.84;99.92 | . | . | 99.67;99.71;99.32 | . | . | 98.19 | 99.73 | 99.89 | 99.59 | 99.90;99.48 | 99.86 | 99.42;99.71;86.41;99.42;100.00;99.42 | 99.42 | 99.68 | 99.83 | 99.48 | 99.68 | . | 100.00 | . | 100.00 | 100.00 | 100.00 | 99.57;99.62 | 99.84 | . | 100.00 | . | . | 96.32 | 99.72 | 99.78 | . | . | . | 99.18 | . | 98.88 | 99.23 | 99.01 | 98.92 | 85.03 | . | . | . | . | . | . | . | 99.37 | 100.00 | 99.40 | 96.70 | . | 99.85;99.85 | . | . | . | 99.81 | . | . | . | 99.62 | 99.77 | 99.57 | 99.77 | 99.72 | 99.43 | 99.85 | 99.53 | . | 97.72 | . | . | . | . | . | . | 99.76;99.76 | 99.51 | 98.86 | 99.57 | 99.83 | 99.92 | 99.83 | 99.75 | 98.59;99.48 | 98.33 | 99.56 | . | 99.72 | 99.78 |
| `FAL11145` | 59 | . | . | . | . | . | . | . | . | 99.40;100.00 | . | . | 98.99 | . | . | . | 99.77 | 81.04 | . | . | 99.88;99.52;99.76;99.03;99.88 | . | 99.14 | 99.88;99.88;100.00;99.40;99.28 | 99.63 | . | . | . | . | 100.00 | . | . | 98.71 | 100.00;100.00;99.15;100.00 | . | . | . | . | 99.74 | . | . | . | . | . | . | 99.65 | . | 99.72 | . | . | 100.00;99.88 | . | . | . | . | 99.44;99.57 | . | 98.10 | . | . | . | . | . | 99.65 | 99.92 | . | . | . | . | . | . | . | . | . | . | . | 99.65 | . | 100.00;100.00 | 99.52;99.81 | 99.32 | 100.00 | 99.75;99.51;99.18;99.75 | 97.40 | . | 99.64 | . | . | . | 99.46 | 99.89 | 99.76 | 100.00 | 99.68 | 100.00;99.71 | 99.42 | 99.47 | 99.83 | 99.48 | 99.73 | . | 100.00 | . | 99.83 | 100.00 | 98.68 | 99.48 | 99.92 | . | . | . | . | . | . | . | . | . | . | . | . | 98.81 | 98.85 | 98.81 | 97.44 | 90.65 | . | . | . | . | . | . | . | 98.06 | . | . | . | . | . | . | . | . | 98.10 | . | . | . | . | . | . | . | . | . | . | . | . | . | . | . | . | . | . | . | . | 99.75;99.63;99.26;99.63 | . | 99.79 | 99.83 | 99.83;99.75;99.50;99.67 | 99.83 | . | . | . | 99.49 | . | 99.78 | 99.50 |
| `FAL11151` | 44 | . | . | . | . | . | . | . | . | 100.00;99.70;99.40 | . | . | . | . | . | . | 99.87 | 81.05 | . | . | 99.52;99.28 | . | . | 99.88;99.76 | 99.63 | . | . | . | . | . | . | . | 98.71 | 100.00;100.00;100.00;100.00;100.00;100.00 | . | . | . | . | 99.82 | . | . | 99.54 | . | . | . | . | . | . | . | . | 100.00 | . | . | . | . | . | . | . | . | . | . | . | . | 99.76 | 99.75 | . | 99.47 | . | . | 100.00 | 99.89 | . | . | . | . | . | 99.65 | . | . | 99.42 | 99.29 | 100.00 | . | . | . | . | . | . | . | 99.64;99.73 | 99.84;99.84 | 99.59;99.51 | 99.90;99.90 | 99.82;99.86 | 99.71;99.71;100.00 | 98.61;98.32 | 99.26;99.05 | 99.54 | 98.88 | 99.68 | . | 100.00;100.00 | 99.63 | . | 100.00 | . | . | 99.84 | . | . | . | . | . | . | . | . | . | . | . | . | . | . | . | . | . | . | . | . | . | . | . | . | 98.20;99.42 | . | . | . | . | . | . | . | . | . | . | . | . | . | . | . | . | . | . | . | . | . | . | . | . | . | . | . | . | . | 99.51;98.77 | . | 98.72 | 99.48 | . | 99.92 | 99.49 | . | 98.89 | . | . | 99.45 | 99.72 |
| `FAL11305` | 57 | . | . | . | . | . | . | . | . | 100.00;99.70 | . | . | 99.87 | . | . | . | 99.84 | 81.07 | . | . | 99.88;99.88 | . | 98.16;99.63 | 99.88;99.88 | 100.00 | . | . | . | . | 100.00;99.72 | . | . | 98.38 | 100.00;100.00;100.00;100.00;100.00 | . | 99.77 | . | 100.00 | . | . | . | . | . | . | 100.00 | . | . | 99.82 | 97.12 | . | 100.00 | . | . | . | . | 99.57 | 99.36 | 100.00 | . | . | . | . | . | 99.71 | 99.83 | . | . | . | . | . | . | . | . | . | . | . | 99.72 | . | 99.82 | 99.90 | . | 100.00 | 100.00;99.92 | . | . | 99.61 | . | 99.67 | . | 99.64 | 99.84 | 99.84;99.84 | 100.00;99.90 | 99.82 | 100.00;99.12 | 99.60;99.36 | 99.58;99.58 | 99.83;99.77 | 98.51;99.40 | 99.82 | . | 100.00 | 99.63 | 100.00 | 100.00 | 99.56;100.00 | 99.52;99.62;99.76 | 99.68;98.13 | . | . | . | . | . | . | . | 100.00 | . | . | . | . | . | . | . | . | . | . | . | . | . | . | . | . | . | . | 99.70 | . | . | . | . | . | . | 99.62 | . | . | . | . | . | . | . | . | . | . | . | . | . | . | . | . | . | . | . | . | 99.63;99.63 | . | 99.57 | 100.00 | 99.83;99.83 | 99.83;99.83 | . | 99.38 | . | 99.78 | . | . | . |
| `FAL11351` | 81 | . | 100.00 | . | . | . | . | . | . | 100.00;100.00 | . | . | 99.87 | . | . | . | 99.94 | . | . | . | 99.88 | . | 99.88 | 84.95;99.88 | 99.63 | . | . | . | . | 100.00 | . | . | 98.71 | 100.00;100.00;100.00;100.00;100.00;100.00 | . | . | . | . | 99.91 | . | 99.77 | 99.88 | 99.77 | . | . | . | 99.81 | 99.72 | . | . | 99.88 | . | . | . | . | 99.70 | 99.68 | 99.79 | . | . | . | . | . | 99.82 | 99.92 | 99.34;99.29 | 99.65 | . | 99.59 | 100.00 | 99.79 | . | . | . | . | . | 99.44 | . | 99.82 | 99.81 | 99.65 | 100.00 | 99.92 | 100.00;100.00 | . | 99.87;99.74 | 99.12 | 98.90 | . | 99.73;99.64 | 99.92;92.32 | 99.76 | 99.90;99.79 | 99.86;99.77 | 99.71;86.41;99.71;100.00;100.00;100.00;99.71;99.71 | 99.60;99.31;99.36 | 99.26;99.58;99.58 | 99.83;99.77;99.77 | 97.91;99.55;99.63 | 99.18;99.73 | 99.37 | 100.00;100.00 | . | . | 100.00 | 100.00 | 99.57;82.26;99.62 | 99.84;99.84 | . | . | . | . | . | . | . | . | . | . | 99.39 | 99.90 | . | . | . | . | . | . | . | . | . | . | . | . | 99.51;99.66;95.85 | 100.00 | . | . | . | . | . | 100.00 | . | 100.00 | . | . | . | 99.65 | 99.61 | 99.78 | 99.77 | 100.00 | . | 100.00 | 99.73 | . | 99.09 | . | . | . | . | . | . | 99.88 | . | 100.00 | 99.79 | 100.00 | 99.83;99.83 | 99.67 | 99.83 | . | 99.32 | 99.73 | 99.61 | 99.72 | 99.83 |
| `FAL11384` | 90 | . | . | . | . | . | . | . | . | 100.00;99.70;99.70 | 98.34 | . | 99.87;99.75;99.75 | 99.75 | . | . | 99.90 | . | . | . | 99.88;99.76;99.88;99.88;99.88;99.88;99.88 | 99.62 | 99.75;99.88 | 99.88;99.64;100.00;99.88;99.88;99.88;99.88 | 100.00 | . | . | . | . | 100.00 | . | . | 98.71 | 100.00;100.00;100.00;100.00;100.00;100.00;100.00 | . | . | . | . | 99.91 | . | . | . | . | . | 99.88 | 99.77;99.65 | 99.61 | 99.72 | . | . | 100.00;100.00 | . | 99.76 | . | . | 99.54 | 100.00;100.00 | 99.79 | . | . | 98.95 | 99.16 | . | 99.82;99.88 | 99.83 | . | . | . | . | . | . | . | . | 99.71 | . | . | 99.72;99.65 | . | . | 99.81 | 99.69 | 100.00 | 99.84;100.00;99.84;99.75 | 99.76 | . | 99.74;99.41;99.77;99.77 | . | 99.71 | . | 99.82;99.82 | 99.89;99.92 | 99.84;99.84;99.84 | 99.90;99.90;99.90 | 99.72;99.77 | 100.00;99.71;99.42;98.25;99.12;99.71 | 99.48;99.65 | 99.58;99.79 | 99.83;99.89 | 99.55;98.51 | 99.86 | . | 100.00 | . | . | 100.00 | . | 99.81;99.86 | 99.68;98.22;99.68;99.76;99.84 | 80.60 | 100.00 | 100.00 | 99.58 | 96.55 | 100.00 | . | 99.67 | . | . | . | . | . | . | . | . | . | 99.12 | 99.89 | 98.95 | 99.89 | 99.77 | 99.71 | 99.93 | 99.51 | 99.71 | 100.00 | 100.00;99.40 | 99.84;99.84 | . | . | . | . | 100.00 | . | . | . | 99.59 | 92.03 | . | . | . | 99.56 | . | . | . | . | 99.79 | 99.48 | 97.78 | . | 100.00 | . | 99.88 | 99.51;99.51;99.51;99.63 | 99.75;99.75;99.75 | 99.57 | 99.66 | 99.92;99.58;99.92;99.42;99.83;99.67;99.58;99.92 | 99.83;99.75;99.83;99.83 | 99.75 | 99.84;99.74 | . | 99.66 | . | 99.89 | 99.83 |
| `FAL11387` | 58 | 99.35 | 100.00 | 100.00 | 99.77 | 99.60 | 100.00 | 99.89 | 99.55 | 100.00;99.70;100.00;99.70 | . | . | . | . | . | . | . | 83.46 | . | 88.91 | 99.88 | . | . | 99.88 | 99.63 | . | . | . | . | 100.00 | . | . | 98.38 | 100.00;100.00;99.15;100.00 | . | . | . | . | 99.82 | . | . | . | . | . | . | 99.65 | . | . | . | 100.00 | 99.51 | 99.53 | . | . | . | 99.41 | 99.36 | . | . | . | . | . | . | 99.82 | 99.75 | . | . | . | . | . | . | . | . | . | 99.24 | . | . | . | 99.64 | 99.32 | 99.36 | 100.00 | 99.84 | . | . | . | 99.64 | . | 99.51 | 99.82;99.73 | 99.86;99.86 | 99.76;99.84 | 99.69;99.90 | 99.82 | 99.71;99.42;99.71;99.42;86.41 | 99.13 | 98.84 | 99.77 | 98.28 | 99.73 | . | 100.00 | . | . | . | . | 95.61 | 99.84 | . | . | . | . | . | . | . | . | . | . | . | . | . | . | . | . | . | . | . | . | . | . | . | . | 99.73;99.39 | . | . | . | . | 99.85 | . | . | . | . | 99.44 | . | . | . | . | . | . | . | . | . | . | . | . | . | . | . | . | . | . | . | 99.51 | . | . | . | 99.92;99.92 | 99.59 | . | . | 99.21 | . | 99.69 | 99.72 | 99.78 |
| `FAL11417` | 55 | . | . | . | . | . | . | . | . | 99.70;99.70;100.00;100.00 | . | . | . | . | . | . | 99.90 | . | . | . | 99.88;99.88 | . | 99.75 | 99.88;99.88 | . | 99.77 | 100.00 | 100.00 | . | 100.00 | . | 92.02 | . | 100.00;100.00;100.00 | . | . | . | . | . | . | . | . | . | . | . | 99.65 | . | 99.72 | . | . | 99.38 | . | . | . | . | 99.47;99.54 | 99.68 | 100.00 | . | . | . | . | . | 99.47 | 99.83 | . | . | . | . | . | . | . | . | . | . | . | 99.72;99.51 | . | 100.00;99.45 | 99.90 | 99.34 | 99.89 | 100.00;99.92;99.42;99.92 | 99.53 | . | 99.74;99.61 | . | . | . | 99.82 | 99.86 | 99.84;99.84 | 99.58;100.00 | 99.82 | 99.71;97.66;99.71;100.00;99.71;99.42 | 99.48;99.36 | 99.68;99.68 | 99.77;99.83 | 99.55;98.36 | 99.82 | . | 100.00 | . | 100.00 | 100.00 | 99.56;100.00;98.25 | 99.71;99.62 | 98.22;99.84 | . | . | . | . | . | . | 99.89 | . | . | . | . | . | . | . | . | . | . | . | . | . | . | . | . | . | 99.34 | . | . | . | . | . | . | . | . | 99.81 | . | . | . | . | . | . | . | . | . | . | . | . | . | . | . | . | . | . | . | . | 99.39;99.63 | . | 99.79 | 99.83 | 99.92;99.92;99.75;99.92 | 99.83;99.75 | 99.83 | 99.74 | . | 99.76 | . | . | . |
| `FAL11425` | 32 | . | . | . | . | . | . | . | . | 100.00;99.70;100.00;99.70 | . | . | . | . | . | . | 99.87 | . | . | . | . | . | . | . | 100.00 | . | . | . | . | 100.00 | . | . | 98.71 | 100.00;100.00 | . | . | . | . | 99.74 | . | . | . | . | . | . | . | 99.81 | . | . | . | 99.88 | . | . | . | . | 99.67 | . | . | . | . | . | . | . | 99.18 | 99.83;99.92 | . | . | . | . | . | . | . | . | . | . | . | 99.65 | . | 100.00 | 99.71 | 99.36 | 100.00 | . | . | . | . | . | . | . | . | . | . | . | . | 100.00 | 99.48 | 99.68 | 99.77 | 99.55 | 99.73 | . | 100.00 | . | . | 100.00 | . | . | 99.92 | . | . | . | . | . | . | . | . | . | . | . | . | . | . | . | . | . | . | . | . | . | . | . | . | 99.64;99.59;99.66 | . | . | . | . | . | . | . | . | . | . | . | . | . | . | . | . | . | . | . | . | . | . | . | . | . | . | . | . | . | . | . | . | . | 99.92 | . | 99.23 | . | . | . | 99.71 | 99.72 | 99.61 |
| `FAL16059` | 56 | . | . | . | . | . | . | . | . | 100.00;99.70 | . | . | 99.87 | . | . | . | 99.94 | . | . | . | 99.88;99.88;99.88;99.88 | . | . | 100.00;99.88;99.76;99.88 | 99.63 | . | . | . | . | 100.00 | . | . | 98.06 | 100.00;100.00;100.00 | . | . | . | . | 100.00 | . | . | . | . | . | . | 99.77 | . | 99.72;99.82 | . | . | 100.00 | . | . | . | . | 99.54 | 100.00 | 99.79 | . | . | . | . | . | 99.59 | 99.83 | 99.63 | . | . | . | . | . | . | . | . | . | 99.18 | 99.51;99.58 | . | 100.00;100.00 | 99.81;99.81 | 99.29 | 100.00 | 99.75;99.92 | . | . | 99.71 | . | 98.52 | . | 99.73 | 99.86 | 99.84;99.84 | 99.69;99.90 | 99.72 | 99.71;99.71;99.71;99.71;99.71;86.41 | 99.60;99.54 | 99.47;99.58 | 99.89;99.83 | 98.21;99.55 | 99.86;99.73 | . | 100.00;100.00 | . | . | 100.00 | 100.00;100.00 | 99.67;99.62 | 98.13;99.76 | . | . | . | . | . | . | 99.78 | . | . | . | . | . | . | . | . | . | . | . | . | . | . | . | . | . | 99.20 | . | . | . | . | . | . | . | . | 100.00 | . | . | . | . | . | . | . | . | . | . | . | . | . | . | . | . | . | . | . | . | 99.51;98.53;99.51;99.63 | . | 99.79 | 99.66 | 99.92;99.92;99.83 | 99.83;99.83;99.75 | . | 99.58;99.64 | . | 99.59 | . | 99.89 | 99.78 |
| `FAL17459` | 62 | . | . | . | . | . | . | . | . | 100.00;99.70;100.00;99.70 | . | . | . | 100.00 | . | . | 99.90 | 99.90 | . | . | 99.88 | . | . | 99.88 | 100.00 | . | . | 100.00 | . | . | . | . | 98.06 | 100.00;100.00 | . | . | . | . | . | 99.82 | . | . | . | . | . | 99.65;99.88 | . | 99.82 | . | . | 99.01 | . | . | . | . | 99.70 | 99.68 | . | 99.60 | . | . | . | . | 99.59 | 99.83 | . | . | . | . | 99.83 | 99.48 | . | 99.37 | . | . | . | 99.65 | . | . | 96.71 | 99.81 | 100.00 | 99.34 | . | 99.64 | 99.64 | 99.78;99.85 | . | . | 99.46;99.73 | 99.86;99.95 | 99.76;99.84 | 99.90;99.79 | 99.82;99.86 | 99.71;99.42;100.00;100.00 | 99.31 | 99.47 | 91.16;99.71 | 98.58;98.21 | . | . | 100.00 | . | . | 100.00 | 97.81 | 99.57;99.67 | 99.84;99.84 | . | . | . | . | . | . | . | . | . | 99.39 | . | . | 98.81 | 99.43 | 99.01 | 98.03 | 91.16 | . | . | . | . | . | . | . | 99.68 | . | . | . | . | 99.70 | . | . | . | . | . | 97.83 | . | . | . | . | . | . | . | . | . | 99.93 | . | . | . | . | . | . | . | . | 99.63 | 99.87 | . | . | 99.67;99.83 | . | . | . | 99.70 | . | 99.66 | 99.72 | 99.78 |
| `FAO71993` | 84 | . | . | . | . | . | . | . | . | 100.00;99.70;100.00;99.70 | . | . | 99.24 | . | . | . | 99.87 | . | 98.62 | . | 99.76;99.03;99.88 | . | 99.39;99.39 | 99.88;99.76;99.88 | 99.63 | 99.37;99.71 | 99.84 | 99.53;99.77 | 100.00 | 100.00 | 99.44 | 98.58 | 99.68 | 100.00;100.00;100.00;100.00;100.00;100.00 | . | . | . | . | 99.82 | . | . | 100.00;100.00 | . | . | . | . | . | 99.91 | . | 99.15 | 99.75 | . | . | . | . | 99.51;98.52 | 99.68 | 99.58 | . | 98.95 | . | . | . | 99.65 | 99.75;99.92 | . | . | . | 99.86 | 100.00 | . | . | . | . | . | . | 99.65 | . | 99.82 | 99.71 | 99.39 | 100.00 | 99.75;99.84 | 92.20 | . | 99.77 | 99.78 | . | . | 99.73 | 99.84 | 99.84 | 99.79;99.79 | 99.82 | 99.71;100.00;99.71;99.71;99.71 | 99.36 | 99.47 | 99.71 | 98.43 | 99.45 | . | 100.00 | . | 100.00 | 100.00 | . | 99.52 | 99.59;99.68 | . | 99.29 | . | . | 95.63 | 98.86 | 100.00 | . | . | . | . | . | . | . | . | . | . | 99.78;99.50;99.39 | 99.66;99.89;100.00 | 100.00;99.74;99.74 | 99.57;99.46;99.78 | 99.54;99.54 | 99.85;100.00 | 99.86 | 99.76 | . | . | . | . | . | . | . | . | 100.00 | . | . | . | 99.65;99.62 | 99.54;99.23 | 99.71 | 99.77 | 99.72;100.00 | 99.52;99.31 | 100.00 | 99.66 | . | . | . | . | . | . | . | . | . | 99.51;99.14;99.26 | . | 100.00;99.79;99.79 | 99.83;99.83;99.14 | 99.92;99.83;99.92;99.92 | 99.75;99.83;99.83 | 99.58 | . | 98.58 | . | 99.13;95.16 | . | . |
| `FAO72169` | 77 | 98.69 | 99.82 | 98.04 | 98.83 | 99.20 | 98.19 | 96.61 | 97.86 | 99.70;99.70;100.00;100.00 | . | . | . | . | . | . | 99.94 | . | . | . | 99.64;99.52 | . | . | 99.88;99.64 | 99.63 | . | . | . | . | 100.00 | . | . | 98.71 | 100.00;100.00;100.00;100.00;82.91;100.00 | 99.47 | . | . | . | 99.91 | . | . | 99.88;99.65 | . | . | . | . | . | 99.36 | . | 99.39;99.76 | 99.14 | 99.53 | . | . | . | 99.47 | 100.00;99.68 | . | . | . | . | 99.16 | 99.40 | 99.71 | 99.83 | . | 99.05 | . | . | 99.83;99.83;100.00 | 99.68;99.69;99.79 | 98.70 | 99.36;96.47 | . | 99.85 | 99.77 | 99.30;99.65 | . | 99.64 | 99.61 | 99.32 | 100.00 | 98.68 | 99.76 | . | 99.45 | 99.78 | 99.43 | . | 99.82;99.82;99.82 | 99.15;99.81 | 99.67;99.76 | 99.69;100.00;99.58 | 99.77;99.77 | 99.12;99.71;100.00;98.83;99.71;100.00 | 99.31;99.54 | 99.68;99.68 | 99.71;99.66 | 99.33;98.58 | 99.50 | . | 100.00;100.00 | . | 100.00 | 100.00 | . | 99.67 | 99.92 | . | . | . | . | . | . | . | . | 99.62 | . | 99.80 | 99.90 | . | . | . | . | . | . | . | . | . | 99.08 | . | . | 99.59 | . | . | . | . | . | . | . | 96.00 | . | 99.54 | . | . | . | . | . | . | . | . | . | . | . | . | . | . | . | . | . | . | . | 99.63;99.51 | . | . | . | 99.92 | 99.67;99.67 | . | . | 99.69;99.69 | 99.47 | 99.61 | 99.72 | 99.83 |
| `FAO96374` | 77 | . | . | . | . | . | . | . | . | 99.70 | . | . | 99.87 | 99.62 | . | 99.87 | 99.94 | 81.09 | . | . | 99.88;99.88;99.88;99.88;99.88;89.01 | . | 99.51 | 99.88;99.88;99.88;99.88;99.88;99.88;84.71 | 99.63 | 99.77 | 99.92 | 100.00 | . | 100.00 | . | 98.86 | 98.71 | 100.00;100.00;100.00;100.00;100.00;100.00;100.00 | . | . | . | . | 99.82 | . | . | 99.42 | . | . | . | 99.77 | 99.81 | 99.91 | . | . | 99.51;100.00 | . | . | . | . | 99.67;99.74 | 99.36 | 100.00 | . | 99.37;98.52 | 98.31 | . | . | 99.71 | 99.83 | . | . | . | 99.73 | . | . | . | . | . | . | . | 99.58 | 99.54 | 100.00 | 100.00 | 99.34 | 100.00 | 99.84;99.75;98.60;99.92 | . | . | 99.48;99.58;99.80 | . | . | 99.51 | 99.55 | 99.81 | 99.67 | 99.90;99.90 | 99.72 | 100.00;97.66;97.37;100.00;99.42 | 99.60 | 99.68 | 99.77 | 99.85 | 99.73 | . | . | . | 100.00 | 100.00 | 99.56 | 94.71;99.43 | 100.00 | . | . | . | . | . | . | 100.00 | . | . | . | . | . | 99.28 | 100.00 | 99.01 | 99.51 | 90.99 | . | . | . | . | . | . | . | 99.56 | 100.00 | 99.40 | . | . | 98.48 | 100.00 | . | . | 100.00 | . | . | . | . | . | . | . | . | . | . | . | . | . | . | . | . | . | . | . | 99.88 | 99.63;99.51;99.63 | . | 99.57 | . | 99.92;99.83;99.92;99.67 | 99.83;99.75 | . | 99.69 | . | 99.73 | . | 99.72 | 99.78 |
| `FAO97359` | 70 | . | . | . | . | . | . | . | . | 100.00;99.40 | . | 100.00 | 99.49;99.87 | 99.87;99.62;99.87 | . | . | 99.94 | . | . | . | 99.88;99.88 | . | 99.63 | 99.88;99.76 | 99.63 | . | . | . | . | 100.00 | . | . | 98.71 | 100.00;100.00;100.00;100.00;100.00;100.00 | 99.61 | . | . | . | 99.74 | . | . | . | . | . | . | . | . | 99.63 | . | . | 100.00 | . | 98.41 | . | . | 99.61 | 98.72 | . | 98.80;99.00;99.60 | . | . | . | . | 99.65 | 99.92 | . | . | . | . | 99.65 | 99.47 | . | 84.97 | . | . | . | 99.72 | . | 99.45;99.82;99.26 | 99.71 | 99.39 | 100.00 | 99.75;99.84 | . | . | 99.48 | 99.71 | 99.47 | . | 99.73 | 99.81 | 99.67 | 99.90 | 99.86 | 97.66;100.00;100.00;99.42 | 99.31;99.31 | 99.68;99.58 | 99.77;98.97 | 97.76;99.18 | 99.59 | . | 100.00;100.00 | . | 100.00 | . | . | 99.48 | 99.76 | 99.58 | . | . | . | . | 98.86 | 98.59 | . | 99.39 | . | . | . | . | . | . | . | . | . | . | . | . | . | . | . | 84.89;99.56 | 99.71;99.71 | . | 98.80 | . | 99.85 | . | . | . | . | . | . | . | . | . | . | . | . | . | . | . | . | . | . | . | . | . | . | . | 99.29;99.76 | 99.63;99.63 | 99.62;99.62 | 99.57;99.36;98.93 | 99.66;99.83;99.83 | 99.50;99.75;99.50;99.83 | 99.83 | 99.49 | . | 99.57 | 99.47 | 99.59 | 99.67 | 99.83 |
| `FAQ33890` | 84 | . | . | . | . | . | . | . | . | 99.40;100.00 | 100.00 | . | 99.87 | . | . | . | 99.81 | . | . | . | 99.76;99.76;99.88;99.88;99.88 | . | 99.51;99.51 | 99.88;99.88;100.00;99.88;99.88 | 99.63 | . | . | . | . | 100.00 | . | . | 98.71 | 100.00;100.00;100.00;100.00;100.00 | . | . | . | . | 99.65 | . | . | 99.77 | . | . | . | . | . | 99.72 | . | . | 99.88;99.88;100.00 | . | . | . | . | 99.70 | 99.68 | 100.00 | . | . | . | . | . | 99.65;99.71 | 99.83 | 99.58 | 99.82 | . | . | 100.00 | 98.76;99.68 | . | 80.99;98.31 | . | 98.62 | . | 99.44 | . | . | 99.90 | 99.29 | 99.89;100.00 | 99.92;99.18;99.92;100.00 | . | . | 99.71;99.67 | 98.91 | 99.71;99.71;98.37 | . | 99.73 | 99.92 | 99.84 | 100.00;100.00 | 99.82 | 99.71;100.00;99.42;100.00;100.00;100.00;99.42;100.00;99.71;86.41 | 99.48;99.54 | 99.68;99.68 | 99.71;99.83 | 99.63;99.55 | 99.68;99.73 | . | 100.00 | . | 100.00 | 100.00 | 100.00 | 99.62;99.62 | 99.84 | . | 100.00 | . | . | 94.25 | 100.00 | . | . | . | 99.19 | . | 99.40 | 98.53 | 98.85 | 98.61 | 99.31 | 90.48 | . | . | . | . | . | . | . | 99.66 | . | 100.00 | . | . | 99.85 | . | . | . | 100.00 | . | . | . | 98.60 | 99.61 | 99.28 | 99.77 | 99.72 | . | 99.56 | 98.85 | . | . | . | . | . | . | . | . | . | 99.51;99.63;99.63 | 99.62 | 99.79 | 99.83 | 99.33;99.75;98.83;97.50;99.92;99.92 | 99.75;99.67 | 99.91 | 99.58 | 99.57 | 99.71 | . | 99.72 | 99.83 |
| `FAQ33923` | 104 | . | . | . | . | . | . | . | . | 100.00;100.00;99.40 | 99.87 | . | 99.87;99.75 | 99.75 | . | 98.86 | 99.77 | . | . | . | 99.88;99.76;99.88;99.64 | . | 99.51;99.51 | 99.88;99.88;99.88;100.00 | 99.63 | . | 99.92 | 99.30 | . | 99.44 | . | . | 98.38 | 100.00;100.00;100.00 | . | . | . | . | 99.47 | . | . | 99.77;95.01 | . | . | . | 99.77 | 99.42 | 99.82 | . | . | 99.88;99.26;99.88;99.88 | . | 99.60 | . | . | 99.28;99.44;99.51 | 99.04 | 99.58 | . | . | 98.31 | . | . | 99.35 | 99.92 | . | 99.54 | . | . | 100.00 | 99.58 | . | 97.06 | . | . | . | 99.37 | . | 99.45;100.00 | 99.52 | 99.32 | 100.00 | 99.92;99.67 | 90.78 | . | 85.37 | . | . | . | 99.46;99.64 | 99.75;99.81 | 99.67;99.67 | 99.79;100.00;99.69 | 99.77;99.59 | 100.00;99.12;99.71;97.37;99.42 | 99.13 | 99.58 | 99.66 | 99.40 | 99.64 | . | 100.00;100.00 | 99.81 | 100.00 | 100.00 | 100.00;100.00 | 99.67;99.52 | 99.84 | . | 99.05 | . | 97.89 | 98.16;93.10 | 95.44;99.72 | 99.67 | . | . | . | . | . | 98.85 | 99.62 | 98.61 | 99.11 | 90.65 | 99.45 | 99.66 | 99.74 | 99.35 | 99.77 | 99.71 | 99.57 | 99.39 | 99.71 | 100.00 | 100.00 | 99.84 | . | . | . | . | 99.81 | . | . | . | 99.56 | 99.61 | 99.71 | 99.54 | 100.00 | 99.19 | 99.56 | 99.46 | . | . | . | 98.54 | . | . | . | 99.26 | 99.52;99.88 | 99.51;99.51;99.63;99.51 | 99.62 | 99.57 | 100.00 | 99.92;99.75;99.58;99.00 | 99.67;99.83 | 99.83 | 99.38;98.59 | 99.07 | 99.59 | . | 99.78 | 99.67 |
| `FAQ34034` | 65 | . | . | . | . | . | . | . | . | 99.70;99.70;100.00 | 99.36 | . | 100.00 | . | . | . | 99.87 | 97.32 | . | . | 99.76;99.88;99.88;99.88 | . | 99.26 | 99.88;99.76;99.76;99.76 | 99.63 | . | . | . | . | 100.00 | . | . | 98.71 | 100.00;100.00;100.00;100.00;100.00 | . | . | . | . | 99.82 | . | . | . | . | . | 99.88 | 99.88 | 100.00 | . | . | . | 100.00 | . | . | . | . | 99.61 | 99.68 | 99.79 | . | . | . | . | . | 99.88 | 100.00 | 99.63 | . | 99.65 | . | 100.00 | 99.68 | . | 82.60 | . | . | . | 99.58 | . | 100.00;100.00 | 99.81;99.81 | 99.34 | 99.89 | 100.00;99.92 | . | . | 99.77;99.74 | . | 99.38 | . | 99.55 | 99.81 | 99.59 | 99.79;99.90 | 99.82 | 99.71;86.41;100.00;99.71;99.71 | 99.48;99.48 | 99.37;99.58 | 99.71;99.77 | 98.36;99.48 | 99.77 | . | 100.00;100.00 | . | . | . | . | 99.67 | 99.92 | . | . | . | . | . | . | . | . | . | . | . | . | 99.04 | 99.04 | 98.81 | 99.31 | 86.39 | . | . | . | . | . | . | . | 99.37 | . | 100.00 | . | . | . | . | . | . | 100.00 | . | . | . | . | . | . | . | . | . | . | . | . | . | . | . | . | . | . | . | . | 99.51;99.51;99.63;99.63 | . | 99.79 | 99.83 | 99.92;99.92;99.92;99.92 | 99.75 | . | . | . | 99.73 | . | 99.78 | 99.67 |

---

## ABRICATE\_MEGARES

The results table shown here is a collection from all samples.

| #FILE | NUM\_FOUND | AAC2-PRIME | AAC3 | ACRA | ACRB | ACRD | ACRE | ACRF | ACRS | AMPH | ANT3-DPRIME | APH3-DPRIME | APH3-PRIME | APH6 | ASMA | BACA | BAER | BAES | BCR | BLAEC | CATA | CMLA | CPXAR | CRP | CTX | DFRA | EMRA | EMRB | EMRD | EMRK | EMRR | EMRY | EPTA | ERMB | EVGS | FLOR | FOSA | GADW | GADX | HNS | KDPE | KPN | MARA | MARR | MDFA | MDTA | MDTB | MDTC | MDTE | MDTF | MDTG | MDTH | MDTI | MDTJ | MDTK | MDTM | MDTN | MDTO | MDTP | MPHA | MPHB | MSBA | MVRC | OXA | PBP2 | PBP4B | PMRF | QACG | QACL | QNRB | QNRS | ROBA | SHV | SOXS | SULI | SULII | SULIII | TEM | TETA | TETB | TETC | TETD | TETM | UGD | YOGI |
| --- | --- | --- | --- | --- | --- | --- | --- | --- | --- | --- | --- | --- | --- | --- | --- | --- | --- | --- | --- | --- | --- | --- | --- | --- | --- | --- | --- | --- | --- | --- | --- | --- | --- | --- | --- | --- | --- | --- | --- | --- | --- | --- | --- | --- | --- | --- | --- | --- | --- | --- | --- | --- | --- | --- | --- | --- | --- | --- | --- | --- | --- | --- | --- | --- | --- | --- | --- | --- | --- | --- | --- | --- | --- | --- | --- | --- | --- | --- | --- | --- | --- | --- | --- | --- | --- |
| `FAL00958` | 60 | . | . | 100.00 | 99.90 | 99.74 | 99.83 | 99.87 | 100.00 | 99.65 | . | . | . | 99.88 | 99.84 | 99.76 | 100.00;99.86;99.72;99.72 | 99.93;99.64;99.72;98.86 | 100.00 | 100.00 | . | . | 99.86;99.93 | 100.00 | 99.68;99.66 | 99.16 | 99.74 | 100.00 | 100.00 | 99.83 | 100.00 | 99.74 | 99.82 | . | 99.64 | . | . | 99.73 | 99.76 | 99.76 | 99.26 | 99.57 | 99.23 | 99.54 | 100.00 | 99.84;99.92 | 99.84;99.74 | 99.81;99.90;99.84 | 99.83 | 99.90 | 99.67;99.67 | 100.00 | 99.70 | 100.00 | 99.93 | 99.76 | 99.81 | 99.81 | 99.66 | . | 98.74 | 100.00 | 100.00;99.70;100.00 | . | 99.84 | 99.77 | 99.90 | . | . | . | . | 100.00 | . | 99.69 | . | 99.26 | . | . | 99.84 | . | . | . | . | 99.91;100.00;99.83;100.00;99.91;99.83;99.83;100.00;99.91;99.74;99.91;99.91 | 99.94 |
| `FAL01198` | 75 | 99.62 | . | 100.00 | 99.90 | 99.78 | . | . | . | 99.91 | 97.80;96.39 | 99.88;99.88;99.88;99.88;99.88;99.51 | . | 99.88;99.88;99.88;99.88;99.88 | 99.95;99.84;99.95;99.95;99.67;99.35 | . | 100.00;99.86 | 99.86;99.93 | 99.92 | 100.00 | 99.55 | 99.38 | 99.93;100.00 | 100.00 | 99.89;100.00;99.79;99.79;99.79;99.89;100.00;99.89;87.08;99.79;99.89 | 99.79;98.95;99.60 | 99.83 | 99.87 | 99.92 | 100.00 | 99.62 | 99.68 | 99.88 | 99.87 | 99.69 | 99.84;99.92;99.84 | 100.00 | 99.59 | 99.76 | 99.76 | 99.26 | . | 99.49 | 99.77 | 99.84 | 99.84;82.77;99.84 | 99.90;99.71;99.84 | 99.94;99.55 | 99.91 | 99.87 | 85.33 | 99.92 | 99.70 | 99.73 | 100.00 | 99.84;99.76;99.76;99.92;99.84;98.13 | 99.81 | 100.00 | 99.66 | 100.00 | 99.58 | 99.94 | 99.70;100.00;99.70 | . | 99.79 | 100.00 | 99.90 | 100.00 | 99.40 | . | 100.00 | 100.00;100.00 | 99.60 | 99.38 | 99.90 | 99.63;99.63;99.51;99.63 | 99.87 | 86.56;94.90;97.95;98.03;98.11 | 99.92;99.84;99.84;99.92;99.84;99.69 | 99.92;99.83;99.83;99.83;99.83 | 99.91;99.83 | 99.76;99.76;99.76;100.00;100.00 | 99.79 | 100.00;100.00 | 99.88;99.88 |
| `FAL01556` | 65 | . | . | 100.00 | 99.94 | 99.81 | 100.00 | 99.90 | 99.85 | 100.00 | 99.87 | 99.88;99.88;99.76 | . | 99.88;99.88;99.52 | 99.78 | 99.76 | 100.00 | 99.93 | 100.00 | 99.91 | . | . | 100.00;99.93 | 100.00 | 99.36;99.68;99.79;97.24;99.68;99.79;99.79;87.01;99.79;99.79;99.26;100.00;99.79;99.79;99.89;99.68;99.79 | 98.95 | 100.00 | 99.94 | 100.00 | 99.91 | 100.00 | 99.81 | 99.94 | . | 99.75 | 99.84;99.75;99.84;99.84;99.75 | . | 99.59;99.73 | 99.88;99.88 | 100.00 | 99.26 | . | . | . | 99.84;99.84 | 99.92 | 99.94 | 99.84 | 99.91;99.91 | 99.90;99.94 | 99.84 | 100.00 | 99.70 | 99.73 | 100.00 | 99.92;98.22;99.84;98.22 | 99.90 | 100.00 | 99.59 | . | 98.68;99.58 | 100.00 | 100.00;100.00;99.70;99.70 | . | 99.89 | 99.92 | 99.90 | . | . | . | 100.00;100.00 | 100.00 | . | 99.69 | 99.79 | 99.63;99.63;99.75 | . | 98.03;98.11;98.19 | 99.92;99.92;99.92;99.92;99.92;99.92;94.27;99.61 | 99.92;99.83 | . | 99.76 | . | 99.91;100.00;100.00;99.83;99.83;100.00;100.00;99.31;99.91;99.91;100.00 | 99.94 |
| `FAL05306` | 74 | . | . | 100.00 | 99.87 | 99.71 | 100.00 | 99.81 | 99.70 | 99.91 | 96.15;99.24;96.70;93.28;99.62 | 99.88 | . | 99.88 | 99.84 | 99.76 | 100.00 | 99.86 | 99.92 | 99.82 | . | 99.04 | 99.78;99.86 | 99.84 | 99.89;99.89 | 99.58;96.99;96.18;99.58 | 99.91 | 99.94 | 99.75 | 99.91;99.66;99.83;99.74;99.91 | 100.00 | 99.74;99.68;99.68;99.48;99.61 | 99.82 | 98.82 | 99.69;99.47;99.67;99.75;99.28 | 99.84;99.92 | . | 99.59 | 99.76 | 99.52 | 99.26 | . | 99.49 | 99.54 | 99.92 | 99.84 | 99.90 | 99.81 | 99.91 | 99.84 | 99.76 | 99.92 | 99.70 | 99.73 | 100.00 | 99.84 | 99.90 | 100.00 | 99.59 | 99.78 | 98.95 | 100.00 | 100.00;99.40;99.40 | . | 99.79 | . | 99.90 | 99.40 | 96.70 | . | 99.85;99.85 | 100.00 | . | 99.69 | 99.38;99.69 | 99.51 | 98.86 | 97.87 | 99.92 | 99.83 | 99.75 | 99.76 | 98.59;99.48 | 100.00 | 99.88 |
| `FAL11145` | 65 | . | . | 100.00 | 99.87 | 99.61 | 99.65 | 99.77 | 99.85 | 99.83 | 96.03 | 99.88;99.52;99.76;99.03;99.88 | 99.14 | 99.88;99.88;100.00;99.40;99.28 | 99.84;85.91 | 99.76 | 100.00 | 99.79 | 99.92 | 99.74 | . | . | 99.71;99.71 | 100.00 | 100.00;99.68 | 98.10 | 99.91 | 99.87 | 99.92 | . | 99.62 | 99.87 | 99.94;99.88 | . | 99.69 | 99.75;99.51;99.18;99.75 | . | 99.45 | 99.76 | 99.76 | 99.12 | . | 99.49 | 99.54 | 99.76 | 99.76 | 99.84 | 99.74 | 99.83 | 99.71 | 99.43 | 99.92 | 100.00 | 99.73 | 100.00 | 99.92 | 99.81 | 100.00 | 99.59 | . | 98.95 | 100.00;100.00 | 99.40;100.00 | . | 99.63 | 99.92 | 99.90 | . | . | . | . | 100.00 | . | 99.69 | . | 99.75;99.63;99.26;99.63 | . | 97.95 | 99.84;99.69;99.53;99.61 | 99.83 | . | 99.76 | . | 100.00;88.95 | 99.82 |
| `FAL11151` | 56 | . | . | 100.00 | 99.87 | 99.68 | 99.83 | 99.87 | 99.85 | 99.83 | . | 99.52;99.28 | . | 99.88;99.76 | 99.89;99.73;99.95 | 99.64 | 100.00 | 99.86 | 99.83 | 99.82 | . | . | 99.85;100.00 | . | . | . | 100.00 | 99.94 | 99.75 | 99.91 | 100.00 | 99.74 | 99.82 | . | 99.61 | . | . | . | . | 99.52 | 99.26 | . | 99.74 | 99.77 | 99.76 | 99.84;99.68 | 99.94;86.01 | 99.84 | . | . | 99.76 | 99.92 | 99.70 | 99.73 | 100.00 | 99.84 | 99.90 | 99.95 | 99.45 | . | 99.16 | 100.00 | 100.00;99.70;99.40 | . | 99.79 | 100.00 | 99.79 | . | . | . | . | 100.00 | . | 99.69 | . | 99.51;98.77 | . | 97.87 | . | 99.92 | 99.49 | 99.76 | . | 100.00;100.00;99.91;100.00;100.00 | 99.82 |
| `FAL11305` | 67 | . | . | 100.00 | 99.90 | 99.84 | 100.00 | 99.84 | 99.85 | 99.83 | 96.27 | 99.88;99.88 | 98.16;99.63 | 99.88;99.88 | 99.95;99.84;99.95 | 100.00 | 100.00;100.00 | 99.93;99.93 | 100.00;100.00 | 100.00 | 97.32 | . | 99.85;100.00 | 100.00 | 99.89;99.78;100.00;99.79;99.58;99.68;99.79;99.68;84.86;99.79;98.94 | 100.00 | 99.91;99.91 | 100.00;100.00 | 99.83 | 99.91 | 100.00;100.00 | 99.61 | 99.94 | . | 99.75 | 100.00;99.92 | . | 99.59;99.73;99.59 | 99.76;100.00;99.88 | 100.00 | 99.26 | . | 99.23;99.49 | 99.77;99.54 | 100.00 | 99.92;99.92 | 99.87;99.90 | 99.90;99.87 | 99.91;99.91;99.91 | 99.90;99.90;99.97 | 99.92 | 100.00 | 99.70 | 99.73 | . | 99.68;98.13 | 99.90 | 100.00 | 99.66 | . | 99.58;100.00 | . | 100.00;99.70 | . | 99.79 | 100.00 | 99.90 | 99.70 | . | . | . | 100.00 | . | 99.69 | . | 99.63;99.63 | . | 98.19 | 99.84;99.84 | 99.83;99.83 | . | 99.76 | 99.38 | 100.00 | 99.88 |
| `FAL11351` | 68 | . | . | 100.00 | 99.90 | 99.78 | 100.00 | 99.94 | 99.70 | 99.48;99.74 | 96.27 | 99.88;99.88 | . | 84.95;99.88 | 99.84 | 99.76 | 99.86;100.00 | 99.79;99.93 | 99.92 | 99.91 | . | . | 99.85;100.00 | 99.84 | 100.00;96.40;99.47;100.00;99.79;99.36;99.79 | 99.79 | . | . | 99.92 | 99.91 | . | 99.81 | 99.82 | 99.61 | 99.78 | 99.92 | 100.00;100.00 | 99.73 | 100.00 | 100.00 | 99.12 | . | 99.49 | 99.54 | 99.92;100.00 | 99.92 | 99.94;99.90 | 99.84;99.87 | 99.91 | 99.94 | 99.84 | 100.00 | 99.39 | 100.00 | 100.00 | 99.84;99.84 | 99.90 | 99.95 | 99.59 | . | 99.58 | 100.00 | 100.00;100.00 | . | 99.89 | 100.00 | 99.90 | . | . | . | 100.00 | 100.00 | 99.50 | 100.00 | 99.79 | . | 100.00 | 98.11;98.11 | 99.84;99.84 | 99.67 | 99.83 | 99.76 | . | 99.91;100.00;99.91;99.91;99.91;100.00;100.00;100.00 | 99.94 |
| `FAL11384` | 77 | 98.34 | . | 100.00 | 99.90 | 99.81 | 100.00 | 99.90 | 99.85 | 100.00 | 97.68;96.39;96.15;96.03 | 99.88;99.62;99.76;99.88;99.88;99.88;99.88;99.88;99.88 | 99.75 | 99.88;99.64;100.00;99.88;99.88;99.88;99.88 | 99.95;99.95 | 99.76 | 100.00;100.00 | 99.86;99.86 | 100.00 | 99.91 | . | 99.79 | 99.85;100.00 | 100.00 | 99.89;99.89;100.00;100.00;99.79;99.89;99.79 | 98.95;99.16;99.79 | 99.91 | 100.00 | 99.83 | 100.00 | 100.00 | 99.74 | 99.94 | . | 99.78 | 99.84;100.00;99.84;99.75 | 99.76 | 99.59 | 99.88 | 99.52 | 100.00 | . | 99.23 | 99.77 | 100.00 | 99.92;99.92 | 99.90;99.94 | 99.90;99.90 | 99.91 | 99.87 | 99.84 | 99.92 | 100.00 | 99.73 | 100.00 | 99.68;98.22;99.68;99.76;99.84 | 99.81 | 100.00 | 99.59 | . | 99.58;99.67 | 100.00 | 100.00;99.70;99.70 | 99.89 | 99.84 | 100.00 | 99.90 | 100.00 | 100.00;99.40 | 99.85;99.85 | . | 100.00;100.00 | . | 99.69 | 99.79 | 99.51;99.51;99.51;99.63 | 99.75;99.75;99.75 | 98.19;80.66;97.95 | 99.92;99.61;99.92;99.45;99.84;99.69;99.61;99.92 | 99.83;99.75;99.83;99.83 | 99.75 | 99.52;99.76 | 99.84;99.74 | 99.91;99.91;100.00;100.00;100.00;99.91;99.83;100.00;100.00;99.91;100.00;99.91;99.83;99.74;99.91;100.00;99.91;100.00;99.91;99.83;100.00;100.00;99.91;100.00;99.91;99.74;98.54 | 99.94 |
| `FAL11387` | 53 | . | . | 100.00 | 99.90 | 99.78 | . | . | . | 99.91 | . | 99.88 | . | 99.88 | 99.95 | 99.76 | 100.00 | 99.93 | 99.92 | 99.82 | . | . | 99.78;100.00 | . | 99.68;99.89;99.79 | . | . | . | 99.75 | 99.83 | . | 99.74 | 99.94 | . | 99.67 | 99.84 | . | 99.59 | 99.88 | 100.00 | 99.12 | . | 99.49 | 99.54 | 100.00 | 99.84 | 99.94 | 99.87 | 99.91 | 99.90 | . | . | 99.70 | 99.73 | 100.00 | 99.84 | 99.71 | 99.95 | 99.59 | . | 99.58 | . | 100.00;99.70;100.00;99.70 | . | 99.79 | 99.92 | 99.90 | . | . | . | 99.85 | . | . | 99.69 | . | 99.51 | . | 97.95 | 99.92;99.92 | 99.59 | . | 99.28 | . | 100.00;100.00;100.00;99.91;99.74;100.00;100.00;100.00;100.00;99.74;100.00 | 99.88 |
| `FAL11417` | 56 | . | . | 100.00 | 99.94 | 99.71 | 99.91 | 99.90 | 100.00 | 99.83 | . | 99.75;99.88;99.88 | . | 99.88;99.88 | 99.95;99.95;99.95 | 99.76 | 100.00 | 99.93 | 100.00 | . | . | . | 99.78;99.86 | 100.00 | 99.68;99.89;100.00;99.89;99.68;99.89;99.89;99.58;99.89;99.79 | 100.00 | 99.91;99.91 | 100.00;99.94 | 99.83 | 99.83 | 100.00;100.00 | 99.61 | . | . | 99.67 | 100.00;99.92;99.42;99.92 | 99.53 | 99.59 | 94.67;92.61 | 99.76 | 99.26 | . | 99.49 | 99.54 | 99.92 | 99.84 | 99.97 | 99.84 | 99.91 | 99.94 | . | . | . | . | . | 98.22;99.84 | . | . | . | 99.89 | 99.58 | . | 99.70;99.70;100.00;100.00 | . | 99.84 | 100.00 | 99.90 | . | . | . | . | 100.00 | . | . | . | 99.39;99.63 | . | 97.95 | 99.92;99.92;99.76;99.92 | 99.83;99.75 | 99.83 | 99.76 | 99.74 | 100.00;99.91;100.00;99.91;99.83;100.00;99.91;100.00;99.74;99.91;99.74;99.83;100.00;100.00;100.00;99.83;99.06;100.00;100.00 | 99.82 |
| `FAL11425` | 57 | . | . | 100.00 | 99.94 | 99.71 | 99.74 | 99.87 | 99.70 | 99.83 | . | . | . | . | 99.84 | 99.76 | 100.00 | 99.86 | 100.00 | 99.74 | . | . | 100.00;99.85 | 100.00 | 99.79 | . | 100.00 | 99.94 | 99.83;99.92 | 99.83 | 100.00 | 99.81 | 99.94 | . | 99.83 | . | . | 99.59 | 99.64 | 100.00 | 99.26 | . | 99.49 | 99.77 | 99.84 | 99.84 | 99.94 | 99.90 | 99.91 | 99.90 | 99.67 | 99.92 | 100.00 | 99.73 | 100.00 | 99.92 | 99.81 | 100.00 | 99.66 | . | 99.16 | 99.94 | 100.00;99.70;100.00;99.70 | . | 99.84 | 100.00 | 99.90 | . | . | . | . | 100.00 | . | 100.00 | . | . | . | . | 99.92 | . | 99.23 | . | . | 100.00 | 99.88 |
| `FAL16059` | 67 | . | . | 100.00 | 99.87 | 99.78 | 100.00 | 99.94 | 99.85 | 99.83 | 96.51 | 99.88;99.88;99.88;99.88 | . | 100.00;99.88;99.76;99.88 | 99.78;100.00 | 99.76 | 100.00 | 99.86 | 100.00 | 100.00 | . | . | 100.00;99.93 | 100.00 | 99.89;99.79;99.79;99.79;99.79;99.79;99.89;99.79;99.79;99.79;99.68 | 99.79 | 99.91 | 99.87 | 99.83 | 100.00 | 99.81 | 99.68 | 99.94;100.00 | . | 99.72 | 99.75;99.92 | . | 99.59 | 99.88 | 99.76 | 100.00 | . | 99.49 | 99.54 | 99.84 | 99.84 | 99.94 | 99.81 | 99.91 | 99.84 | 99.67 | 99.92 | 99.70 | 99.45 | 100.00 | 98.13;99.76 | 99.71 | 99.90 | 99.45 | 99.78 | 99.58 | 99.94 | 100.00;99.70 | . | 99.79 | 100.00 | 99.90 | . | . | . | . | 100.00 | . | 99.69 | . | 99.51;98.53;99.51;99.63 | . | 86.56 | 99.92;99.92;99.84 | 99.83;99.83;99.75 | . | 99.76 | 99.58;99.64 | 100.00;100.00;99.91;99.83;100.00;99.83;99.91;100.00;99.91 | 99.88 |
| `FAL17459` | 64 | . | . | 100.00 | 99.90 | 99.81 | 100.00 | 99.90 | 100.00 | 100.00 | 97.92 | 99.88 | . | 99.88 | 99.89;99.89 | 99.76 | 99.86;100.00 | 100.00;99.93 | 100.00 | 99.82 | . | . | 100.00;100.00 | 100.00 | 99.89;99.78 | 99.60 | 99.83 | 99.94 | 99.83 | 100.00;99.83;99.91 | 100.00 | 99.68;99.81;99.61 | 99.88 | . | 99.92;99.89;99.58 | 99.34 | . | 99.73;99.45 | 99.76 | 100.00 | 99.71 | . | 99.49;99.49;99.23 | 99.54;99.54;99.77 | 99.84 | 99.92;99.92 | 99.97;99.97 | 99.84;99.90 | 99.91;99.91 | 98.84;99.94 | 99.84 | 99.92 | 100.00 | 99.45 | 100.00 | 99.84;99.84 | 99.81 | 99.95 | 99.66 | . | 99.58;99.58 | 100.00 | 100.00;99.70;100.00;99.70 | . | 99.84 | . | 99.90 | . | . | . | 99.70 | 100.00;100.00;99.77 | . | 99.38 | . | 99.63 | 99.87 | 97.95;80.37 | 99.69;99.84 | . | . | . | . | 100.00;100.00;100.00;100.00;100.00 | 99.82 |
| `FAO71993` | 69 | . | . | 100.00 | 99.87 | 99.74 | 99.91 | 99.87 | 99.85 | 99.83 | 81.89 | 99.39;99.76;99.39;99.03;99.88 | . | 99.88;99.76;99.88 | 99.89 | 99.76 | 100.00 | 99.93 | 100.00 | 99.82 | . | . | 99.86;99.78 | 100.00 | 100.00;87.29;99.47;99.79 | 98.95;99.58 | 99.83 | 99.94 | 99.75;99.92 | 99.83 | 99.81 | 99.68 | 99.82 | 99.87 | 99.75 | 99.75;99.84 | 92.20 | 99.73 | 99.88 | 99.76 | 99.12 | . | 99.74 | 99.54 | 99.84 | 99.92 | 99.90 | 99.81 | 99.91 | 99.90 | 99.84 | 100.00 | 99.39 | 100.00 | 100.00 | 99.59;99.68 | 99.71 | 99.90 | 99.66 | 100.00 | 99.58 | 100.00 | 100.00;99.70;100.00;99.70 | . | 99.84 | 99.85 | 99.90 | . | . | . | . | 100.00 | . | 100.00 | . | 99.51;99.14;99.26 | . | 86.77;86.77 | 99.92;99.84;99.92;99.92 | 99.75;99.83;99.83 | 99.58 | 99.52;99.76;99.76 | . | 100.00 | 99.88 |
| `FAO72169` | 65 | . | . | 100.00 | 99.84 | 99.71 | 99.91 | 99.94 | 99.85 | 99.91 | . | 99.64;99.52 | . | 99.88;99.64 | 98.63;99.84 | 99.76 | 99.86;100.00 | 99.86;99.86 | 99.92 | 99.91 | . | . | 99.86;99.85 | 100.00 | 99.58;99.79;99.58;99.79 | 99.16 | 99.83 | 99.94 | 99.83 | 99.91;99.83 | 100.00 | 99.81;99.68 | 99.94 | . | 99.81;99.75 | 98.68 | 99.76 | 99.59 | 99.88 | 99.76 | 99.26 | . | 99.49 | 99.54 | 99.92 | 99.84;99.84 | 99.90;99.90 | 99.87;99.90 | 99.91 | 99.81 | 99.67 | 99.92 | 99.70 | 100.00 | 100.00 | 99.92 | 99.81;99.42;99.61;98.16 | 99.90;99.90;97.95 | 99.59;99.52 | . | 99.37 | 100.00 | 99.70;99.70;100.00;100.00 | . | 99.74 | 100.00 | 99.90 | . | . | . | . | 100.00 | . | 100.00 | . | 99.63;99.51 | . | 98.11;97.95 | 99.92 | 99.67;99.67 | . | 99.52;99.76 | . | 100.00;100.00;100.00;99.91;100.00 | 99.94 |
| `FAO96374` | 72 | . | . | 100.00 | 99.90 | 99.71 | 99.91 | 99.94 | 99.85 | 99.91 | 97.56;96.51;99.87 | 99.88;99.88;99.88;99.88;99.88;89.01 | 99.51 | 99.88;99.88;99.88;99.88;99.88;99.88;84.71 | 99.95 | 99.76 | 100.00 | 99.86 | 100.00 | 99.82 | . | . | 99.85;100.00 | 100.00 | 99.58;99.79;99.58;99.79;99.68;95.76;99.89 | 99.37;98.52;100.00;98.31 | 99.91 | 100.00 | 99.83 | 99.91 | 99.62 | 99.81 | 99.94;99.82 | 99.74 | 99.83 | 99.84;99.75;98.60;99.92 | . | 99.73 | 100.00 | 99.76 | 99.26 | . | 99.49 | 99.54 | 99.92 | 99.92 | 99.94 | 99.90 | 99.91 | 99.87 | 99.84 | 99.92 | 99.39 | 99.73 | 100.00 | 100.00 | 99.90 | 99.95 | 99.86 | 100.00 | 99.58 | 99.94 | 99.70 | . | 99.84 | 99.85 | 99.90 | 99.40 | . | . | 98.48;100.00 | 100.00 | . | 100.00 | 99.79 | 99.63;99.51;99.63 | . | 97.63;97.95 | 99.92;99.84;99.92;99.69 | 99.83;99.75 | . | 99.76;100.00 | 99.69 | 99.91;99.83;99.83;99.91;99.49;100.00;100.00;99.91;99.83;99.83;100.00;99.83 | 99.88 |
| `FAO97359` | 72 | . | 100.00 | 100.00 | 99.87 | 99.71 | 99.83 | 99.94 | 99.70 | 99.74 | 96.03;97.80;97.56;99.18;97.80 | 99.88;99.63;99.88 | . | 99.88;99.76 | 99.67 | 99.76 | 100.00 | 99.86 | 100.00 | 99.74 | . | 98.56 | 99.86;99.85 | 99.68 | 99.79;99.79;99.79;99.89 | 98.80;99.00;99.60 | 99.91 | 100.00 | 99.92 | 99.83;99.83 | 100.00 | 99.68;99.74 | 99.82 | . | 99.69;98.72;99.64 | 99.75;99.84 | . | 99.59 | 99.88 | 100.00 | 99.26;99.12 | . | 99.49 | 99.77 | 99.92 | 99.84 | 99.87 | 99.90 | 99.91 | 99.84 | 99.67 | 100.00 | 99.70 | 100.00 | 100.00 | 99.76 | 99.71 | 99.95 | 99.86 | 98.59 | 99.16 | 100.00 | 100.00;99.40 | . | 99.89 | 100.00 | 99.90 | . | 98.80 | . | 99.85 | 100.00 | . | 99.69 | 99.38;99.07 | 99.63;99.63 | 99.62;99.62 | . | 99.53;99.76;99.53;99.84 | 99.83 | 99.49 | 99.52 | . | 99.91;100.00;99.91;100.00;99.91;100.00;99.91 | 99.94 |
| `FAQ33890` | 72 | 100.00 | . | 100.00 | 99.90 | 99.81 | 99.74 | 99.81 | 99.70 | 99.91 | 96.15 | 99.76;99.76;99.88;99.88;99.88 | 99.51;99.51 | 99.88;99.88;100.00;99.88;99.88 | 99.84;99.89;99.62 | 99.76 | 100.00 | 99.86 | 100.00 | 99.65 | . | . | 99.85;100.00 | 99.84 | 97.67;99.89;100.00;99.58;99.79;99.68;99.79 | 100.00 | 100.00 | 99.94 | 99.83 | 99.91;99.83 | 99.62 | 99.81;99.81 | 99.76 | . | 99.75;99.72 | 99.92;99.18;99.92;100.00 | . | 99.59 | 99.76 | 99.76;99.52 | 99.26 | . | 99.74 | 99.54 | 100.00 | 99.84 | 99.87 | 99.84 | 99.91 | 99.87 | 99.67;99.84 | 100.00;100.00 | 99.70 | 99.73 | 100.00 | 99.84 | 99.71;99.81 | 99.85;99.95 | 99.59;99.73 | . | 99.58 | 100.00 | 99.40;100.00 | . | 99.68 | 99.92 | 99.90 | 100.00 | . | . | 99.85 | 100.00 | . | 100.00 | . | 99.51;99.63;99.63 | 99.62 | 98.03 | 99.22;99.76;98.90;97.65;99.92;99.92 | 99.75;99.67 | 99.91 | 99.52;99.52 | 99.58 | 99.91;99.91;99.91;99.91;100.00;99.83;99.83;99.91;99.83;99.91;99.91;99.91 | 99.94 |
| `FAQ33923` | 77 | 99.87 | . | 100.00 | 99.87 | 99.65 | 99.74 | 99.77 | 99.55 | 99.65 | 98.86;96.39;97.68;96.03 | 99.88;99.76;99.88;99.64 | 99.51;99.51 | 99.88;99.88;99.88;100.00 | 99.84 | 99.51 | 100.00 | 99.79 | 100.00 | 99.47 | . | 99.66 | 99.86;99.78 | 99.84 | 99.78;99.58;99.79;99.78;99.68;99.47;99.68;99.68;99.47;99.79;99.68 | 98.31;99.58 | 99.66 | 99.94 | 99.92 | 98.54;99.57;99.91;99.74;99.40;99.83;99.48;99.48;99.83 | 99.44 | 99.03;99.74;99.61;99.55;99.35;99.74;99.55;99.68;99.68;99.74 | 99.76 | . | 99.72 | 99.92;99.67 | 89.83 | 99.73 | 100.00 | 99.76 | 99.12;99.26 | 98.89 | 99.74 | 99.54 | 99.76 | 99.68 | 99.81 | 99.81 | 99.83 | 99.78 | 99.51 | 100.00 | 99.70 | 99.73 | 100.00 | 99.84 | 99.81 | 99.90 | 99.59 | 99.67 | 99.16 | 99.89 | 100.00;100.00;99.40 | . | 99.84 | 99.92 | . | 100.00 | 100.00 | 99.85 | . | 100.00 | . | 99.38 | 99.59;99.90 | 99.51;99.51;99.63;99.51 | 99.62 | 98.03;98.03 | 99.92;99.76;99.61;98.98 | 99.67;99.83 | 99.83 | 99.76;99.76 | 99.38;98.59 | 100.00 | 99.88 |
| `FAQ34034` | 68 | 99.36 | . | 100.00 | 99.87 | 99.78 | 99.83 | 99.87 | 99.70 | 99.83 | 96.27 | 99.76;99.88;99.88;99.88 | 99.26 | 99.88;99.76;99.76;99.76 | 99.95;99.89;99.84 | 99.76 | 100.00;83.82 | 99.93;99.93 | 99.83 | 99.82 | . | . | 100.00;99.78 | 100.00 | 99.89 | 99.79 | 99.74;99.83;99.91 | 99.94;100.00;99.94 | 100.00 | 99.83 | 100.00;100.00;100.00 | 99.74 | 100.00 | . | 99.75 | 100.00;99.92 | . | 99.59 | 99.76 | 99.76 | 99.26 | . | 99.49 | 99.54 | 99.84 | 99.84 | 99.90 | 99.90;99.81 | 99.91 | 99.87 | 99.76 | 100.00 | 99.39 | 100.00 | 100.00 | 99.92 | 99.71 | 99.95 | 99.93 | . | 99.37 | 100.00 | 99.70;99.70;100.00 | . | 99.89 | 100.00 | 99.90 | 100.00 | . | . | . | 100.00 | . | 100.00 | . | 99.51;99.51;99.63;99.63 | . | 98.11;98.11 | 99.92;99.92;99.92;99.92 | 99.75 | . | 99.76;99.52 | . | 100.00;98.46;100.00;99.91;99.91;99.83;100.00;100.00 | 99.88 |

---

## ABRICATE\_RESFINDER

The results table shown here is a collection from all samples.

| #FILE | NUM\_FOUND | aac(2')-IIa\_1 | aac(3)-VIa\_2 | aadA1\_2 | aadA2\_1 | aadA5\_1 | ant(3'')-Ia\_1 | aph(3'')-Ib\_2 | aph(3'')-Ib\_3 | aph(3'')-Ib\_5 | aph(3')-IIa\_2 | aph(3')-Ia\_1 | aph(3')-Ia\_10 | aph(3')-Ia\_3 | aph(3')-Ia\_7 | aph(3')-Ia\_9 | aph(6)-Id\_1 | blaCTX-M-15\_1 | blaCTX-M-55\_1 | blaSHV-161\_1 | blaSHV-1\_1 | blaTEM-122\_1 | blaTEM-141\_1 | blaTEM-156\_1 | blaTEM-1A\_1 | blaTEM-1B\_1 | blaTEM-1C\_1 | catA1\_1 | cmlA1\_1 | cmlA1\_2 | dfrA12\_8 | dfrA14\_1 | dfrA14\_5 | dfrA17\_1 | dfrA1\_8 | dfrA5\_1 | erm(B)\_18 | floR\_2 | fosA7\_1 | mph(A)\_2 | mph(B)\_1 | qacE\_1 | qnrB19\_1 | qnrS1\_1 | qnrS2\_1 | qnrS4\_1 | sitABCD\_1 | sul1\_5 | sul2\_11 | sul2\_2 | sul2\_3 | sul3\_2 | tet(A)\_6 | tet(B)\_1 | tet(B)\_2 | tet(C)\_2 | tet(C)\_3 | tet(M)\_5 | tet(M)\_8 |
| --- | --- | --- | --- | --- | --- | --- | --- | --- | --- | --- | --- | --- | --- | --- | --- | --- | --- | --- | --- | --- | --- | --- | --- | --- | --- | --- | --- | --- | --- | --- | --- | --- | --- | --- | --- | --- | --- | --- | --- | --- | --- | --- | --- | --- | --- | --- | --- | --- | --- | --- | --- | --- | --- | --- | --- | --- | --- | --- | --- |
| `FAL00958` | 5 | . | . | . | . | . | . | . | . | . | . | . | . | . | . | . | 99.88 | . | . | . | . | . | . | . | . | . | . | . | . | . | . | . | 99.17 | . | . | . | . | . | . | . | . | . | . | . | . | . | 99.45;97.98 | . | 99.26 | . | . | . | 97.65 | . | . | . | . | . | . |
| `FAL01198` | 33 | 99.62 | . | . | 97.80 | . | 82.41 | 99.75;99.75;99.75 | . | 99.88;99.88 | . | . | . | . | . | 99.51 | 99.88;99.88;99.88;99.88;99.88 | . | 100.00 | 99.88 | . | 99.77;99.88 | . | 99.65;99.65;99.65 | . | . | . | 99.55 | 99.29 | . | 99.60 | . | 98.96 | . | . | 99.79 | 99.87 | 99.75;99.84;99.75 | 100.00 | 100.00 | . | 85.59 | . | 100.00 | . | . | 99.51;99.34;99.51 | 100.00 | 99.51 | 99.63;99.63;99.63 | . | 99.87 | 99.92;97.65;97.65;97.73;99.84;97.49 | 99.83;99.83 | 99.92;99.83;99.83 | 99.91 | 99.83 | 99.79 | . |
| `FAL01556` | 19 | . | . | . | . | 99.87 | . | . | . | 99.88;99.88;99.75 | . | . | . | . | . | . | 99.88;99.88;99.52 | 99.89 | . | . | . | 99.77 | . | . | 99.77 | 99.88 | . | . | . | . | . | . | . | 98.95 | . | . | . | 99.75;99.67;99.75;99.75;99.67 | . | . | 98.68 | 85.59 | . | 100.00;100.00 | . | . | 99.60 | 99.88 | 99.63 | 99.63;99.75 | . | . | 99.92;97.73;99.92;97.73;97.73;97.73;94.27;97.41 | 99.92 | 99.83 | . | . | . | . |
| `FAL05306` | 25 | . | . | 99.24 | 96.70;93.28 | 99.62 | 82.20 | 99.75 | . | . | . | . | . | . | . | . | 99.88 | . | . | . | . | . | 99.54 | . | . | . | . | . | . | 99.29 | 96.99;96.18 | . | . | 99.58 | 99.58 | . | 98.82 | 99.75;99.84 | . | 99.78 | . | 85.59 | . | 99.85;99.85 | . | . | 99.22;99.16 | 99.77;99.77 | 99.51 | . | . | 98.86 | 99.92 | 99.83 | . | . | 99.75 | 99.48 | 98.54 |
| `FAL11145` | 12 | . | . | . | . | . | 82.10 | 99.38 | . | 99.88;99.75;99.00;99.88 | . | 99.14 | . | . | . | . | 99.88;99.88;100.00;99.40;99.28 | . | . | . | . | . | . | 99.65 | . | . | . | . | . | . | . | . | . | . | 98.10 | . | . | 99.67;99.42;99.09;99.67 | . | . | . | . | . | . | . | . | 99.13 | . | 99.75;99.63;99.26;99.63 | . | . | . | 99.84;99.69;99.53;99.61 | 99.83 | . | . | . | . | . |
| `FAL11151` | 9 | . | . | . | . | . | . | . | 99.25 | 99.50 | . | . | . | . | . | . | 99.88;99.76 | . | . | . | . | . | . | . | . | 99.54 | . | . | . | . | . | . | . | . | . | . | . | . | . | . | . | . | . | . | . | . | 99.16 | . | 98.77 | 99.51 | . | . | . | . | 99.92 | 99.49 | . | . | . |
| `FAL11305` | 16 | . | . | . | . | . | 82.30 | 99.75 | . | 99.88 | . | 98.16;99.63 | . | . | . | . | 99.88;99.88 | 99.77 | . | . | . | . | . | . | 100.00 | . | . | 97.12 | . | . | . | . | . | . | 100.00 | . | . | 99.92;99.84 | . | . | 100.00 | . | . | . | . | . | 92.89;98.41 | . | 99.63;99.63 | . | . | . | 99.84;99.84 | . | 99.83;99.83 | . | . | 99.38 | . |
| `FAL11351` | 19 | . | . | . | . | . | 82.30 | . | . | 99.88 | . | . | . | . | 99.88 | . | 84.95;99.88 | . | . | . | 99.77 | . | . | . | . | 99.88 | 99.77 | . | . | . | . | . | . | . | 99.79 | . | 99.61 | 99.84 | 100.00;100.00 | . | . | 85.59 | . | . | . | 100.00 | 99.36;99.54;99.36;99.22 | 99.88 | . | . | . | 100.00 | 97.65;99.84 | 99.67 | . | 99.83 | . | . | . |
| `FAL11384` | 29 | 98.34 | . | . | 97.68 | . | 82.41;82.20;82.10 | 99.75;99.63;99.75;99.75 | . | 99.88;99.88;99.88 | 99.62 | 99.75 | . | . | . | 99.88 | 99.88;99.64;100.00;99.88;99.88;99.88;99.88 | . | . | . | . | . | . | 99.77;99.65 | 99.88 | . | . | . | 99.76 | . | . | . | . | 98.95 | 99.79 | 99.16 | . | 99.75;99.92;99.75;99.67 | 99.76 | . | 99.67 | 85.59 | 99.84;99.84 | . | . | . | 99.39;99.36;99.54 | 99.88 | 99.51;99.51;99.51 | 99.63 | . | 99.75;99.75;99.75 | 97.73;97.41;99.92;97.25;99.84;97.49;99.61;97.73 | . | 99.83;99.75;99.83;99.83 | . | 99.75 | 99.84;99.74 | . |
| `FAL11387` | 9 | . | . | . | . | . | . | . | . | 99.88 | . | . | . | . | . | . | 99.88 | . | . | . | . | . | . | 99.65 | . | . | . | . | . | . | . | . | . | . | . | . | . | 99.75 | . | . | . | . | . | 99.85 | . | . | 99.22 | . | 99.51 | . | . | . | 97.73;99.92 | 99.59 | . | . | . | . | . |
| `FAL11417` | 17 | . | . | . | . | . | . | 99.75 | . | 99.88 | . | . | . | 99.75 | . | . | 99.88;99.88 | . | . | . | . | . | . | 99.65 | . | . | . | . | . | . | . | . | . | . | 100.00 | . | . | 99.92;99.84;99.34;99.84 | 99.53 | 99.89 | . | . | . | . | . | . | 99.60;99.42 | . | 99.39 | 99.63 | . | . | 99.92;99.92;99.76;97.73 | 99.75 | 99.83 | 99.83 | . | 99.74 | . |
| `FAL11425` | 3 | . | . | . | . | . | . | . | . | . | . | . | . | . | . | . | . | . | . | . | . | . | . | . | . | . | . | . | . | . | . | . | . | . | . | . | . | . | . | . | . | . | . | . | . | . | 99.25;99.25 | . | . | . | . | . | 97.73 | . | . | 99.23 | . | . | . |
| `FAL16059` | 15 | . | . | . | . | . | 82.51 | 99.75;99.75;99.75 | . | 99.88 | . | . | . | . | . | . | 100.00;99.88;99.76;99.88 | . | . | . | . | . | . | 99.77 | . | . | . | . | . | . | . | . | . | . | 99.79 | . | . | 99.67;99.84 | . | 99.78 | . | . | . | . | . | . | 99.34;99.31;99.31 | . | 99.51;98.53;99.51 | 99.63 | . | . | 97.73;99.92;99.84 | 99.83;99.83 | 99.75 | . | . | 99.58;99.64 | . |
| `FAL17459` | 11 | . | . | . | 97.92 | . | . | . | . | 99.88 | . | . | . | . | . | . | 99.88 | . | . | . | . | . | . | 99.65;99.88 | . | . | . | . | . | . | 99.60 | . | . | . | . | . | . | 99.26 | . | . | . | . | . | 99.70 | . | . | 99.45 | . | . | 99.63 | . | 99.87 | 99.69;97.65 | . | . | . | . | . | . |
| `FAO71993` | 17 | . | . | . | . | . | 81.89 | 99.63 | . | 99.00;99.88 | . | . | . | 99.39;99.39 | . | . | 99.88;99.76;99.88 | . | . | . | . | . | . | . | . | 100.00;100.00 | . | . | . | . | . | . | 99.17 | . | 99.58 | . | 99.87 | 99.67;99.75 | 92.20 | 100.00 | . | . | . | . | . | . | 92.17;99.16;98.01 | . | 99.51;99.14;99.26 | . | . | . | 97.73;99.84;97.73;97.73 | 99.75;99.83;99.83 | . | . | 99.58 | . | . |
| `FAO72169` | 12 | . | . | . | . | . | . | 99.38 | . | 99.63 | . | . | . | . | . | . | 99.88;99.64 | . | . | . | . | . | . | . | . | 99.88;99.65 | . | . | . | . | . | . | . | . | . | 99.16 | . | 98.68 | 99.76 | . | . | . | . | . | . | . | 99.45;98.03;99.51 | . | 99.51 | . | 99.63 | . | 99.92 | . | 99.67;99.67 | . | . | . | . |
| `FAO96374` | 25 | . | . | . | 97.56 | 99.87 | 82.51 | . | . | 99.88;99.88;99.88;99.88;99.88;91.67 | . | 99.51 | . | . | . | . | 99.88;99.88;99.88;99.88;99.88;99.88;84.71 | . | . | . | . | . | . | 99.77 | . | 99.42 | . | . | . | . | . | 99.37 | 98.14 | 98.31 | 100.00 | . | 99.74 | 99.75;99.67;98.60;99.84 | . | 100.00 | . | 85.59 | . | 98.48 | 100.00 | . | 99.28 | 99.88 | 99.63;99.51 | 99.63 | . | . | 99.92;97.65;99.92;97.49 | 99.83;99.75 | . | . | . | 99.69 | . |
| `FAO97359` | 20 | . | 100.00 | . | 97.80;97.56;97.80 | . | 82.10;99.18 | 99.75 | . | 99.88 | . | . | 99.63 | . | . | . | 99.88;99.76 | . | . | . | . | . | . | . | . | . | . | . | 98.41 | . | 98.80;99.00;99.60 | . | . | . | . | . | . | 99.67;99.75 | . | 98.59 | . | 85.29;85.59 | . | 99.85 | . | . | 97.89;99.34 | 99.31;99.77 | 99.63;99.63 | . | . | 99.62;99.62 | 97.33;99.76;97.33;99.84 | 99.83 | . | 99.49 | . | . | . |
| `FAQ33890` | 17 | 100.00 | . | . | . | . | 82.20 | 99.63 | . | 99.75;99.88;99.88;99.88 | . | 99.51;99.51 | . | . | . | . | 99.88;99.88;100.00;99.88;99.88 | . | . | . | . | . | . | . | . | 99.77 | . | . | . | . | . | . | . | . | 100.00 | . | . | 99.84;99.09;99.84;99.92 | . | . | . | . | . | 99.85 | . | . | 99.31;99.34 | . | 99.51;99.63;99.63 | . | . | 99.62 | 99.22;99.76;98.90;97.65;97.73;99.92 | 99.75;99.67 | . | 99.91 | . | 99.58 | . |
| `FAQ33923` | 28 | 99.87 | . | . | 97.68 | 98.86 | 82.41;82.10 | 99.75 | . | 99.75;99.88;99.63 | . | 99.51;99.51 | . | . | . | . | 99.88;99.88;99.88;100.00 | . | . | . | . | . | . | 99.77;95.01 | . | 99.77 | . | . | 99.60 | . | . | . | . | 98.31 | 99.58 | . | . | 99.84;99.59 | 89.83 | 99.67 | . | 85.59 | 99.84 | . | . | . | 97.28 | 99.54;99.88 | 99.51;99.51;99.63;99.51 | . | . | 99.62 | 99.92;99.76;97.41;96.78 | 99.67 | 99.83 | 99.83 | . | 99.38 | 98.59 |
| `FAQ34034` | 15 | 99.36 | . | . | . | . | 82.30 | 99.75 | . | 99.75;99.88;99.88 | . | 99.26 | . | . | . | . | 99.88;99.76;99.76;99.76 | . | . | . | . | . | . | 99.88 | 99.88 | . | . | . | . | . | . | . | . | . | 99.79 | . | . | 99.92;99.84 | . | . | . | . | . | . | . | . | 99.36 | . | 99.51;99.51;99.63 | 99.63 | . | . | 97.73;99.92;97.73;97.73 | . | 99.75 | . | . | . | . |

---

## ABRICATE\_ARGANNOT

The results table shown here is a collection from all samples.

| #FILE | NUM\_FOUND | (AGly)aac(2')-IIa\_Bg | (AGly)aac3-VIa | (AGly)aadA1-pm | (AGly)aadA2 | (AGly)aadA22 | (AGly)aadA3 | (AGly)aadA5 | (AGly)aph(3'')-Ia | (AGly)aph3-Ia | (AGly)aphA2 | (AGly)sat-2A | (AGly)strA | (AGly)strB | (Bla)AmpC1\_Ecoli | (Bla)AmpC2\_Ecoli | (Bla)Penicillin\_Binding\_Protein\_Ecoli | (Bla)ampH | (Bla)ampH\_Ecoli | (Bla)blaCTX-M-15 | (Bla)blaCTX-M-55 | (Bla)blaSHV-1 | (Bla)blaTEM-105 | (Bla)blaTEM-122 | (Bla)blaTEM-141 | (Bla)blaTEM-150 | (Bla)blaTEM-156 | (Fcyn)FosA7 | (Flq)qnr-S1 | (Flq)qnrB5 | (Flq)qnrS | (Flq)qnrS2 | (MLS)erm(B) | (MLS)mph(A) | (MLS)mph(B) | (Phe)catA1 | (Phe)cmlA1 | (Phe)cmlA4 | (Phe)floR | (Sul)sul1 | (Sul)sul2 | (Sul)sul3 | (Tet)tetA | (Tet)tetB | (Tet)tetC | (Tet)tetM | (Tet)tetR | (Tmt)dfrA1 | (Tmt)dfrA12 | (Tmt)dfrA14 | (Tmt)dfrA17 | (Tmt)dfrA5 |
| --- | --- | --- | --- | --- | --- | --- | --- | --- | --- | --- | --- | --- | --- | --- | --- | --- | --- | --- | --- | --- | --- | --- | --- | --- | --- | --- | --- | --- | --- | --- | --- | --- | --- | --- | --- | --- | --- | --- | --- | --- | --- | --- | --- | --- | --- | --- | --- | --- | --- | --- | --- | --- |
| `FAL00958` | 9 | . | . | . | . | . | . | . | . | . | . | . | . | 99.88 | 99.77 | 100.00 | 99.84 | . | 99.65 | . | . | . | . | . | . | . | . | . | . | . | . | . | . | . | . | . | . | . | . | . | 99.26 | . | 99.84 | . | . | . | 99.69 | . | . | 99.16 | . | . |
| `FAL01198` | 32 | 99.62 | . | 96.39 | 99.87 | . | . | . | . | 99.26 | . | . | 99.88;99.88;99.88;99.88;99.88 | 99.88;99.88;99.88;99.88;99.88 | 100.00 | 100.00 | 99.79 | . | 99.91 | . | 100.00 | 99.77 | . | 99.77;99.88 | . | . | 99.65;99.65;99.65 | 100.00 | 100.00 | . | . | . | 99.86 | 100.00 | . | 99.55 | 99.29 | . | 99.84;99.92;99.84 | 100.00 | 99.63;99.63;99.51;99.63 | 99.87 | 97.73;99.84;99.84;99.92;97.65;99.69 | 99.92;99.83;99.83;99.83;99.83 | 97.31;99.83 | 99.79 | 99.85;80.18;99.85;100.00;100.00;100.00;99.54 | . | 99.60 | 98.95 | . | 99.79 |
| `FAL01556` | 20 | . | . | . | . | . | . | 99.87 | . | . | . | . | 99.88;99.88;99.75 | 99.88;99.88;99.52 | 99.92 | 99.91 | 99.89 | . | 100.00 | 99.89 | . | . | 99.88 | 99.77 | . | 99.77 | . | . | 100.00;100.00 | . | . | . | . | . | 98.68 | . | . | . | 99.84;99.75;99.84;99.84;99.75 | 99.88 | 99.63;99.63;99.75 | . | 97.73;99.92;97.73;99.92;99.92;99.92;94.27;99.61 | 99.92;99.83 | . | . | 100.00;100.00;99.69;99.69;99.85;99.69;100.00;100.00;100.00;99.54 | . | . | . | 98.95 | . |
| `FAL05306` | 28 | . | . | 96.15 | 98.72 | 99.24 | 95.20 | 99.62 | . | . | . | 99.81 | 99.88 | 99.88 | . | 99.82 | 99.79 | . | 99.91 | . | . | . | . | . | 99.54 | . | . | . | 99.85;99.85 | . | . | . | 98.92 | 99.78 | . | . | . | 99.21 | 99.84;99.92 | 99.76;99.76 | 99.51 | 98.86 | 97.73 | 99.83 | 99.75 | 98.59;99.48 | 100.00 | 99.58 | 96.99;96.18 | . | 99.58 | . |
| `FAL11145` | 16 | . | . | 96.03 | . | . | . | . | . | 99.14 | . | 98.10 | 99.88;99.50;99.75;99.00;99.88 | 99.88;99.88;100.00;99.40;99.28 | 99.92 | 99.74 | 99.63 | . | 99.83 | . | . | . | . | . | . | . | 99.65 | . | . | . | . | . | . | . | . | . | . | . | 99.75;99.51;99.18;99.75 | . | 99.75;99.63;99.26;99.63 | . | 97.65;97.49;97.33;97.41 | 99.83 | . | . | 99.69;99.54;90.32;99.54;99.54 | 98.10 | . | . | . | . |
| `FAL11151` | 10 | . | . | . | . | . | . | . | . | . | . | . | 99.50;99.25 | 99.88;99.76 | 100.00 | 99.82 | 99.79 | . | 99.83 | . | . | . | 99.54 | . | . | . | . | . | . | . | . | . | . | . | . | . | . | . | . | . | 99.51;98.77 | . | . | 99.92 | 96.89 | . | . | . | . | . | . | . |
| `FAL11305` | 20 | . | . | 96.27 | . | . | . | . | . | 98.16;99.63 | . | 99.62 | 99.88;99.88 | 99.88;99.88 | 100.00 | 100.00 | 99.79 | . | 99.83 | 99.77 | . | . | . | . | . | 100.00 | . | . | . | . | . | . | . | . | 100.00 | 97.12 | . | . | 100.00;99.92 | . | 99.63;99.63 | . | 97.65;97.65 | 99.83;99.83 | . | 99.38 | 100.00;99.85 | 100.00 | . | . | . | . |
| `FAL11351` | 24 | . | . | 96.27 | . | . | . | . | 99.88 | . | . | 100.00 | 99.88 | 84.95;99.88 | 100.00 | 99.91 | 99.89 | 99.48 | 99.74 | . | . | 99.65 | 99.88 | 99.77 | . | . | . | 100.00;100.00 | . | . | 100.00 | . | 99.59 | . | . | . | . | . | 99.92 | 99.88 | . | 100.00 | 99.84;97.65 | 99.67 | 97.23 | . | 100.00;100.00 | 99.79 | . | . | . | . |
| `FAL11384` | 30 | 98.34 | . | 96.39;96.15;96.03 | 99.74 | . | . | . | . | 99.75;99.63 | 99.62 | 100.00 | 99.88;99.75;99.88;99.88;99.88;99.88;99.88 | 99.88;99.64;100.00;99.88;99.88;99.88;99.88 | 100.00 | 99.91 | 99.84 | . | 100.00 | . | . | . | . | . | . | 99.88 | 99.77;99.65 | 99.76 | . | 99.85;99.85 | . | . | . | . | 99.67 | . | 99.76 | . | 99.84;100.00;99.84;99.75 | 99.88 | 99.51;99.51;99.51;99.63 | 99.75;99.75;99.75 | 99.92;99.61;97.73;99.45;97.65;99.69;97.41;99.92 | 99.83;99.75;99.83;99.83 | 99.75 | 99.84;99.74 | 100.00;100.00;99.85;99.69;100.00;100.00;100.00;100.00;99.85 | 99.79 | . | . | 98.95 | 99.16 |
| `FAL11387` | 13 | . | . | . | . | . | . | . | . | . | . | . | 99.88 | 99.88 | 99.92 | 99.82 | 99.79 | . | 99.91 | . | . | . | . | . | . | . | 99.65 | . | 99.85 | . | . | . | . | . | . | . | . | . | 99.84 | . | 99.51 | . | 99.92;97.73 | 99.59 | . | . | 99.85;100.00 | . | . | . | . | . |
| `FAL11417` | 18 | . | . | . | . | . | . | . | . | 99.51 | . | 99.81 | 99.88;99.88 | 99.88;99.88 | 100.00 | . | 99.84 | . | 99.83 | . | . | . | . | . | . | . | 99.65 | 99.53 | . | . | . | . | . | 99.89 | . | . | . | . | 100.00;99.92;99.42;99.92 | . | 99.39;99.63 | . | 97.73;97.73;97.57;99.92 | 99.83;99.75 | 97.23 | 99.74 | 100.00;100.00;100.00;100.00 | 100.00 | . | . | . | . |
| `FAL11425` | 7 | . | . | . | . | . | . | . | . | . | . | . | . | . | 100.00 | 99.74 | 99.84 | . | 99.83 | . | . | . | . | . | . | . | . | . | . | . | . | . | . | . | . | . | . | . | . | . | . | . | 99.92 | . | 98.07 | . | 99.69 | . | . | . | . | . |
| `FAL16059` | 17 | . | . | 96.51 | . | . | . | . | . | . | . | 100.00 | 99.88;99.88;99.88;99.88 | 100.00;99.88;99.76;99.88 | 100.00 | 100.00 | 99.79 | . | 99.83 | . | . | . | . | . | . | . | 99.77 | . | . | . | . | . | . | 99.78 | . | . | . | . | 99.75;99.92 | . | 99.51;98.53;99.51;99.63 | . | 99.92;97.73;97.65 | 99.83;99.83;99.75 | . | 99.58;99.64 | 100.00;99.54;100.00 | 99.79 | . | . | . | . |
| `FAL17459` | 14 | . | . | . | 100.00 | . | . | . | . | . | . | . | 99.88 | 99.88 | . | 99.82 | 99.84 | . | 100.00 | . | . | . | . | . | . | . | 99.65;99.88 | . | 99.70 | . | . | . | . | . | . | . | . | . | 99.34 | . | 99.63 | 99.87 | 97.49;99.84 | . | . | . | 100.00;99.85;99.39;99.85 | . | 99.60 | . | . | . |
| `FAO71993` | 22 | . | . | 95.79 | . | . | . | . | 99.39 | 99.63 | . | 100.00 | 99.75;99.00;99.88 | 99.88;99.76;99.88 | 99.85 | 99.82 | 99.84 | . | 99.83 | . | . | . | 100.00;100.00 | . | . | . | . | 92.20 | . | . | . | . | 99.86 | 100.00 | . | . | . | . | 99.75;99.84 | . | 99.51;99.14;99.26 | . | 99.92;97.65;99.92;99.92 | 99.75;99.83;99.83 | 99.58 | . | 99.69;99.85;100.00;99.54 | 99.58 | . | 98.95 | . | . |
| `FAO72169` | 14 | . | . | . | . | . | . | . | . | . | . | . | 99.63;99.50 | 99.88;99.64 | 100.00 | 99.91 | 99.74 | . | 99.91 | . | . | . | 99.88;99.65 | . | . | . | . | 99.76 | . | . | . | . | . | . | . | . | . | . | 98.68 | . | 99.63;99.51 | . | 97.73 | 99.67;99.67 | . | . | 99.54 | . | . | . | . | 99.16 |
| `FAO96374` | 27 | . | . | 96.51 | 99.74 | . | . | 99.87 | . | 99.51 | . | 100.00 | 99.88;99.88;99.88;99.88;99.88;91.67 | 99.88;99.88;99.88;99.88;99.88;99.88;84.71 | 99.85 | 99.82 | 99.84 | . | 99.91 | . | . | . | 99.42 | . | . | . | 99.77 | . | 98.48 | . | . | 100.00 | 99.73 | 100.00 | . | . | . | . | 99.84;99.75;98.60;99.92 | 99.88 | 99.63;99.51;99.63 | . | 97.73;99.84;97.73;99.69 | 99.83;99.75 | . | 99.69 | 99.85;99.69;99.85;99.85 | 100.00 | . | 99.37;98.52 | 98.31 | . |
| `FAO97359` | 22 | . | 100.00 | 96.03;97.59 | 99.87;99.62;99.87 | . | . | . | . | 99.63 | . | . | 99.88;99.88 | 99.88;99.76 | 100.00 | 99.74 | 99.89 | . | 99.74 | . | . | . | . | . | . | . | . | . | 99.85 | . | . | . | . | 98.57 | . | . | 98.41 | . | 99.75;99.84 | 99.29;99.76 | 99.63;99.63 | 99.62;99.62 | 99.53;97.57;99.53;97.65 | 99.83 | 96.89 | . | 99.85;100.00;99.85;99.69 | . | 98.80;99.00;99.60 | . | . | . |
| `FAQ33890` | 21 | 100.00 | . | 96.15 | . | . | . | . | . | 99.51;99.51 | . | 100.00 | 99.75;99.75;99.88;99.88;99.88 | 99.88;99.88;100.00;99.88;99.88 | 99.92 | 99.65 | 99.68 | . | 99.91 | . | . | . | 99.77 | . | . | . | . | . | 99.85 | . | . | . | . | . | . | . | . | . | 99.92;99.18;99.92;100.00 | . | 99.51;99.63;99.63 | 99.62 | 97.02;97.57;97.65;97.65;99.92;97.73 | 99.75;99.67 | 97.31 | 99.58 | 99.69;99.85;99.69;99.69;99.54;99.85 | 100.00 | . | . | . | . |
| `FAQ33923` | 29 | 99.87 | . | 96.39;96.03 | 99.74 | . | . | 98.86 | . | 99.51;99.51 | . | 99.81 | 99.88;99.75;99.88;99.63 | 99.88;99.88;99.88;100.00 | 99.92 | 99.47 | 99.84 | . | 99.65 | . | . | . | 99.77 | . | . | . | 99.77;95.01 | 89.83 | . | 99.85 | . | . | . | 99.67 | . | . | 99.60 | . | 99.92;99.67 | 99.52;99.88 | 99.51;99.51;99.63;99.51 | 99.62 | 97.73;97.57;99.61;98.98 | 99.67;99.83 | 97.23 | 99.38;98.59 | 99.69;99.54;99.69;99.54 | 99.58 | . | . | 98.31 | . |
| `FAQ34034` | 18 | 99.36 | . | 96.27 | . | . | . | . | . | 99.26 | . | 100.00 | 99.75;99.88;99.88;99.88 | 99.88;99.76;99.76;99.76 | 100.00 | 99.82 | 99.89 | . | 99.83 | . | . | . | . | . | . | 99.88 | 99.88 | . | . | . | . | . | . | . | . | . | . | . | 100.00;99.92 | . | 99.51;99.51;99.63;99.63 | . | 99.92;97.73;99.92;99.92 | 99.75 | . | . | 99.69;99.85;99.69;100.00 | 99.79 | . | . | . | . |

---

## CPIPES Software Versions

Collected at run time from the software output (STDOUT/STDERR).

| Process Name | Software | Version |
| --- | --- | --- |
| `ABRICATE_RUN` | `abricate` | `1.0.1` |
|  | `bash` | `4.2.46(2)-release` |
| `ABRICATE_SUMMARY` | `abricate` | `1.0.1` |
|  | `bash` | `4.2.46(2)-release` |
|  | `grep` | `2.20` |
|  | `sed` | `4.2.2` |
|  | `sort` | `8.22` |
| `CAT_FASTQ` | `cat` | `8.22` |
|  | `gzip` | `1.5` |
| `CENTRIFUGE_CLASSIFY` | `centrifuge` | `1.0.4` |
| `CENTRIFUGE_PROCESS` | `biopython` | `1.79` |
|  | `numpy` | `1.20.3` |
|  | `pandas` | `1.2.5` |
|  | `python` | `3.8.1` |
| `CPIPES` | `CPIPES` | `0.4.0` |
|  | `Nextflow` | `21.12.1.edge` |
|  | `centriflaken` | `0.2.1` |
| `DUMP_SOFTWARE_VERSIONS` | `python` | `3.8.1` |
|  | `yaml` | `5.3` |
| `FASTQC` | `fastqc` | `0.11.9` |
| `FLYE_ASSEMBLE` | `flye` | `2.8-b1674` |
|  | `grep` | `2.20` |
| `GEN_SAMPLESHEET` | `python` | `3.8.1` |
| `KRAKEN2_CLASSIFY` | `gzip` | `1.5` |
|  | `kraken2` | `2.1.2` |
| `KRAKEN2_EXTRACT_CONTIGS` | `biopython` | `1.79` |
|  | `numpy` | `1.20.3` |
|  | `pandas` | `1.2.5` |
|  | `python` | `3.8.1` |
| `MLST` | `mlst` | `2.11` |
| `SAMPLESHEET_CHECK` | `python` | `3.8.1` |
| `SEQKIT_GREP` | `seqkit` | `2.2.0` |
| `SEQKIT_SEQ` | `seqkit` | `2.2.0` |
| `SEROTYPEFINDER` | `head` | `8.22` |
|  | `sed` | `4.2.2` |
|  | `serotypefinder` | `2.0.1/2.0.2` |
|  | `tail` | `8.22` |
| `TABLE_SUMMARY` | `bash` | `4.2.46(2)-release` |
|  | `head` | `8.22` |
|  | `python` | `3.8.1` |
|  | `tail` | `8.22` |

**MultiQC v1.12**
- Written by Phil Ewels,
available on GitHub.

This report uses HighCharts,
jQuery,
jQuery UI,
Bootstrap,
FileSaver.js and
clipboard.js.

×

### Plot Table Data

Select Column

Select Column

Please select two table columns.

Close

×

### Regex Help

Toolbox search strings can behave as regular expressions (regexes). Click a button below to see an example of it in action. Try modifying them yourself in the text box.

`^` (start of string)
`$` (end of string)
`[]` (character choice)
`\d` (shorthand for `[0-9]`)
`\w` (shorthand for `[0-9a-zA-Z_]`)
`.` (any character)
`\.` (literal full stop)
`()` `|` (group / separator)
`*` (prev char 0 or more)
`+` (prev char 1 or more)
`?` (prev char 0 or 1)
`{}` (char num times)
`{,}` (count range)

```
samp_1
samp_1_edited
samp_2
samp_2_edited
samp_3
samp_3_edited
prepended_samp_1
tmp_samp_1_edited
tmpp_samp_1_edited
tmppp_samp_1_edited
#samp_1_edited.tmp
samp_11
samp_11111
```

See regex101.com for a more heavy duty testing suite.

Close
