# Supplementary material for: Centriflaken: An automated data analysis pipeline for assembly and in silico analyses of foodborne pathogens from metagenomic samples
Source: PLoS One. 2025 Dec 23;20(12):e0329425. doi: 10.1371/journal.pone.0329425 (PMC12725755; doi:10.1371/journal.pone.0329425)
Supplement: Attachment — Submitted filename: Response to Reviewers PONE-D-25-38731.docx [file pone.0329425.s009.docx]

**Response to Reviewers PONE-D-25-38731**

centriflaken: an automated data analysis pipeline for assembly and in silico analyses of foodborne pathogens from metagenomic samples

Journal Requirements:

When submitting your revision, we need you to address these additional requirements.

1. Please ensure that your manuscript meets PLOS ONE's style requirements, including those for file naming. The PLOS ONE style templates can be found at https://journals.plos.org/plosone/s/file?id=wjVg/PLOSOne_formatting_sample_main_body.pdf and https://journals.plos.org/plosone/s/file?id=ba62/PLOSOne_formatting_sample_title_authors_affiliations.pdf

 -ok

2. Please note that PLOS One has specific guidelines on code sharing for submissions in which author-generated code underpins the findings in the manuscript. In these cases, we expect all author-generated code to be made available without restrictions upon publication of the work. Please review our guidelines at https://journals.plos.org/plosone/s/materials-and-software-sharing#loc-sharing-code and ensure that your code is shared in a way that follows best practice and facilitates reproducibility and reuse.

 -ok

3. In your Methods section, please provide additional information regarding the permits you obtained for the work. Please ensure you have included the full name of the authority that approved the field site access and, if no permits were required, a brief statement explaining why.

 - Water samples were collected from various sites, and no permits were required because a member of the study team was affiliated with the relevant authority. All samples were prepared according to the established protocol

4. We note that the grant information you provided in the ‘Funding Information’ and ‘Financial Disclosure’ sections do not match.

- Will fix it when submitting.

When you resubmit, please ensure that you provide the correct grant numbers for the awards you received for your study in the ‘Funding Information’ section.

Ok.

5. Thank you for stating the following in the Acknowledgments Section of your manuscript:
The study was supported by funding from the Chief Scientist-Challenge Grants Proposal #2021-1464 and the FDA Foods Program Intramural Funds.

We note that you have provided funding information that is not currently declared in your Funding Statement. However, funding information should not appear in the Acknowledgments section or other areas of your manuscript. We will only publish funding information present in the Funding Statement section of the online submission form.
Please remove any funding-related text from the manuscript and let us know how you would like to update your Funding Statement. Currently, your Funding Statement reads as follows:
The author(s) received no specific funding for this work.

Please include your amended statements within your cover letter; we will change the online submission form on your behalf.

- Done. We have eliminated the funding information from the manuscript and have added the funding information. We could not modify the funding statement in the submission form. We have added the following to the cover letter: “The funders had no role in study design, data collection and analysis, decision to publish, or preparation of the manuscript”

6. Please amend your list of authors on the manuscript to ensure that each author is linked to an affiliation. Authors’ affiliations should reflect the institution where the work was done (if authors moved subsequently, you can also list the new affiliation stating “current affiliation:….” as necessary).

-ok. We all work in the same place but belong to different branches and divisions. We are all part of the Human Foods Program (HFP), Food and Drug Administration.

7. [Please include captions for your Supporting Information files at the end of your manuscript, and update any in-text citations to match accordingly. Please see our Supporting Information guidelines for more information: http://journals.plos.org/plosone/s/supporting-information.](http://journals.plos.org/plosone/s/supporting-information.)

 - done.

8. If the reviewer comments include a recommendation to cite specific previously published works, please review and evaluate these publications to determine whether they are relevant and should be cited. There is no requirement to cite these works unless the editor has indicated otherwise.

- ok. Done. No additional citations were recommended.

9. Please review your reference list to ensure that it is complete and correct. If you have cited papers that have been retracted, please include the rationale for doing so in the manuscript text, or remove these references and replace them with relevant current references. Any changes to the reference list should be mentioned in the rebuttal letter that accompanies your revised manuscript. If you need to cite a retracted article, indicate the article’s retracted status in the References list and also include a citation and full reference for the retraction notice.

- Not applicable.

Reviewer's Responses to Questions

**Comments to the Author**

Reviewer #1: This manuscript describes the development of a stand-alone open-source tool (centriflaken) for the automated detection of Shiga toxin-containing Escherichia coli (STEC) in metagenomic samples. Previously, the authors published the analysis pipeline, but each subsequential step in the pipeline had to be manually started. This manuscript compares and contrasts the previous pipeline with the new stand-alone version. The ability to parallelize the new version resulted in the analysis completing in about 1/3 the time it took the previous version and maintained the same accuracy. Another added feature is the use of singularity to package all the software with their dependencies which will help end-users install and run centriflaken. This new tool has the ability to help identify STEC in metagenomic samples taken from water if the concentration meets the minimum detection limit.

- Thank you for your thoughtful and comprehensive review of our manuscript. We appreciate your recognition of the key improvements in centriflaken, particularly the automation capabilities, parallelization benefits, and the singularity packaging for enhanced usability. Your acknowledgment of the tool's potential application for STEC detection in water samples is especially valuable, and we're grateful for your constructive feedback on this work.

Page 5, line 121. change “bysubtyping” to “by subtyping”.

-done.

Page 7, line 152. When starting a sentence, the “c” in centriflaken should be capitalized. Also, page 9, line 193, the “c” in centriflaken is capitalized. My guess is that it should be lowercase.

- Agreed and done.

Page 8, lines 176. The sentence “The pipeline took approximately 6 hours to finish….” Is a result in the Material and Methods section. There needs to be more information about the run including number of reads, total number of bases, was it run in parallel, what processors were used, and other spec of the compute system being used.

- I agreed. We decided to eliminate this sentence from the materials and methods section and move it to the results section, as described below in response to the Page 12, lines 265-268 comment.

Page 9, lines 193-195. Why isn’t more information given about using Illumina short reads with centriflaken? This is asking the reader to take your word that it works instead of showing the data. This is the perfect opportunity to show the process for using centriflaken with short read technologies.

- We appreciate the reviewer's valid concern regarding the limited information provided about using Illumina short reads with centriflaken. The reviewer is correct that additional data and demonstration would strengthen this manuscript. We acknowledge that the current presentation asks readers to accept our assertion without sufficient supporting evidence. While this is not the primary focus of this manuscript, we do have preliminary data on centriflaken's performance with Illumina short reads available on protocols.io. These results represent part of a separate, more comprehensive study using Illumina MiSeq reads that is currently in preparation for publication. This forthcoming study will provide detailed validation data, performance metrics, and comparative analyses specifically focused on short-read sequencing applications with centriflaken. To address the reviewer's concern in the current manuscript, we will add a supplementary MultiQC file showing results for 29 short-read samples with diverse levels of STECs and the following sentence: “An example of centriflaken's performance using Illumina short reads can be found in Supplementary MultiQC HTML Report 4.”

Page 9, lines 205-207. Please include more information about the samples used in the study. Did you re-extract DNA from the water sample, was the water sample frozen, was the DNA frozen from a previous extraction or did you use the reads generated from a previous Nanopore run?

- We thank the reviewer for requesting clarification about our sample preparation methodology. The 21 samples were collected in 2020 and 2021, and both sample processing and DNA extraction were performed immediately upon collection. Nanopore sequencing runs were conducted as soon as DNA was obtained. Due to the COVID-19 pandemic, our research focus was temporarily diverted to other studies, and we recently re-analyzed the existing sequencing data with centriflaken for this publication. To provide complete transparency regarding our experimental design, we have modified the sentence to include "(sample collection and processing detailed below)" as suggested by the reviewer. This clarifies that we used fresh samples with immediate processing and sequencing, rather than re-extracted DNA, frozen samples, or previously generated sequencing reads.

Page 9, line 212. I am not sure what the “X” means in the sentence.

- Thank you for your observation. That was a typo. We have eliminated the X.

Page 12, line 263. Change “provides” to “provided”.

- Agreed and done.

Page 12, lines 265-268. Please provide the more detail about the computer systems used to run centriflaken and the previous manual version.

- The reduction of time and computational resources mentioned in the paper are with respect to running them on our HPC Cluster. Both the individual steps that are part of the centriflaken workflow and the automated nextflow workflow were run on Reedling HPC Cluster which uses SLURM for job scheduling with each compute node containing 48 CPU cores with 256 GB of memory. Each of the individual steps of the centriflaken workflow were executed in batch mode (i.e., each command was written sequentially as a bash script). In contrast the Nextflow version automatically parallelizes the entire sequence of pipeline steps. We have added sentence addressing this: “Both the manual workflow and the automated Nextflow version were evaluated on the Reedling HPC Cluster, which uses SLURM for job scheduling with each compute node containing 48 CPU cores and 256 GB of memory. Each of the individual steps of the centriflaken workflow were executed in batch mode (i.e., each command was written sequentially as a bash script). In contrast the Nextflow version automatically parallelizes the entire sequence of pipeline steps.”

Page 12, lines 273-275. The sentences says that there were the “same” number of contigs between the previous version and centriflaken. While they are the same for two inoculation levels, they are similar for the lower inoculation level samples.

- Agreed and modified the sentence to read as: “but a similar number of contigs containing the STEC spiked genome was recovered (with minimal differences observed only at the two recoverable lowest inoculation levels), confirming the equivalency of both analysis workflows”

Table 1. There is a superscript “c” at the end of the Maguire et al 2021 column. It probably should be a superscript “a”.

- That is correct. Thank you for the correction. Done.

Page 13, lines 280-283. What is the concentration of pathogens found in food outbreak vectors? Is there 106 CFU/ml? When thinking of complex samples to extract DNA for metagenomic samples from, water would not be on the top of my list. How would this be used with other preharvest sample types. What happens when DNA is extracted from food stuffs like meat products or leafy greens where eukaryotic DNA can make up most of the extracted DNA. The usefulness of this tool is very apparent. I am just wondering how logistical it is to use with samples that have low pathogen numbers and is diluted in DNA from other organisms.

- The reviewer raises an important point about pathogen concentrations in real-world food samples. In FDA food safety protocols for STEC detection and isolation, an enrichment step is always performed prior to analysis. This enrichment process increases the target pathogen numbers from potentially very low initial concentrations (which may be below detection limits) to higher, detectable levels typically in the range we tested (10^6 CFU/ml or higher). Therefore, the concentrations we evaluated in this study reflect the post-enrichment levels that would be encountered in actual FDA food safety workflows, not the initial pathogen loads in raw food samples. The enrichment step is a standard and essential component of food pathogen detection protocols, ensuring that even samples with initially low pathogen numbers can be effectively analyzed. This makes centriflaken directly applicable to current FDA food safety practices, as it would be used on enriched samples where pathogen concentrations have been amplified to detectable levels. That is why the initial STEC qPCR of the enrichment is a very crucial step in the entire workflow, because that will provide the concentration of the target in the sample and the feasibility of closing or getting a fragmented STEC genome by long read sequencing. We have added this sentence after that statement: “In accordance with standard FDA food safety protocols for STECs, an enrichment step is performed prior to sequencing to amplify target pathogen concentrations from potentially low initial levels to the detectable range (≥10^6^ CFU/ml).”

Page 14, line 319. Again, please provide some type of context to the computers systems used.

- Agreed and done. We have added the following sentence: “centriflaken was run on a Reedling HPC Cluster, which uses SLURM for job scheduling with each compute node containing 48 CPU cores and 256 GB of memory.”

Tables 3 and 4. How would you interpret the results from sample FAQ33923 because it has stx2, eae, tir exhA, espA, espB, espD, espF and espI but there multiple E. coli serotype associated with sample. There are several serotypes identified in the serotype analysis that have been isolated from human infections including O104, 0113, and O45. Do you have enough sequence data to type the eae gene? I am afraid that the results from this sample could be also interpreted that eae and stx are located in isolates from different serotypes.

- We thank the reviewer for this insightful question. We have added a new explanation to the *Discussion* section to clarify the interpretation of sample FAQ33923. Specifically, we now note that this sample contained 26 distinct *E. coli* serotypes, including O104, O113, and O45—three serotypes commonly associated with human infections. Several virulence genes, including *stx2* and *eae*, were detected. The *eae* gene showed 99% identity to an *Escherichia albertii* intimin allele (epsilon-7/Xi allele, GenBank accession FJ609833.1), while *stx2* was located on a separate contig, suggesting that these genes originated from different strains within the same enrichment. Other virulence factors (*espA*, *espB*, *espD*, and *espF*) also matched *E. albertii*, supporting the interpretation that *eae* and its associated genes derive from a non-STEC background. Although *stx2* was clearly present, the high degree of genomic fragmentation and diversity of *E. coli* serotypes prevented conclusive linkage between *stx2* and *eae*. This case exemplifies the challenge of analyzing highly mixed metagenomic samples where multiple closely related *E. coli* strains coexist, underscoring the limitations of metagenomic reconstruction in resolving the genomic context of virulence determinants.

Tables. Please list the samples in the Tables in the same order.

- Agreed and done. We thank the reviewer for their excellent suggestion.

Page 16, lines 252-254. qPCR was mentioned in the Materials and Methods but not in the results.

- We thank the reviewer for this helpful comment. We have added a description of the qPCR results in the Results section as follows: “qPCR screening of these 21 enriched samples confirmed the presence of *stx* (17/21) and O157 *wzy* (12/21) genes. The results are summarized in Table 3. Four samples tested negative for either *stx* or O157 *wzy* genes and were used as negative control for evaluating the centriflaken pipeline”. The results are summarized in a new Table (**Table 3)**, and four samples that tested negative for either stx or wzy were used as negative controls for evaluating the centriflaken pipeline.

It would be good to have a Figure showing the complete fast metagenomic analysis procedure including culturing.

- We agree with the reviewer’s suggestion. We have added a new figure (**Figure 3**) illustrating the complete fast metagenomic analysis workflow, starting from sample collection and culturing to final reporting. This figure provides a clear visual overview of the end-to-end process described in the manuscript.

Supplemental Table 3. The genes under the macrolide column needs to be italicized.

- Agreed and done.
