# Supplementary material for: Centriflaken: An automated data analysis pipeline for assembly and in silico analyses of foodborne pathogens from metagenomic samples
Source: PLoS One. 2025 Dec 23;20(12):e0329425. doi: 10.1371/journal.pone.0329425 (PMC12725755; doi:10.1371/journal.pone.0329425)
Supplement: Peer review history

# Peer review: Decision Letter 0

**DOI:** 10.1371/journal.pone.0329425.r001

**Authors:** Mark Eppinger

11 Sep 2025

Dear Dr. González-Escalona,

Thank you for submitting your manuscript to PLOS ONE. After careful consideration, we feel that it has merit but does not fully meet PLOS ONE’s publication criteria as it currently stands. Therefore, we invite you to submit a revised version of the manuscript that addresses the points raised during the review process.

Please submit your revised manuscript by Oct 26 2025 11:59PM. If you will need more time than this to complete your revisions, please reply to this message or contact the journal office at plosone@plos.org . When you're ready to submit your revision, log on to https://www.editorialmanager.com/pone/ and select the 'Submissions Needing Revision' folder to locate your manuscript file.

- A rebuttal letter that responds to each point raised by the academic editor and reviewer(s). You should upload this letter as a separate file labeled 'Response to Reviewers'.
- A marked-up copy of your manuscript that highlights changes made to the original version. You should upload this as a separate file labeled 'Revised Manuscript with Track Changes'.
- An unmarked version of your revised paper without tracked changes. You should upload this as a separate file labeled 'Manuscript'.

If applicable, we recommend that you deposit your laboratory protocols in protocols.io to enhance the reproducibility of your results. Protocols.io assigns your protocol its own identifier (DOI) so that it can be cited independently in the future. For instructions see: https://journals.plos.org/plosone/s/submission-guidelines#loc-laboratory-protocols . Additionally, PLOS ONE offers an option for publishing peer-reviewed Lab Protocol articles, which describe protocols hosted on protocols.io. Read more information on sharing protocols at https://plos.org/protocols?utm_medium=editorial-email&utm_source=authorletters&utm_campaign=protocols .

We look forward to receiving your revised manuscript.

Kind regards,

Mark Eppinger

Academic Editor

PLOS ONE

Journal Requirements:

When submitting your revision, we need you to address these additional requirements.

1. Please ensure that your manuscript meets PLOS ONE's style requirements, including those for file naming. The PLOS ONE style templates can be found at https://journals.plos.org/plosone/s/file?id=wjVg/PLOSOne_formatting_sample_main_body.pdf and https://journals.plos.org/plosone/s/file?id=ba62/PLOSOne_formatting_sample_title_authors_affiliations.pdf

2. Please note that PLOS One has specific guidelines on code sharing for submissions in which author-generated code underpins the findings in the manuscript. In these cases, we expect all author-generated code to be made available without restrictions upon publication of the work. Please review our guidelines at https://journals.plos.org/plosone/s/materials-and-software-sharing#loc-sharing-code and ensure that your code is shared in a way that follows best practice and facilitates reproducibility and reuse.

3. In your Methods section, please provide additional information regarding the permits you obtained for the work. Please ensure you have included the full name of the authority that approved the field site access and, if no permits were required, a brief statement explaining why.

4. We note that the grant information you provided in the ‘Funding Information’ and ‘Financial Disclosure’ sections do not match.

When you resubmit, please ensure that you provide the correct grant numbers for the awards you received for your study in the ‘Funding Information’ section.

5. Thank you for stating the following in the Acknowledgments Section of your manuscript:

The study was supported by funding from the Chief Scientist-Challenge Grants Proposal #2021-1464 and the FDA Foods Program Intramural Funds.

We note that you have provided funding information that is not currently declared in your Funding Statement. However, funding information should not appear in the Acknowledgments section or other areas of your manuscript. We will only publish funding information present in the Funding Statement section of the online submission form.

Please remove any funding-related text from the manuscript and let us know how you would like to update your Funding Statement. Currently, your Funding Statement reads as follows:

The author(s) received no specific funding for this work.

Please include your amended statements within your cover letter; we will change the online submission form on your behalf.

6. Please amend your list of authors on the manuscript to ensure that each author is linked to an affiliation. Authors’ affiliations should reflect the institution where the work was done (if authors moved subsequently, you can also list the new affiliation stating “current affiliation:….” as necessary).

7. Please include captions for your Supporting Information files at the end of your manuscript, and update any in-text citations to match accordingly. Please see our Supporting Information guidelines for more information: http://journals.plos.org/plosone/s/supporting-information.

8. If the reviewer comments include a recommendation to cite specific previously published works, please review and evaluate these publications to determine whether they are relevant and should be cited. There is no requirement to cite these works unless the editor has indicated otherwise. 

9. Please review your reference list to ensure that it is complete and correct. If you have cited papers that have been retracted, please include the rationale for doing so in the manuscript text, or remove these references and replace them with relevant current references. Any changes to the reference list should be mentioned in the rebuttal letter that accompanies your revised manuscript. If you need to cite a retracted article, indicate the article’s retracted status in the References list and also include a citation and full reference for the retraction notice.

[Note: HTML markup is below. Please do not edit.]

Reviewers' comments:

Reviewer's Responses to Questions

**Comments to the Author**

1. Is the manuscript technically sound, and do the data support the conclusions?

Reviewer #1: Yes

**********

2. Has the statistical analysis been performed appropriately and rigorously?

Reviewer #1: N/A

**********

3. Have the authors made all data underlying the findings in their manuscript fully available?

The PLOS Data policy

Reviewer #1: Yes

**********

4. Is the manuscript presented in an intelligible fashion and written in standard English?

Reviewer #1: Yes

**********

Reviewer #1: This manuscript describes the development of a stand-alone open-source tool (centriflaken) for the automated detection of Shiga toxin-containing Escherichia coli (STEC) in metagenomic samples. Previously, the authors published the analysis pipeline, but each subsequential step in the pipeline had to be manually started. This manuscript compares and contrasts the previous pipeline with the new stand-alone version. The ability to parallelize the new version resulted in the analysis completing in about 1/3 the time it took the previous version and maintained the same accuracy. Another added feature is the use of singularity to package all the software with their dependencies which will help end-users install and run centriflaken. This new tool has the ability to help identify STEC in metagenomic samples taken from water if the concentration meets the minimum detection limit.

Questions/comments

Page 5, line 121. change “bysubtyping” to “by subtyping”.

Page 7, line 152. When starting a sentence, the “c” in centriflaken should be capitalized. Also, page 9, line 193, the “c” in centriflaken is capitalized. My guess is that it should be lowercase.

Page 8, lines 176. The sentence “The pipeline took approximately 6 hours to finish….” Is a result in the Material and Methods section. There needs to be more information about the run including number of reads, total number of bases, was it run in parallel, what processors were used, and other spec of the compute system being used.

Page 9, lines 193-195. Why isn’t more information given about using Illumina short reads with centriflaken? This is asking the reader to take your word that it works instead of showing the data. This is the perfect opportunity to show the process for using centriflaken with short read technologies.

Page 9, lines 205-207. Please include more information about the samples used in the study. Did you re-extract DNA from the water sample, was the water sample frozen, was the DNA frozen from a previous extraction or did you use the reads generated from a previous Nanopore run?

Page 9, line 212. I am not sure what the “X” means in the sentence.

Page 12, line 263. Change “provides” to “provided”.

Page 12, lines 265-268. Please provide the more detail about the computer systems used to run centriflaken and the previous manual version.

Page 12, lines 273-275. The sentences says that there were the “same” number of contigs between the previous version and centriflaken. While they are the same for two inoculation levels, they are similar for the lower inoculation level samples.

Table 1. There is a superscript “c” at the end of the Maguire et al 2021 column. It probably should be a superscript “a”.

Page 13, lines 280-283. What is the concentration of pathogens found in food outbreak vectors? Is there 106 CFU/ml? When thinking of complex samples to extract DNA for metagenomic samples from, water would not be on the top of my list. How would this be used with other preharvest sample types. What happens when DNA is extracted from food stuffs like meat products or leafy greens where eukaryotic DNA can make up most of the extracted DNA. The usefulness of this tool is very apparent. I am just wondering how logistical it is to use with samples that have low pathogen numbers and is diluted in DNA from other organisms.

Page 14, line 319. Again, please provide some type of context to the computers systems used.

Tables 3 and 4. How would you interpret the results from sample FAQ33923 because it has stx2, eae, tir exhA, espA, espB, espD, espF and espI but there multiple E. coli serotype associated with sample. There are several serotypes identified in the serotype analysis that have been isolated from human infections including O104, 0113, and O45. Do you have enough sequence data to type the eae gene? I am afraid that the results from this sample could be also interpreted that eae and stx are located in isolates from different serotypes.

Tables. Please list the samples in the Tables in the same order.

Page 16, lines 252-254. qPCR was mentioned in the Materials and Methods but not in the results. It would be good to have a Figure showing the complete fast metagenomic analysis procedure including culturing.

Supplemental Table 3. The genes under the macrolide column needs to be italicized.

**********

what does this mean? ). If published, this will include your full peer review and any attached files.

If you choose “no”, your identity will remain anonymous but your review may still be made public.

**Do you want your identity to be public for this peer review?** For information about this choice, including consent withdrawal, please see our Privacy Policy

Reviewer #1: No

**********

[NOTE: If reviewer comments were submitted as an attachment file, they will be attached to this email and accessible via the submission site. Please log into your account, locate the manuscript record, and check for the action link "View Attachments". If this link does not appear, there are no attachment files.]

While revising your submission, please upload your figure files to the Preflight Analysis and Conversion Engine (PACE) digital diagnostic tool, https://pacev2.apexcovantage.com/ . PACE helps ensure that figures meet PLOS requirements. To use PACE, you must first register as a user. Registration is free. Then, login and navigate to the UPLOAD tab, where you will find detailed instructions on how to use the tool. If you encounter any issues or have any questions when using PACE, please email PLOS at figures@plos.org . Please note that Supporting Information files do not need this step.

# Author comment: Author response to Decision Letter 1

**DOI:** 10.1371/journal.pone.0329425.r002

19 Nov 2025

Reviewer's Responses to Questions

Comments to the Author

Reviewer #1: This manuscript describes the development of a stand-alone open-source tool (centriflaken) for the automated detection of Shiga toxin-containing Escherichia coli (STEC) in metagenomic samples. Previously, the authors published the analysis pipeline, but each subsequential step in the pipeline had to be manually started. This manuscript compares and contrasts the previous pipeline with the new stand-alone version. The ability to parallelize the new version resulted in the analysis completing in about 1/3 the time it took the previous version and maintained the same accuracy. Another added feature is the use of singularity to package all the software with their dependencies which will help end-users install and run centriflaken. This new tool has the ability to help identify STEC in metagenomic samples taken from water if the concentration meets the minimum detection limit.

- Thank you for your thoughtful and comprehensive review of our manuscript. We appreciate your recognition of the key improvements in centriflaken, particularly the automation capabilities, parallelization benefits, and the singularity packaging for enhanced usability. Your acknowledgment of the tool's potential application for STEC detection in water samples is especially valuable, and we're grateful for your constructive feedback on this work.

Page 5, line 121. change “bysubtyping” to “by subtyping”.

-done.

Page 7, line 152. When starting a sentence, the “c” in centriflaken should be capitalized. Also, page 9, line 193, the “c” in centriflaken is capitalized. My guess is that it should be lowercase.

- Agreed and done.

Page 8, lines 176. The sentence “The pipeline took approximately 6 hours to finish….” Is a result in the Material and Methods section. There needs to be more information about the run including number of reads, total number of bases, was it run in parallel, what processors were used, and other spec of the compute system being used.

- I agreed. We decided to eliminate this sentence from the materials and methods section and move it to the results section, as described below in response to the Page 12, lines 265-268 comment.

Page 9, lines 193-195. Why isn’t more information given about using Illumina short reads with centriflaken? This is asking the reader to take your word that it works instead of showing the data. This is the perfect opportunity to show the process for using centriflaken with short read technologies.

- We appreciate the reviewer's valid concern regarding the limited information provided about using Illumina short reads with centriflaken. The reviewer is correct that additional data and demonstration would strengthen this manuscript. We acknowledge that the current presentation asks readers to accept our assertion without sufficient supporting evidence. While this is not the primary focus of this manuscript, we do have preliminary data on centriflaken's performance with Illumina short reads available on protocols.io. These results represent part of a separate, more comprehensive study using Illumina MiSeq reads that is currently in preparation for publication. This forthcoming study will provide detailed validation data, performance metrics, and comparative analyses specifically focused on short-read sequencing applications with centriflaken. To address the reviewer's concern in the current manuscript, we will add a supplementary MultiQC file showing results for 29 short-read samples with diverse levels of STECs and the following sentence: “An example of centriflaken's performance using Illumina short reads can be found in Supplementary MultiQC HTML Report 4.”

Page 9, lines 205-207. Please include more information about the samples used in the study. Did you re-extract DNA from the water sample, was the water sample frozen, was the DNA frozen from a previous extraction or did you use the reads generated from a previous Nanopore run?

- We thank the reviewer for requesting clarification about our sample preparation methodology. The 21 samples were collected in 2020 and 2021, and both sample processing and DNA extraction were performed immediately upon collection. Nanopore sequencing runs were conducted as soon as DNA was obtained. Due to the COVID-19 pandemic, our research focus was temporarily diverted to other studies, and we recently re-analyzed the existing sequencing data with centriflaken for this publication. To provide complete transparency regarding our experimental design, we have modified the sentence to include "(sample collection and processing detailed below)" as suggested by the reviewer. This clarifies that we used fresh samples with immediate processing and sequencing, rather than re-extracted DNA, frozen samples, or previously generated sequencing reads.

Page 9, line 212. I am not sure what the “X” means in the sentence.

- Thank you for your observation. That was a typo. We have eliminated the X.

Page 12, line 263. Change “provides” to “provided”.

- Agreed and done.

Page 12, lines 265-268. Please provide the more detail about the computer systems used to run centriflaken and the previous manual version.

- The reduction of time and computational resources mentioned in the paper are with respect to running them on our HPC Cluster. Both the individual steps that are part of the centriflaken workflow and the automated nextflow workflow were run on Reedling HPC Cluster which uses SLURM for job scheduling with each compute node containing 48 CPU cores with 256 GB of memory. Each of the individual steps of the centriflaken workflow were executed in batch mode (i.e., each command was written sequentially as a bash script). In contrast the Nextflow version automatically parallelizes the entire sequence of pipeline steps. We have added sentence addressing this: “Both the manual workflow and the automated Nextflow version were evaluated on the Reedling HPC Cluster, which uses SLURM for job scheduling with each compute node containing 48 CPU cores and 256 GB of memory. Each of the individual steps of the centriflaken workflow were executed in batch mode (i.e., each command was written sequentially as a bash script). In contrast the Nextflow version automatically parallelizes the entire sequence of pipeline steps.”

Page 12, lines 273-275. The sentences says that there were the “same” number of contigs between the previous version and centriflaken. While they are the same for two inoculation levels, they are similar for the lower inoculation level samples.

- Agreed and modified the sentence to read as: “but a similar number of contigs containing the STEC spiked genome was recovered (with minimal differences observed only at the two recoverable lowest inoculation levels), confirming the equivalency of both analysis workflows”

Table 1. There is a superscript “c” at the end of the Maguire et al 2021 column. It probably should be a superscript “a”.

- That is correct. Thank you for the correction. Done.

Page 13, lines 280-283. What is the concentration of pathogens found in food outbreak vectors? Is there 106 CFU/ml? When thinking of complex samples to extract DNA for metagenomic samples from, water would not be on the top of my list. How would this be used with other preharvest sample types. What happens when DNA is extracted from food stuffs like meat products or leafy greens where eukaryotic DNA can make up most of the extracted DNA. The usefulness of this tool is very apparent. I am just wondering how logistical it is to use with samples that have low pathogen numbers and is diluted in DNA from other organisms.

- The reviewer raises an important point about pathogen concentrations in real-world food samples. In FDA food safety protocols for STEC detection and isolation, an enrichment step is always performed prior to analysis. This enrichment process increases the target pathogen numbers from potentially very low initial concentrations (which may be below detection limits) to higher, detectable levels typically in the range we tested (10^6 CFU/ml or higher). Therefore, the concentrations we evaluated in this study reflect the post-enrichment levels that would be encountered in actual FDA food safety workflows, not the initial pathogen loads in raw food samples. The enrichment step is a standard and essential component of food pathogen detection protocols, ensuring that even samples with initially low pathogen numbers can be effectively analyzed. This makes centriflaken directly applicable to current FDA food safety practices, as it would be used on enriched samples where pathogen concentrations have been amplified to detectable levels. That is why the initial STEC qPCR of the enrichment is a very crucial step in the entire workflow, because that will provide the concentration of the target in the sample and the feasibility of closing or getting a fragmented STEC genome by long read sequencing. We have added this sentence after that statement: “In accordance with standard FDA food safety protocols for STECs, an enrichment step is performed prior to sequencing to amplify target pathogen concentrations from potentially low initial levels to the detectable range (≥106 CFU/ml).”

Page 14, line 319. Again, please provide some type of context to the computers systems used.

- Agreed and done. We have added the following sentence: “centriflaken was run on a Reedling HPC Cluster, which uses SLURM for job scheduling with each compute node containing 48 CPU cores and 256 GB of memory.”

Tables 3 and 4. How would you interpret the results from sample FAQ33923 because it has stx2, eae, tir exhA, espA, espB, espD, espF and espI but there multiple E. coli serotype associated with sample. There are several serotypes identified in the serotype analysis that have been isolated from human infections including O104, 0113, and O45. Do you have enough sequence data to type the eae gene? I am afraid that the results from this sample could be also interpreted that eae and stx are located in isolates from different serotypes.

- We thank the reviewer for this insightful question. We have added a new explanation to the Discussion section to clarify the interpretation of sample FAQ33923. Specifically, we now note that this sample contained 26 distinct E. coli serotypes, including O104, O113, and O45—three serotypes commonly associated with human infections. Several virulence genes, including stx2 and eae, were detected. The eae gene showed 99% identity to an Escherichia albertii intimin allele (epsilon-7/Xi allele, GenBank accession FJ609833.1), while stx2 was located on a separate contig, suggesting that these genes originated from different strains within the same enrichment. Other virulence factors (espA, espB, espD, and espF) also matched E. albertii, supporting the interpretation that eae and its associated genes derive from a non-STEC background. Although stx2 was clearly present, the high degree of genomic fragmentation and diversity of E. coli serotypes prevented conclusive linkage between stx2 and eae. This case exemplifies the challenge of analyzing highly mixed metagenomic samples where multiple closely related E. coli strains coexist, underscoring the limitations of metagenomic reconstruction in resolving the genomic context of virulence determinants.

Tables. Please list the samples in the Tables in the same order.

- Agreed and done. We thank the reviewer for their excellent suggestion.

Page 16, lines 252-254. qPCR was mentioned in the Materials and Methods but not in the results.

- We thank the reviewer for this helpful comment. We have added a description of the qPCR results in the Results section as follows: “qPCR screening of these 21 enriched samples confirmed the presence of stx (17/21) and O157 wzy (12/21) genes. The results are summarized in Table 3. Four samples tested negative for either stx or O157 wzy genes and were used as negative control for evaluating the centriflaken pipeline”. The results are summarized in a new Table (Table 3), and four samples that tested negative for either stx or wzy were used as negative controls for evaluating the centriflaken pipeline.

It would be good to have a Figure showing the complete fast metagenomic analysis procedure including culturing.

- We agree with the reviewer’s suggestion. We have added a new figure (Figure 3) illustrating the complete fast metagenomic analysis workflow, starting from sample collection and culturing to final reporting. This figure provides a clear visual overview of the end-to-end process described in the manuscript.

Supplemental Table 3. The genes under the macrolide column needs to be italicized.

- Agreed and done.

Attachment Submitted filename: Response to Reviewers PONE-D-25-38731.docx

# Editor report: Decision Letter 1

**DOI:** 10.1371/journal.pone.0329425.r003

**Authors:** Mark Eppinger

25 Nov 2025

centriflaken: an automated data analysis pipeline for assembly and in silico analyses of foodborne pathogens from metagenomic samples

PONE-D-25-38731R1

Dear Dr. González-Escalona,

We’re pleased to inform you that your manuscript has been judged scientifically suitable for publication and will be formally accepted for publication once it meets all outstanding technical requirements.

Within one week, you’ll receive an e-mail detailing the required amendments. When these have been addressed, you’ll receive a formal acceptance letter and your manuscript will be scheduled for publication.

An invoice will be generated when your article is formally accepted. Please note, if your institution has a publishing partnership with PLOS and your article meets the relevant criteria, all or part of your publication costs will be covered. Please make sure your user information is up-to-date by logging into Editorial Manager at Editorial Manager®  and clicking the ‘Update My Information' link at the top of the page. For questions related to billing, please contact billing support .

If your institution or institutions have a press office, please notify them about your upcoming paper to help maximize its impact. If they’ll be preparing press materials, please inform our press team as soon as possible -- no later than 48 hours after receiving the formal acceptance. Your manuscript will remain under strict press embargo until 2 pm Eastern Time on the date of publication. For more information, please contact onepress@plos.org.

Kind regards,

Mark Eppinger

Academic Editor

PLOS ONE

Additional Editor Comments (optional):

Reviewers' comments:

# Editor report: Acceptance letter

**DOI:** 10.1371/journal.pone.0329425.r004

**Authors:** Mark Eppinger

PONE-D-25-38731R1

PLOS One

Dear Dr. González-Escalona,

I'm pleased to inform you that your manuscript has been deemed suitable for publication in PLOS One. Congratulations! Your manuscript is now being handed over to our production team.

At this stage, our production department will prepare your paper for publication. This includes ensuring the following:

* All references, tables, and figures are properly cited

* All relevant supporting information is included in the manuscript submission,

* There are no issues that prevent the paper from being properly typeset

You will receive further instructions from the production team, including instructions on how to review your proof when it is ready. Please keep in mind that we are working through a large volume of accepted articles, so please give us a few days to review your paper and let you know the next and final steps.

Lastly, if your institution or institutions have a press office, please let them know about your upcoming paper now to help maximize its impact. If they'll be preparing press materials, please inform our press team within the next 48 hours. Your manuscript will remain under strict press embargo until 2 pm Eastern Time on the date of publication. For more information, please contact onepress@plos.org.

You will receive an invoice from PLOS for your publication fee after your manuscript has reached the completed accept phase. If you receive an email requesting payment before acceptance or for any other service, this may be a phishing scheme. Learn how to identify phishing emails and protect your accounts at https://explore.plos.org/phishing.

If we can help with anything else, please email us at customercare@plos.org.

Thank you for submitting your work to PLOS ONE and supporting open access.

Kind regards,

PLOS ONE Editorial Office Staff

on behalf of

Dr. Mark Eppinger

Academic Editor

PLOS One
